# Evolutionary instability of selfish learning in repeated games

**DOI:** 10.1093/pnasnexus/pgac141

**Published:** 2022-07-27

**Authors:** Alex McAvoy, Julian Kates-Harbeck, Krishnendu Chatterjee, Christian Hilbe

**Affiliations:** Department of Mathematics, University of Pennsylvania, Philadelphia, PA, USA; Center for Mathematical Biology, University of Pennsylvania, Philadelphia, PA, USA; Department of Physics, Harvard University, Cambridge, MA, USA; Institute of Science and Technology Austria, Klosterneuburg, Austria; Max Planck Research Group: Dynamics of Social Behavior, Max Planck Institute for Evolutionary Biology, Plön, Germany

## Abstract

Across many domains of interaction, both natural and artificial, individuals use past experience to shape future behaviors. The results of such learning processes depend on what individuals wish to maximize. A natural objective is one’s own success. However, when two such “selfish” learners interact with each other, the outcome can be detrimental to both, especially when there are conflicts of interest. Here, we explore how a learner can align incentives with a selfish opponent. Moreover, we consider the dynamics that arise when learning rules themselves are subject to evolutionary pressure. By combining extensive simulations and analytical techniques, we demonstrate that selfish learning is unstable in most classical two-player repeated games. If evolution operates on the level of long-run payoffs, selection instead favors learning rules that incorporate social (other-regarding) preferences. To further corroborate these results, we analyze data from a repeated prisoner’s dilemma experiment. We find that selfish learning is insufficient to explain human behavior when there is a trade-off between payoff maximization and fairness.

Significance Statement A natural first approach to learning is to attempt to improve one’s own outcome (e.g. wealth, resources, or reputation), without regard for others. This kind of “selfish” learning, however, can be detrimental in social dilemmas, in which the individuals’ incentives are at odds. Here, we study the evolutionary dynamics of different learning rules, demonstrating that selfish learning can be driven to extinction in evolving populations. To this end, we contrast selfish learning with a competing learning rule, which uses simple social preferences. The competing rule attains superior outcomes across a wide range of social interactions, even when interacting with selfish learners, and it ensures that these outcomes are both fair and socially optimal.

## Introduction

Individuals naturally adapt to their environment, either by modifying their existing behaviors or by considering alternative ones when necessary (1, 2). The study of behavioral adaptation is far-reaching, with applications ranging from microbial dynamics (3,4) to social preferences in humans (5–7) to learning algorithms in multiagent systems (8, 9). Evolutionary game theory, a tool for modeling behavioral adaptations (10–17), has been used to describe how people learn to engage in reciprocity (18–30), how social norms evolve over time (31,32), how groups are formed (33), how thriving communities can suddenly be undermined by corruption (34,35), and how artificial agents behave “in silico” (36–39). A key assumption in evolutionary game theory is that individuals might not act optimally from the outset. Rather, adaptation happens over time through either cultural or genetic mechanisms (10–15).

To describe how people learn new behaviors, the respective literature has considered various cognitive processes. Some models stipulate that individuals learn by imitating their peers (40–42), whereas others assume that learning is based on aspiration levels (43,44), reinforcement (45, 46), or introspection (47, 48). Crucially, however, learning rules are frequently based on the assumption that individuals strive to increase their own immediate payoffs. Although errors, mutations, and chance events may temporarily lead individuals to adopt inferior strategies, better performing strategies are favored on average. We refer to learning rules with this property as “selfish learning.”

In reality, many models ask neither whether individuals actually learn based on strict payoff maximization nor whether they have a long-run incentive to do so (40–48). This assumption might be justified when interactions lack any strategic component (as in single-player optimization). However, the rationale for selfish learning is less clear in social dilemmas, where there are conflicts of interest between the individual and the group (49–51). When social dilemmas give rise to multiple equilibria, selfish optimization may easily result in detrimental outcomes that are socially inefficient. This drawback is already well-recognized in the field of multiagent reinforcement learning, where selfish learners are considered “naïve” and serve as a benchmark for other learning rules (52). If selection acts upon the learning rules that determine how players choose strategies, then it may select for different learning rules altogether.

The problem of optimal learning is best illustrated with a key model of evolutionary game theory: the repeated prisoner’s dilemma (19). In each round of the game, two players independently choose whether to cooperate or defect. Each player has an incentive to defect even though mutual cooperation is in everybody’s interest. For repeated games with sufficiently long time horizons, there is a wide range of possible equilibria (53). In particular, players may always cooperate, always defect, or alternate between cooperation and defection (54). After decades of research, much is known about particular strategies that sustain cooperation, such as “tit-for-tat” (55) or “win-stay, lose-shift” (36). Much less is known how such desirable strategies can be learned in the first place, especially when players are learning concurrently. If strategy updating is described by imitation, for example, players may end up defecting for a substantial amount of time (56). Such detrimental outcomes become increasingly likely when the game involves only a few rounds, offers a small benefit of cooperation, or when players commit errors (21).

In this study, we ask whether there is a learning rule that helps individuals find more profitable equilibria, even when the opponent is self-interested. To this end, we imagine two individuals interacting in a repeated game. Each individual has its own learning rule for adapting its strategy, with the ultimate goal of achieving a high payoff. As a baseline, we consider a variant of selfish learning. At regular time intervals, a selfish learner compares the performance of its present strategy with the (hypothetical) performance of a slightly perturbed version of its strategy. The learner adopts the perturbed strategy if it yields a higher payoff, regardless of its effects on others.

We contrast selfish learning with a learning rule we term “fairness-mediated team learning” (*FMTL*). Players with this learning rule balance two objectives, efficiency and fairness. To promote efficiency, *FMTL* favors strategies that increase the total payoff of the group (i.e. the “team” payoff). In this way, *FMTL* aims to avoid equilibria that leave everybody worse off. To prevent themselves from getting exploited, however, *FMTL* players simultaneously aim to minimize payoff differences within their group (i.e. promote “fairness”). The respective weights assigned to efficiency and fairness are dynamically adjusted based on the players’ current payoffs. Increasing efficiency is the primary objective when payoff inequalities are negligible.

The definition of *FMTL* has a natural connection to the field of multiagent learning (57–61). Two of the most well-studied areas involve so-called “fully-cooperative” and “fully-competitive” interactions. In fully cooperative interactions, players have identical payoff functions; what is good for one player is equally good for the other. In fully competitive settings, the players’ incentives are perfectly opposed. The iterated prisoner’s dilemma falls somewhere in between and is often referred to as a “mixed” or “general-sum” game (62,63). As such, it presents a more difficult learning problem. In striving for fairness, *FMTL* forces the players to have approximately equal payoffs, which is reminiscent of the fully cooperative setting. Once payoffs are sufficiently close, *FMTL* views itself and the opponent as a team and attempts to optimize the total payoff. The approach of optimizing a team score is common in cooperative settings, especially when individuals have mostly (but not completely) aligned incentives (9).

When *all* learners are driven by fairness and efficiency, it may not be surprising that the learning dynamics favor socially beneficial outcomes. Remarkably, however, such outcomes already arise when only *one* learner has these objectives. For a wide range of two-player games, we show that *FMTL* players tend to settle at equilibria that are both individually optimal and socially efficient even when interacting with selfish opponents. Based on these observations, we explore the dynamics that arise when the two learning rules themselves are subject to evolution. We consider a process with two timescales. On a short timescale, individuals have a fixed learning rule that they use to guide their strategic choices. On a longer timescale, players can switch their learning rule, based on how successful it proved to be. The resulting evolutionary dynamics depend on which game is played, and on the relative pace at which learning rules are updated (compared to how often strategies are updated). When learning rules and strategies evolve at a similar timescale, selfish learning is favored. However, when learning rules evolve at a slower rate, selfish learning is routinely invaded and ceases to be stable in many classical games.

## Results

### A model of learning in repeated games

**Fig. 1.**
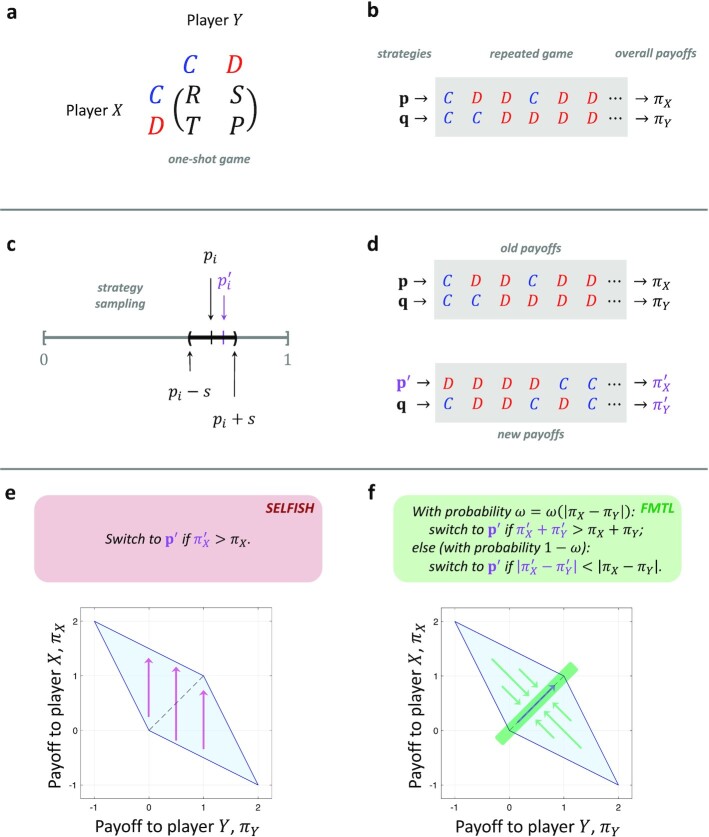
Learning in repeated games and *FMTL*. (a) In each encounter of a repeated game, players engage in a “one-shot” game. Both choose actions, *C* or *D*, and receive payoffs, *R, S, T*, or *P*. (b) A repeated game consists of a sequence of one-shot games. Both players choose strategies, $\mathbf {p}$ and $\mathbf {q}$, and receive overall payoffs, π_*X*_ and π_*Y*_, respectively. (c) To update strategies, players occasionally sample a new, nearby strategy $\mathbf {p^{\prime }}$ (“local search”). (d) When *X* uses strategy $\mathbf {p^{\prime }}$ against *Y*’s strategy, $\mathbf {q}$, the players get new payoffs, $\pi _{X}^{\prime }$ and $\pi _{Y}^{\prime }$. This new strategy is then evaluated based on a player’s learning rule. (e) If *X* is a selfish learner, $\mathbf {p^{\prime }}$ is accepted only if it improves *X*’s payoff, i.e. if $\pi _{X}^{\prime }\gt \pi _{X}$. (f) If *X* uses *FMTL*, then with probability 1 − ω she takes $\mathbf {p^{\prime }}$ only if it brings the two players’ payoffs closer together (fairness). Otherwise, with probability ω, she takes $\mathbf {p^{\prime }}$ only if it improves the sum of the two players’ payoffs (efficiency). The probability ω is a decreasing function of the payoff difference so that fairness becomes more important to *FMTL* as one player starts to do better than the other.

To compare different learning rules, we consider two players who interact in a repeated game. In each round, players independently decide whether to cooperate or defect. As a result, each player obtains a payoff (Fig. 1a). After every round, players interact for another round with probability λ. They decide whether to cooperate based on their strategies. A strategy is a rule that tells the player what to do in the next round, given the history of previous play. In the simplest version of the model, players use memory-one strategies (13). These strategies are contingent on only the last round of play, and they reasonably approximate human behavior in economic experiments (64–67). A memory-one strategy for player *X* consists of an initial probability of cooperation, *p*_0_, together with a four-tuple of conditional cooperation probabilities, $\mathbf {p}=\left(p_{CC},p_{CD},p_{DC},p_{DD}\right)\in \left[0,1\right]^{4}$. Here, *p_xy_* is the probability that *X* cooperates in the next round when *X* played *x* and *Y* played *y* in the previous round. For example, an unconditional defector is represented by a memory-one strategy with *p*_0_ = 0 and $\mathbf {p}=\left(0,0,0,0\right)$. Tit-for-tat takes the form *p*_0_ = 1 and $\mathbf {p}=\left(1,0,1,0\right)$. Given strategies $\mathbf {p}$ and $\mathbf {q}$ of the two players, we can compute how likely they are to cooperate over the course of the game, and which overall payoffs $\pi _{X}\left(\mathbf {p},\mathbf {q}\right)$ and $\pi _{Y}\left(\mathbf {p},\mathbf {q}\right)$ they obtain (Fig. 1b and “Methods”).

After each repeated game, players may update their strategies based on their experience with the opponent. They do so by implementing a learning rule. We consider learning rules that consist of four elements: a distribution $\mathcal {D}$ over initial strategies, a sampling procedure $\mathcal {S}$, a set of objective functions $\mathcal {V}$, and a priority assignment Ω. The first element, the distribution over initial strategies, determines the player’s default strategy that is used before any learning takes place. The second element, the sampling procedure, determines how to generate an alternative strategy in each learning step. Throughout the main text, we assume that a player *X* with current strategy $\mathbf {p}$ generates an alternative strategy $\mathbf {p^{\prime }}$ using local random search (68) (Fig. 1c). After generating an alternative strategy, the player decides whether to accept it. To this end, the set of objective functions $\mathcal {V}$ specifies what the player’s strategy ought to maximize. If *X*’s objective is to maximize $V\in \mathcal {V}$, the player switches to $\mathbf {p^{\prime }}$ if and only if $V\left(\mathbf {p^{\prime }},\mathbf {q}\right) \gt V\left(\mathbf {p},\mathbf {q}\right)$. This decision may involve, for example, a comparison between the player’s current payoff, $\pi _{X}\left(\mathbf {p},\mathbf {q}\right)$, and the payoff $\pi _{X}\left(\mathbf {p^{\prime }},\mathbf {q}\right)$ the player could have obtained using the alternative strategy (Fig. 1d). However, a player’s priorities over her objectives may change over time. The priority assignment $\Omega \left(\mathbf {p},\mathbf {q}\right)$ determines with which probability each objective $V\in \mathcal {V}$ is chosen.

We compare the performance of two learning rules. According to selfish learning, a player switches to the alternative strategy if and only if it increases the player’s payoff. Within our framework, selfish learning can be represented by the objective function $V_{S}\left(\mathbf {p},\mathbf {q}\right) =\pi _{X}\left(\mathbf {p},\mathbf {q}\right)$, which the player strives to maximize (Fig. 1e). We refer to such a player as a selfish learner. The other learning rule is *FMTL* (Fig. 1f). *FMTL* has two objective functions, $\mathcal {V}=\left\lbrace V_{E},V_{F}\right\rbrace$. The first objective is to achieve efficiency. With the objective function $V_{E}\left(\mathbf {p},\mathbf {q}\right) =\pi _{X}\left(\mathbf {p},\mathbf {q}\right) +\pi _{Y}\left(\mathbf {p},\mathbf {q}\right)$, the player aims to maximize the group’s total payoff. The other objective is fairness. Here, a player aims to minimize payoff differences, which is equivalent to maximizing the objective $V_{F}\left(\mathbf {p},\mathbf {q}\right) =-\left|\pi _{X}\left(\mathbf {p},\mathbf {q}\right) -\pi _{Y}\left(\mathbf {p},\mathbf {q}\right)\right|$. *FMTL* prioritizes efficiency if the players’ current payoffs are sufficiently close, and it prioritizes fairness if there is substantial inequality. For a precise description of the priority assignment, see “Methods.”

### Learning dynamics across different repeated games

To compare the two learning rules, we first assume each player’s learning rule is fixed. Across a range of different two-player games, we explore how the players’ learning rules affect their payoffs. As the baseline scenario, we consider two selfish learners. We then contrast this scenario with groups where either one or both players use *FMTL*.

#### Prisoner’s dilemma

**Fig. 2.**
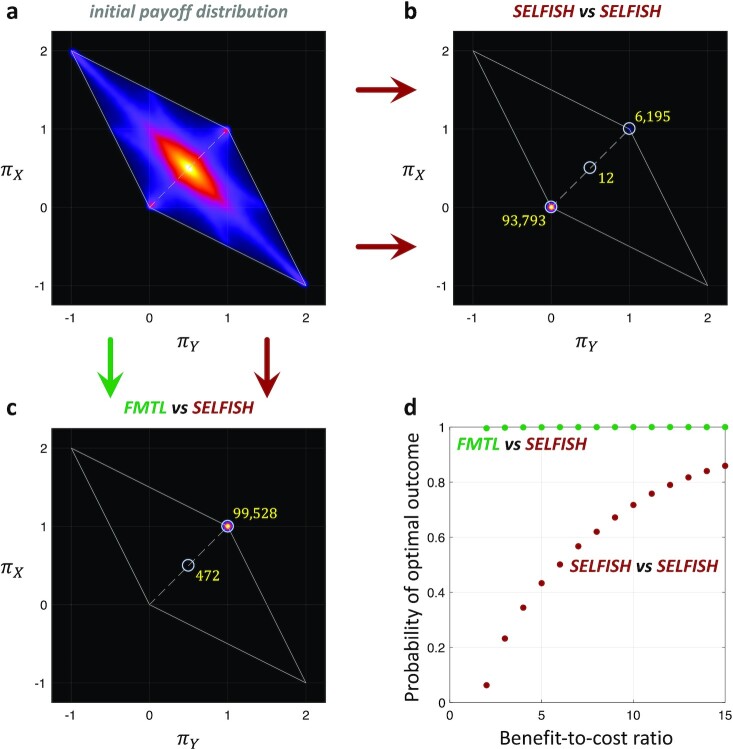
*FMTL* versus a selfish learner in the repeated donation game. (a) *X* and *Y* initially choose random strategies and receive payoffs that fall within the feasible region. (b) When *X* and *Y* are both selfish learners, we let *X* and *Y* update until neither one has accepted a new strategy for 10^4^ update steps. This process is repeated for 10^5^ iterations. The resulting distribution of payoffs is concentrated around the mutual defection payoff. Only a small number of runs (approximately 6%) result in mutual cooperation, and an even smaller number settle at alternating cooperation. (c) When *X* plays *FMTL* instead, final payoffs are concentrated around the payoff for mutual cooperation. This is also the fair and socially optimal outcome. (d) As one may expect, two selfish learners require a substantial benefit-to-cost ratio to coordinate on the optimal outcome (*b* − *c*) with high probability. In contrast, *FMTL* against a selfish learner gives excellent outcomes even when *b*/*c* is small. The endpoints in (b) and (c) are based on 10^5^ random initial strategy pairs, and for *b* = 2 and *c* = 1.

We start by exploring how players fare in one of the most basic and well-studied social dilemmas, the donation game (13). Here, cooperation means paying a cost *c* > 0 to deliver a benefit of *b* > *c* to the coplayer. This results in a prisoner’s dilemma: players individually prefer to defect, yet mutual cooperation yields a better payoff than mutual defection. Players start out with random memory-one strategies (Fig. 2a) and the game is repeated for many rounds (see “Methods”). Previous work shows there are four types of symmetric equilibria among memory-one players (54): players either both cooperate, both defect, alternate, or they use so-called equalizers (20,69).

We use simulations to explore which of these equilibrium outcomes is eventually realized (if any), depending on which learning rules the players apply. When two selfish learners interact, most of the time they either end up in mutual cooperation or mutual defection (Fig. 2b). Although cooperation is socially optimal, an overwhelming majority of simulations give rise to an all-defection equilibrium with low payoffs. The observed learning dynamics change completely if one (or both) of the two learners switches to *FMTL*. In that case, most simulated pairs of learners end up cooperating (Fig. 2c; see Figure S1 for a depiction of the final strategies). Remarkably, players yield almost full cooperation already for low benefit-to-cost ratios, for which cooperation is usually difficult to establish (21) (Fig. 2d).

**Fig. 3.**
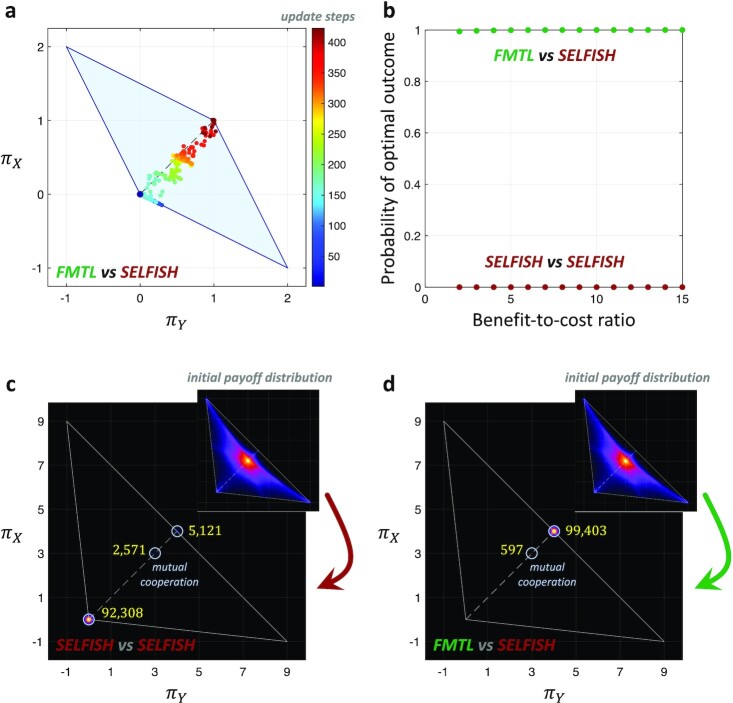
Robustness of *FMTL*. (a) To illustrate how *FMTL* can help players to escape from mutual defection, we consider a scenario where *X* uses *FMTL* and *Y* is a selfish learner. Initially, both *X* and *Y* play ALLD (“always defect”). Even under such hostile initial conditions, the process moves toward mutual cooperation. (b) Across all benefit-to cost-ratios, pairs with an *FMTL* learner reliably escape mutual defection. In contrast, two selfish learners never escape. (c) and (d) In addition to the donation game, we also consider simulations for a prisoner’s dilemma with (*S* + *T*)/2 > *R*. In this game, optimal play requires alternating cooperation and defection. When *FMTL* is matched with a selfish learner, players are indeed most likely to settle at this outcome, although they have no explicit information about *R* relative to (*S* + *T*)/2. Game parameters: (a and b) *R* = 1, *S* = −1,  *T* = 2,  and *P* = 0; (c and d) *R* = 3, *S* = −1, *T* = 9, and *P* = 0.

To explore which mechanism allows *FMTL* to evade inefficient equilibria against selfish learners, we consider simulations in which initially both players defect (Fig. 3a). In this initial state, the players’ payoffs are equal but inefficient. As a result, *FMTL* prioritizes efficiency over fairness, leading the respective learner to cooperate occasionally. While this reduces the payoff to *FMTL*, it also provides some strategic leverage. By cooperating conditionally, the learner can affect how profitable it is for the selfish opponent to cooperate. Once the *FMTL* player adopts a strategy that makes cooperation a best response, even a selfish opponent has an incentive to adapt (Figure S2 and Video S1). In this way, *FMTL* triggers dynamics of ever-increasing cooperation rates. Eventually, players reach an equilibrium in which both players cooperate. Different simulated trajectories vary due to stochasticity, but they almost always lead from defection to cooperation, independent of the magnitude of the benefit-to-cost ratio (Fig. 3b). We observe similar dynamics when players start out with random strategies (see Figure S3).

Donation games may favor successful learning because efficiency requires only a simple pattern of behavior: both players merely need to cooperate each round. Instead, additional coordination problems might arise if efficiency requires the players to cooperate in turns. This problem occurs, for example, in a prisoner’s dilemma with *R* < (*S* + *T*)/2. Here, it is socially optimal to agree on a policy of alternation: *X* cooperates and *Y* defects in even rounds, whereas *X* defects and *Y* cooperates in odd rounds. Again, we explore the dynamics of this game when players use either selfish learning or *FMTL*. When they both use selfish learning, they typically fail to cooperate altogether (Fig. 3c). But if one of them adopts *FMTL*, they overwhelmingly discover the optimal policy of alternation (Fig. 3d). We observe a similar pattern in the context of a harmony game (here, *R* > *T* and *S* > *P*, such that cooperation is dominant (70)). When *R* < (*S* + *T*)/2, two selfish learners typically coordinate on mutual cooperation. But if one of the two players switches to *FMTL*, they both achieve the superior alternation outcome. Specifically, when two selfish learners interact in a game with payoffs *R* = 1, *S* = 2, *T* = 0.5, and *P* = 0, we find that over 99% of runs give a payoff of (*R, R*) = (1, 1). Once one of the learners switches to *FMTL*, more than 99% of runs end up at ((*S* + *T*)/2, (*S* + *T*)/2) = (1.25, 1.25).

#### Generalized social dilemmas: stag hunt and snowdrift games

While the prisoner’s dilemma has been instrumental in modeling human cooperation, there are other natural rankings of the game’s payoffs. These alternative rankings result in weaker forms of conflict. To further explore the performance of *FMTL*, we consider generalized social dilemmas, defined by three properties (49–51): (i) the payoff for mutual cooperation exceeds the payoff for mutual defection, *R* > *P*; (ii) when players choose different actions, the defector obtains a larger payoff than the cooperator, *T* > *S*; and (iii) irrespective of their own action, players prefer their opponent to cooperate, *R* > *S* and *T* > *P*. In addition to the prisoner’s dilemma and the harmony game, there are two more generalized social dilemmas (71): the stag hunt (72) and the snowdrift (73,74) game.

The stag hunt game has the payoff ranking *R* > *T* > *P* > *S*. In particular, mutual defection is an equilibrium of the one-shot game, but so is mutual cooperation. For repeated stag hunt games, we find that players do not need to settle at socially optimal outcomes, even if they both adopt *FMTL* (Figure S4a–f). Such inefficiencies may arise more generally. They can occur in all games with equilibria that are fair but inefficient, and where one player alone is unable to raise the group payoff (see Supporting Information for analytical results). Nevertheless, we find that *FMTL* makes players less likely to settle at such inefficient equilibria in the first place. As a result, each player performs better on average if at least one of them adopts *FMTL*.

In the other generalized social dilemma, the snowdrift game, the payoff ranking is *T* > *R* > *S* > *P*. Mutual defection is no longer an equilibrium because unilateral cooperation is a better outcome for *both* players. When two selfish learners engage in a repeated snowdrift game, they often approach one of these two pure equilibria. Eventually, one player cooperates each round and the other defects (Figures S4g and S5a). Which of the two players ends up cooperating depends on their initial strategies and on chance. In contrast, if one of them switches to *FMTL*, players most likely coordinate on a pattern of play that is both fair and efficient. Similar to the prisoner’s dilemma, this pattern requires players to either mutually cooperate (if (*S* + *T*)/2 < *R*; Figure S4h), or to cooperate in an alternating fashion (if (*S* + *T*)/2 > *R*; Figure S5b). In the latter case, already two selfish learners tend to achieve an efficient (albeit possibly unfair) outcome. The role of *FMTL* here, relative to selfish learning, is to eliminate inequality.

#### Alternative forms of conflict: hero game

Finally, we consider an example that does not meet the conditions of a social dilemma: the hero game (15). This game, sometimes referred to as a (symmetric) battle of the sexes (75,76), satisfies *S* > *T* > *R* ≥ *P*. Here, mutual *C* is preferred to mutual *D*, but when both players use *C*, a single player can act as a “hero” and improve both players’ payoffs by switching to *D* (77). The one-shot game has two pure equilibria, but players disagree on which equilibrium they prefer. When two selfish learners engage in the repeated game, they frequently converge toward one of these pure one-shot equilibria (Figure S5d). In contrast, groups with at least one *FMTL* player reliably learn to alternate (Figure S5e and f). Selfish learning is able to generate efficient outcomes, but only *FMTL* makes sure the realized outcome is fair, irrespective of the learning rule of the opponent. Table S1 summarizes these results across the different games we study.

### Evolutionary dynamics of learning rules

After analyzing how different learning rules affect adaptation, we study how the learning rules themselves evolve over time. We consider two timescales. In the short run, the players’ learning rules are fixed. Players use their learning rule to adapt to their opponent. In the long run, learning rules reproduce, based on how well players with the respective rule perform. This process may reflect cultural or biological evolution (i.e. successful learners are either imitated more often, or they have more offspring). Just as repeated games can be thought of as a “supergame” layered over a one-shot game (53) (Fig. 1a and b), the process describing the evolution of learning rules can be thought of as a supergame layered over the repeated game (Fig. 1c–f).

#### Description of the evolutionary process

To describe this supergame formally, we consider a population of learners who use either selfish learning (S) or *FMTL* (F). In the short run, players are randomly matched to engage in repeated games with a fixed partner. Over the course of their interactions, they update their strategies according to their learning rules, as in the previous section. As a result, they receive a payoff that depends on the game being considered, the players’ learning rules, and on the time that has passed for learning to unfold. For a given game, let *a_ij_*(*n*) denote the expected payoff of a learner *i* against another learner *j* after *n* learning steps (i.e. after the players had *n* opportunities to revise their strategies). We estimate these payoffs using numerical simulations.

For a given number of learning steps *n*, the four payoffs *a*_SS_(*n*), *a*_SF_(*n*), *a*_FS_(*n*), and *a*_FF_(*n*) can be assembled in a 2 × 2 payoff matrix. We interpret the entries of this matrix as the payoff of each learning rule, and we interpret *n* as the players’ learning horizon. Payoff matrices for different values of *n* reflect different assumptions on how patient players are. For small *n*, players are impatient. They assess the quality of their learning rule by how well they perform after only a few learning steps. In contrast, for large *n*, players assess the quality of their learning rule according to how well it performs eventually (even if it may be ineffective in the short run).

For a given payoff matrix, we use the replicator equation to model the evolutionary dynamics among learning rules (78). The equation tracks the frequencies of each learning rule over time, and it favors those rules with higher average payoffs (see “Methods”). For 2 × 2 games, as in our case, replicator dynamics can result in four different scenarios (10, 13). Either one learning rule is globally stable (dominance), there is a mixed population that is globally stable (coexistence), each learning rule is locally stable (bistability), or any mixed population is stable (neutrality).

#### Evolutionary dynamics across different games

**Fig. 4.**
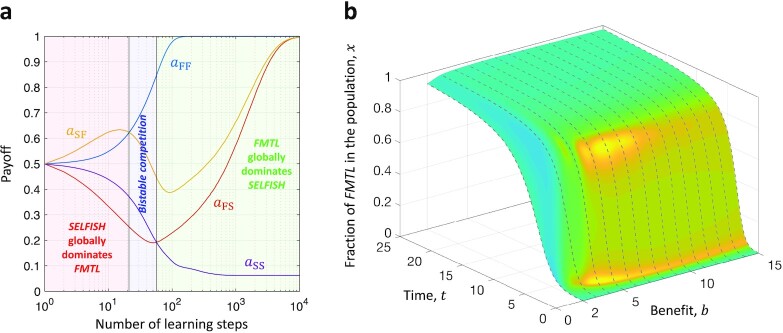
Evolutionary dynamics of *FMTL* in the donation game. (a) In general, the players’ payoffs depend on their learning rule and on how many learning steps they had to update their strategies. Here, we show these (supergame) payoffs for the donation game with *b* = 2 and *c* = 1. If the number of learning steps is small (fewer than ≈ 20 in this example), the payoffs of *FMTL* and selfish learning take the form of a prisoner’s dilemma: *FMTL* yields the better payoff when adopted by everyone, but it is dominated by selfish learning. With slightly more learning steps, the dynamics transition into bistable competition. Moreover, after only ≈ 60 learning steps, *FMTL* globally dominates selfish learning. (b) Starting from an initial frequency of *x*_0_ = 10^−3^, *FMTL* quickly spreads in a population of selfish learners under replicator dynamics. Depicted here are evolutionary trajectories for the donation game when *c* = 1 and *b* varies from 2 to 15. The dynamics in (b) are shown for the supergame payoffs after convergence of the learning process; but due to (a) similar results hold when the timescale of learning is much shorter.

The resulting dynamics between the two learning rules depend on the game and on the players’ learning horizon. Figure   4(a) shows the possible scenarios for the donation game. If the learning horizon is short, then learning rules are selected according to whether they result in an immediate advantage. As a result, we find that selfish learning is dominant, as one might expect. However, as players become increasingly patient, the dynamics first take the form of bistable competition, and then *FMTL* becomes globally stable. In this parameter regime, which starts after *n* ≈ 60 learning steps, already a small initial fraction of *FMTL* learners is sufficient to drive selfish learning to extinction (Fig. 4b). Since clusters of *FMTL* learners perform better than clusters of selfish learners, the evolutionary advantage of *FMTL* is even more pronounced in structured populations (79, 80) (see “Methods” for details).

These patterns generalize to other games. In each case, selfish learning is dominant when players have a very short learning horizon. As players become sufficiently patient, *FMTL* becomes dominant in all variants of the prisoner’s dilemma and the stag-hunt game, as well as in the snowdrift game when (*S* + *T*)/2 < *R* (Figures S6 and S7). Only for the snowdrift game with (*S* + *T*)/2 > *R* and the hero game may selfish learning prevail for long learning horizons. Table S2 gives a summary of these results. Interestingly, the games in which *FMTL* becomes globally stable are exactly those in which two selfish learners are at a considerable risk of settling at inefficient equilibria (Table S1). Conversely, the games in which selfish learning can prevail are those in which selfish learners tend to achieve efficient outcomes (Figure S5a and d). For example, in all simulated cases of the hero game, selfish learners settle at equilibria with maximum average payoffs (even though payoffs may be shared unfairly). Because replicator dynamics depend on only the average payoffs (not on the distribution of payoffs), and because selfish learning achieves the maximum average payoff against itself, *FMTL* cannot invade. This conclusion does not require replicator dynamics. Instead, it remains true for all evolutionary dynamics that depend on only a trait’s average payoff, including stochastic models of weak selection (81).

Importantly, for *FMTL* to be successful, each of its components, fairness and efficiency, is vital. To illustrate this point, we repeat the previous simulations for the donation game with alternative learning rules (Figure S9): players either value only fairness, only efficiency, or they combine fairness with selfishness. Against a selfish learner, we find that each alternative rule performs worse than *FMTL*. When the focal player values only fairness, payoffs tend to be equal but inefficient (Figure S9a). When the focal player values only efficiency, the focal player tends to cooperate unconditionally, whereas the selfish coplayer defects (Figure S9b). Finally, when the focal player combines fairness and selfish learning, the overall performance is similar to the case of two selfish learners (Figure S9c). These results highlight that *FMTL*’s two components are effective only when combined. Learners who only aim for efficiency are subject to exploitation; learners who only value fairness obtain equal payoffs, but at the price of obtaining low payoffs.

### Selfishness versus fairness in a repeated prisoner’s dilemma experiment

The results presented herein cast doubt on the assumption that selfish learning can fully explain human adaptation processes in repeated games. While it has been suggested that selfish learning reasonably approximates cooperative behavior in linear multiplayer games (82,83), such games differ in crucial aspects from the two-player interactions studied herein. For example, in multiplayer games, each individual has less of an impact on other group members. Moreover, each player’s defection can only be punished collectively (by withholding cooperation from *all* other group members). In contrast, pairwise interactions provide individuals with more immediate possibilities to affect their coplayer’s future behavior. It is pairwise games where learning rules have the strongest strategic impact.

**Fig. 5.**
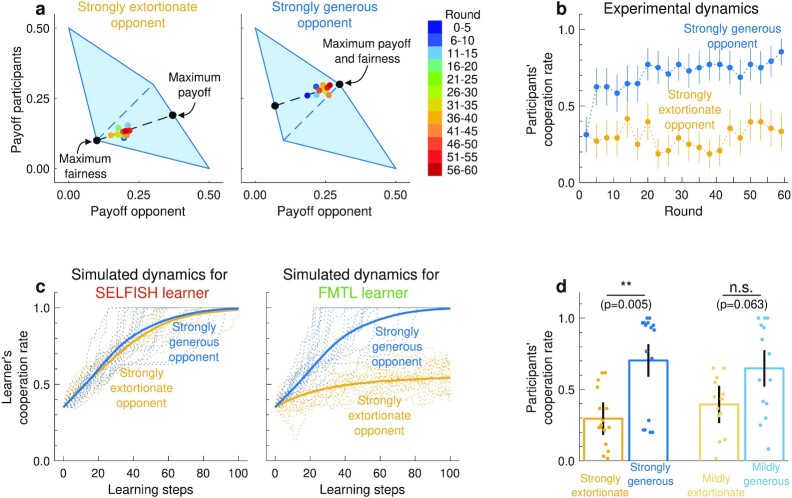
Selfish learning fails to explain human behavior when there is a tension between payoff maximization and fairness. To empirically distinguish between selfish learning and fairness-mediated learning, we reanalyze data from a repeated prisoner’s dilemma experiment (84). In this experiment, human participants interact with a computerized opponent who implements a fixed strategy. Human participants are not informed about the nature of their opponent. (a) The computer implements either an extortionate (20) or a generous (25) zero-determinant strategy. Such strategies ensure that there is a linear relationship between the payoffs of the two players (indicated by the black dashed line). If human participants wish to maximize their payoffs, they should learn to cooperate in either case. If participants wish to enhance fairness, they should cooperate against generous strategies but not against extortioners. (b) The experiment finds that humans become more cooperative against generous strategies. There is no trend toward cooperation against extortioners. (c) To shed further light on these observations, we simulate the possible learning dynamics. We consider a single learner, who either adopts selfish learning or *FMTL*. Only for *FMTL* do we recover that subjects fail to fully cooperate against extortioners. (d) The previous results are based on two treatments in which the trade-off between fairness and payoff maximization is strongest. When the computer instead implements so-called mildly generous and extortionate strategies, this trade-off is weaker. As a result, while generous coplayers still tend to induce more cooperation, the effect is now smaller. Error bars indicate standard errors, and any statistical statements are based on nonparametric tests (Mann–Whitney U test and Wilcoxon test). For details, see “Methods.”

To explore the relevance of selfish learning for pairwise games, we reanalyze data from a prisoner’s dilemma experiment (84). In this experiment, human participants play against a computer program (unbeknownst to the human subjects). The computer program either implements an extortionate (20) or a generous (25) “zero-determinant” strategy (20,21). Against both classes of strategies, the payoff-maximizing choice for human participants is to cooperate in every round. However, while full cooperation is also the fairness-maximizing choice against generous opponents, it leads to maximally unequal outcomes against extortioners (Fig. 5a).

There are two reasons why this experimental paradigm allows for a clean comparison between selfish learning and other learning rules based on other-regarding preference: (i) Because the computer’s strategy is fixed (and known to the researcher), each human participant’s learning behavior can be studied in isolation. (ii) The two competing learning rules make opposing predictions for this experiment: If human participants use selfish learning, they should equally learn to cooperate against either computer strategy. In contrast, if behavior is better described by *FMTL*, we expect more cooperation against the generous strategy (Fig. 5c; see “Methods” for a detailed description of the experimental setup and our predictions).

In Fig. 5(b), we compare experimental data for the “strongly extortionate” and the “strongly generous” computer strategy (for which fairness considerations are most likely to impede cooperation in the extortion treatment). For both treatments, human cooperation rates are similar in the beginning (31.3% during the first three rounds). Against the generous program, humans increase their cooperation rate to 85.4% by the end of the experiment (during the last three rounds). In contrast, against the extortionate program, overall cooperation rates are largely unchanged (33.3%), although the monetary incentives for cooperation are identical. These experimental results are consistent with learning behavior that is shaped by fairness considerations. In line with this view, when the computer implements a strategy that is only mildly extortionate or generous, there is less of a difference in human cooperation rates (Fig. 5d). Moreover, the difference disappears altogether if individuals are informed ahead of the experiment that they are matched with a computerized opponent, in which case fairness considerations can be expected to be absent (85). Overall, these results suggest that fairness motives are of crucial importance to describe human behavior in pairwise interactions, even though they may be less salient in multiplayer games (82, 83).

## Discussion

Given the extensive effort to explore strategies that sustain cooperation in repeated social dilemmas (18–30), it seems quite remarkable that relatively little is known about how strategies with desirable properties can be learned most effectively. Instead, much of the existing work tends to take the way in which individuals learn as given. While details vary between studies (40–47), most often it is assumed that individuals adopt strategies that enhance their own payoff, abandoning strategies that are personally disadvantageous. This modeling assumption could be justified on theoretical grounds if selfish payoff maximization were indeed an optimal learning policy. Here, we have explored under which conditions selfish learning can be expected to succeed. We show theoretically that selfish learning performs well when individuals wish to optimize their short-run performance. However, if individuals are motivated by how well they fare eventually, selfish learning can be of limited use in navigating conflicts of interest.

To assess the performance of selfish learning, we contrast it with an alternative rule termed*FMTL*. Rather than maximizing just one’s own payoff, a learner who adopts *FMTL* strives to enhance either the efficiency of the resulting game outcome or its fairness. By striving to increase efficiency, *FMTL* attempts to evade inferior equilibria that leave all group members worse off. By striving to increase fairness, *FMTL* avoids exploitation when other group members continue to learn selfishly. Using individual-based simulations, we show that *FMTL* can help individuals to settle at better equilibria. These equilibria are either more equitable or more efficient. Moreover, when players select their learning rules according to how well they perform eventually, *FMTL* outcompetes selfish learning for most of the games we study. Qualitatively, the ability of *FMTL* to draw out better outcomes also extends to learning based on imitation as opposed to introspection (Section S4 of Supporting Information; Figures S11 and S12) as well as asymmetric games (Section S5 of Supporting Information; Figure S13).

Our theoretical results are further corroborated by an analysis of human cooperation in a repeated prisoner’s dilemma with fixed opponent strategies (84,85). When interactions entail a trade-off between fairness and payoff maximization, selfish learning fails to explain crucial patterns of human behavior (Fig. 5). There is a considerable literature within behavioral economics seeking to describe human behavior that deviates from models of pure self-interest (86). In particular, there is ample empirical evidence that fairness and efficiency are important drivers of human behavior. Humans value fairness starting from a young age (87, 88), and they are often willing to accept substantial reductions in their own income to achieve more egalitarian outcomes (89). Such a demand for fairness can have substantial economic consequences, as it constrains a firm’s profit seeking behavior (90) and market prices (91). There is similar experimental evidence about the importance of efficiency (6, 92). In dictator games, human participants often give up some of their own payoff in order to increase that of the pair (86,93). At the same time, however, human decisions to increase efficiency via “gifts” are constrained by fairness considerations (94), consistent with the constraint built-in to *FMTL*.

Our results are related to the “indirect evolutionary approach,” which explores the evolution of preferences with game-theoretic methods (95–98). The respective literature distinguishes between objective and subjective payoffs. Objective payoffs include monetary rewards and reproductive success (fitness). Subjective payoffs capture how individuals experience certain outcomes and what they strive to maximize when making decisions. Similar to our model, this approach is “indirect” because preferences that guide behaviors do not need to align with objective payoffs. However, while this literature explicitly models the evolutionary dynamics of preferences (98, 99), it usually does not describe how preferences affect the way individuals learn (see Supporting Information for a more detailed discussion). The learning process is the main focus of our study. We explore how different learning heuristics influence the way in which subjects navigate between equilibria of differing efficiency. To this end, our framework requires a notion of learning rules that is slightly more general than the notion of preferences considered before. Learning rules do not only specify the objectives that players wish to maximize. Instead, they also determine how players choose between different objectives and how they generate alternative strategies. In this way, our framework also applies to learning on shorter time scales, wherein subjects update their learning rules even before the learning dynamics reach an equilibrium (Table S2).

Our results also have implications for objective design in multiagent learning. Previous work has shown that objectives based on a convex combination of the players’ payoffs can improve outcomes relative to selfish learning in stag hunt games (100). An alternative approach is to implement a look-ahead into the opponent’s learning process in order to shape their future behavior (52) (however, this forward-looking approach requires substantial information about the opponent; see Supporting Information). While the space of possible objective functions is vast, we introduce a learning rule (one of many, perhaps) that can outcompete naïve selfish learning. Compared to the rules considered previously, *FMTL* has the advantage of being comparably simple, and its components are natural for both humans and machines to implement. Much in the same way that the most rudimentary strategy (tit-for-tat) won Axelrod’s tournaments (55), here too a simple learning rule is able to align incentives with a selfish learner.

Even if individuals are ultimately driven by their own advantage, optimal learning rules may require them to take into account other considerations, such as the well-being of others. Of course, the repeated two-player games studied herein cannot capture all realistic interactions in which learning is relevant. However, these simple baseline models can help to understand the general principles at work in more complex settings (101). Delineating which aspects are crucial for successful learning is, in our view, one of the most exciting directions for future research.

## Methods

### Strategies and payoffs in repeated games

All of the strategies we consider for repeated games are “memory-one” strategies, which means that they consist of a five-tuple of probabilities, (*p*_0_, *p_CC_*, *p_CD_*, *p_DC_*, and *p_DD_*), where *p*_0_ is the probability of cooperating (action *C*) in the initial round and *p_xy_* is the probability a player cooperates after using *x* ∈ {*C, D*} in the previous round against an opponent using *y* ∈ {*C, D*}. The initial distribution over the outcomes (*CC, CD, DC*, and *DD*), where the first action is that of *X* and the second is that of *Y*, is

(1) $$\begin{eqnarray*}
\nu _{0}\left(\mathbf {p},\mathbf {q}\right) &= \left(p_{0}q_{0},p_{0}\left(1-q_{0}\right) ,\left(1-p_{0}\right) q_{0},\left(1-p_{0}\right)\left(1-q_{0}\right)\right) .
\end{eqnarray*}
$$

Following the initial round, transitions between states are described by the stochastic matrix

(2) $$\begin{eqnarray*}
M\left(\mathbf {p},\mathbf {q}\right) = \left ({\begin{array}{cccc}
p_{CC}q_{CC} & p_{CC}\left(1-q_{CC}\right) & \left(1-p_{CC}\right) q_{CC} & \left(1-p_{CC}\right)\left(1-q_{CC}\right) \\
p_{CD}q_{DC} & p_{CD}\left(1-q_{DC}\right) & \left(1-p_{CD}\right) q_{DC} & \left(1-p_{CD}\right)\left(1-q_{DC}\right) \\
p_{DC}q_{CD} & p_{DC}\left(1-q_{CD}\right) & \left(1-p_{DC}\right) q_{CD} & \left(1-p_{DC}\right)\left(1-q_{CD}\right) \\
p_{DD}q_{DD} & p_{DD}\left(1-q_{DD}\right) & \left(1-p_{DD}\right) q_{DD} & \left(1-p_{DD}\right)\left(1-q_{DD}\right) \end{array}} \right ). \!\!\!\!\! \nonumber \\
\end{eqnarray*}
$$

After *t* ≥ 0 rounds, the distribution over states is $\nu _{t}\left(\mathbf {p},\mathbf {q}\right) =\nu _{0}\left(\mathbf {p},\mathbf {q}\right) M\left(\mathbf {p},\mathbf {q}\right)^{t}$. With discounting factor λ ∈ [0, 1), the mean payoffs to *X* and *Y* when *X* plays $\mathbf {p}$ and *Y* plays $\mathbf {q}$ are

(3a) $$\begin{eqnarray*}
\pi _{X}\left(\mathbf {p},\mathbf {q}\right) &=& \left(1-\lambda \right)\sum _{t=0}^{\infty }\lambda ^{t} \lt \nu _{t}\left(\mathbf {p},\mathbf {q}\right) , \left(R,S,T,P\right) \gt \nonumber \\
&=& \lt \left(1-\lambda \right) \nu _{0}\left(\mathbf {p},\mathbf {q}\right)\left(I-\lambda M\left(\mathbf {p},\mathbf {q}\right)\right)^{-1} , \left(R,S,T,P\right)\gt ;
\end{eqnarray*}
$$

(3b) $$\begin{eqnarray*}
\pi _{Y}\left(\mathbf {p},\mathbf {q}\right) &=& \left(1-\lambda \right)\sum _{t=0}^{\infty }\lambda ^{t} \lt \nu _{t}\left(\mathbf {p},\mathbf {q}\right) , \left(R,T,S,P\right) \gt \nonumber \\
&=& \lt \left(1-\lambda \right) \nu _{0}\left(\mathbf {p},\mathbf {q}\right)\left(I-\lambda M\left(\mathbf {p},\mathbf {q}\right)\right)^{-1} , \left(R,T,S,P\right)\gt ,
\end{eqnarray*}
$$

respectively, where <·, · > denotes the standard inner (dot) product on $\mathbb {R}^{4}$. To approximate the results of an infinite-horizon game, we use a discounted game with a small discounting rate, λ = 1 − 10^−3^. In this way, we ensure that the limiting payoffs always exist, even if the players adopt strategies that allow for multiple absorbing states, such as tit-for-tat (13).

### Learning rules

In the following, we describe the four components of a learning rule (the initial strategy distribution, the sampling procedure, the set of objective functions, and the priority assignment) for both selfish learning and *FMTL*.

#### Distribution over initial strategies

When two individuals are first paired with one another, they each choose an initial strategy for their first interaction, which is to be subsequently revised during the learning process. Two of the most natural choices are (i) to choose each coordinate of the strategy independently from a uniform distribution on [0, 1], and (ii) to choose each coordinate independently from an arcsine (Beta(1/2, 1/2)) distribution on [0, 1]. For the figures presented herein, we use the latter distribution because it is more effective in exploring the corners of the space [0, 1] ^5^ of memory-one strategies (36). However, we obtain similar qualitative results for a uniform initial distribution. In addition, we also explore the learning dynamics that arise when players initially defect unconditionally (Fig.   3a and b).

#### Sampling procedure

We assume strategy sampling to be local in the following sense (see also Fig. 1c). Let *s* ∈ [0, 1] and suppose that *z_i_* is uniformly distributed on [ − *s, s*] (with *z_i_* independent of *z_j_* for *j* ≠ *i*). If *p_i_* is the coordinate being “mutated” at a given time step, then the candidate sample of this coordinate in the next step is $p_{i}^{\prime }=\min \left\lbrace \max \left\lbrace p_{i} + z_{i} , 0 \right\rbrace , 1 \right\rbrace$. We use *s* = 0.1 in our examples, which allows for exploration while ensuring that the candidate strategy is not too distant from the current strategy (so that desirable properties of the current strategy are not immediately discarded). Relatively small values of *s* make the trajectories of the learning process more interpretable (e.g. Fig. 3a), but they also slow down the learning process. While taking a different value of *s* can change the overall performance of each learning rule, we did not find a scenario in which it reverses the relative ranking of selfish learning compared to *FMTL*.

#### Objective functions

Once a candidate strategy is sampled, the respective player decides whether to accept it based on the player’s objectives. To this end, each learning rule specifies a set of objective functions,

(4) $$\begin{eqnarray*}
\mathcal {V} &= \Big \lbrace V\ \mid \ V:\left[0,1\right]^{5}\times \left[0,1\right]^{5} \rightarrow \mathbb {R}\Big \rbrace .
\end{eqnarray*}
$$

Each objective function *V* takes the players’ memory-one strategies $\mathbf {p}$ and $\mathbf {q}$ as an input, and returns a value that indicates to which extent the players’ objectives are met. Throughout our study, we consider objective functions that depend on only the players’ payoffs, $\pi _{X}\left(\mathbf {p},\mathbf {q}\right)$ and $\pi _{Y}\left(\mathbf {p},\mathbf {q}\right)$, but more general formulations are possible . A candidate strategy $\mathbf {p^{\prime }}$ is accepted if $V\left(\mathbf {p^{\prime }},\mathbf {q}\right) \gt V\left(\mathbf {p},\mathbf {q}\right)$, and it is discarded otherwise.

For the simulations, we assume the candidate strategy is accepted if and only if $V\left(\mathbf {p^{\prime }},\mathbf {q}\right) \gt V\left(\mathbf {p},\mathbf {q}\right) +\varepsilon$, where 0 < ε ≪ 1 is a small threshold. This assumption prevents floating point errors from resulting in faulty decisions, particularly when $V\left(\mathbf {p},\mathbf {q}\right)$ is extremely close to $V\left(\mathbf {p^{\prime }},\mathbf {q}\right)$. While any one such faulty decision might have negligible effects on the learning process, these mistakes can accumulate over many time steps. In all of our numerical examples, the threshold we use is ε = 10^−12^. The use of such a threshold can also be interpreted in terms of bounded rationality (102). Due to limitations on cognition or information, the learner may not be able to distinguish two values that are sufficiently close together. Similarly, in the presence of noise perturbing the observed payoffs, ε may be thought of parametrizing the confidence an agent has that there will truly be an improvement in *V* by switching to the new strategy.

We note that objective functions take both players’ strategies as an input. One interpretation of this assumption is that the learner needs to have precise knowledge of the coplayer’s strategy in order to forecast whether a given strategy change is profitable. However, we note that this rather stringent assumption is in fact not necessary. Instead, we only need to assume that individuals can reliably assess the sign of $V\left(\mathbf {p}^{\prime },\mathbf {q}\right) -V\left(\mathbf {p},\mathbf {q}\right) -\varepsilon$. That is, players only need to be able to make qualitative assessments.

For selfish learners, the set of objective functions contains a single element, $\mathcal {V}=\left\lbrace V_{S}\right\rbrace$ with $V_{S}\left(\mathbf {p},\mathbf {q}\right) =\pi _{X}\left(\mathbf {p},\mathbf {q}\right)$. For *FMTL*, the set of objective functions is $\mathcal {V}=\left\lbrace V_{E},V_{F}\right\rbrace$. Here, $V_{E}\left(\mathbf {p},\mathbf {q}\right) = \pi _{X}\left(\mathbf {p},\mathbf {q}\right) + \pi _{Y}\left(\mathbf {p},\mathbf {q}\right)$ reflects the objective to increase efficiency, whereas $V_{F}\left(\mathbf {p},\mathbf {q}\right) = -\left|\pi _{X}\left(\mathbf {p},\mathbf {q}\right) -\pi _{Y}\left(\mathbf {p},\mathbf {q}\right)\right|$ corresponds to the objective of enhancing fairness.

#### Priority assignments

A learning rule’s priority assignment Ω determines which of the players’ different objectives is currently maximized. Formally, a priority assignment is a map $\Omega :\left[0,1\right]^{5}\times \left[0,1\right]^{5}\rightarrow \Delta \left(\mathcal {V}\right)$, where $\Delta \left(\mathcal {V}\right)$ denotes the space of probability distributions on $\mathcal {V}$. For each of the players’ current memory-one strategies $\mathbf {p},\mathbf {q}\in \left[0,1\right]^{5}$ it determines the probability with which each possible objective $V\in \mathcal {V}$ is chosen for maximization.

In the case of selfish learning, the priority assignment is trivial, because there is only one possible objective to choose from. In the case of *FMTL*, the priority assignment takes the form $\Omega \left(\mathbf {p},\mathbf {q}\right)=\left(\omega ,1-\omega \right)$, where $\omega =\omega \left(\mathbf {p},\mathbf {q}\right)$ is the weight assigned to efficiency. In our formulation of *FMTL*, ω depends on the current magnitude of the payoff difference (see Figure S10a),

(5) $$\begin{eqnarray*}
\omega \left(\mathbf {p},\mathbf {q}\right) &= \exp \left( -\frac{\left(\pi _{X}\left(\mathbf {p},\mathbf {q}\right) -\pi _{Y}\left(\mathbf {p},\mathbf {q}\right)\right)^{2}}{2\sigma ^{2}} \right).
\end{eqnarray*}
$$

The parameter σ > 0 reflects a player’s tolerance with respect to inequality. In the limit σ → 0, a player always aims to enhance fairness, whereas in the opposite limit σ → ∞, the player always aims to improve efficiency. In general, neither efficiency nor fairness alone are sufficient for establishing good outcomes against selfish learners (Figure S9). Finding a proper balance between these two objectives depends on the nature of the interaction, and as a result the optimal value of σ can vary from game to game.

We choose σ by taking the average payoff for *FMTL* versus a selfish learner over a small number of runs (10^3^), for each σ ∈ {0.01, 0.02, …, 1.00}. We then select σ based on which value maximizes the average payoff of the *FMTL* individual, except for when this value is comparable for all such σ, in which case we choose the value that minimizes the runtime. For the donation game (Figs 2c and d,3a and b, and 4; Figures S1, S3, S9c, and S13; Video S1), we use σ = 0.1; for the prisoner’s dilemma with (*S* + *T*)/2 > *R* (Fig. 3c and d; Figure S6b) and with (*S* + *T*)/2 < *P* (Figure S6a), we use σ = 1; for the stag hunt game with (*S* + *T*)/2 > *P* (Figures S4a–c and S7a), we use σ = 0.01; for the stag hunt game with (*S* + *T*)/2 < *P* (Figures S4d–f and S7b), we use σ = 1; for the snowdrift game with (*S* + *T*)/2 < *R* (Figures S4g–i and S7c), we use σ = 0.1; for the snowdrift game with (*S* + *T*)/2 > *R* (Figures S5a–c and S8a), we use σ = 1; and for the hero game (Figures S5d–f and S8b), we use σ = 0.1. The use of different values of σ in different games is a result of treating σ as a hyperparameter (103) that can be fine-tuned according to the nature of a given repeated game. However, we note that we get qualitatively similar (even if not completely optimized) results when we use a single value of σ across all our main examples (e.g. σ = 0.25).

Instead of a bell curve, ω could be a bump function such as

(6) $$\begin{eqnarray*}
\omega \left(\mathbf {p},\mathbf {q}\right) &= \left\lbrace \begin{array}{@{}l@{\quad }l@{}}\displaystyle e^{-\frac{\left(\pi _{X}\left(\mathbf {p},\mathbf {q}\right) -\pi _{Y}\left(\mathbf {p},\mathbf {q}\right)\right)^{2}}{\alpha ^{2}\sigma ^{2}\left(\alpha ^{2}-\left(\pi _{X}\left(\mathbf {p},\mathbf {q}\right) -\pi _{Y}\left(\mathbf {p},\mathbf {q}\right)\right)^{2}\right)}} & \left|\pi _{X}\left(\mathbf {p},\mathbf {q}\right) -\pi _{Y}\left(\mathbf {p},\mathbf {q}\right)\right| \lt \alpha \\
[.3cm] 0 & \left|\pi _{X}\left(\mathbf {p},\mathbf {q}\right) -\pi _{Y}\left(\mathbf {p},\mathbf {q}\right)\right|\geqslant \alpha \end{array}\right.
,
\end{eqnarray*}
$$

(see Figure S10b). In practice, we do not see significant qualitative differences between these two functions provided the parameters are chosen properly. However, relative to Eq. (6), the function in Eq. (5) does have the advantage of depending on only one shape parameter, σ.

#### Implementation of learning rules

For two players with given learning rules, we explore the resulting learning dynamics with simulations. The code is available (see “Data Availability” statement). For these simulations, the learning process is terminated after neither learner has accepted a new candidate strategy in a fixed threshold number of update steps (here, 10^4^). All of the examples we consider terminate.

In addition, Fig. 4(a) and Figures S6–S8 illustrate the expected payoffs as a function of the number of learning steps. In these graphs, the horizontal axis shows how many opportunities the two players had to revise their strategies. The vertical axis represents the average payoff over sufficiently many simulations. This payoff depends on the learning rules of the focal player and the opponent.

### Evolutionary dynamics of learning rules

The learning rules considered here may be viewed as strategies for a “supergame.” The payoffs of this supergame are given by the players’ expected payoffs after *n* updating steps. Here, we consider *n* as a fixed parameter of the model, and we refer to it as the player’s learning horizon. When *n* is small, individuals evaluate the performance of their learning rule based on how well they fare after a few learning steps. In contrast, when *n* is large, learning rules are evaluated according to how well they fare eventually.

Because we consider the competition between two learning rules, this supergame can be represented as a 2 × 2 matrix. To this end, we fix a base game G ∈ {HE, PD, SH, SD}, where HE is the hero game, PD is the prisoner’s dilemma, SH is the stag hunt game, and SD is the snowdrift game. For a given learning horizon *n*, we can write the resulting 2 × 2 matrix as

(7) $$\begin{eqnarray*}
\qquad {FMTL}\quad {SELFISH} \\
_{SELFISH}^{FMTL} \left ( _{a_{{\rm SF}}^{{\rm G}}\left(n\right)\quad a_{{\rm SS}}^{{\rm G}}\left(n\right)}^{a_{{\rm FF}}^{{\rm G}}\left(n\right)\quad a_{{\rm FS}}^{{\rm G}}\left(n\right)} \right ). \\
\end{eqnarray*}
$$

When the game and the learning horizon is clear (or irrelevant), we sometimes drop the indices *G* and *n* for better readability.

#### Evolutionary dynamics in well-mixed populations

Given the payoff matrix, we explore the evolutionary dynamics between learning rules using the replicator equation (78). The replicator equation describes deterministic evolution in infinite populations. Let *x* ∈ [0, 1] denote the frequency of *FMTL* in the population. The frequency of selfish learners is 1 − *x*. Using the payoffs of Eq. (7), the fitness values of the two types are

(8a) $$\begin{eqnarray*}
f_{\textrm {F}}\left(x\right) &= x a_{\textrm {FF}} + \left(1-x\right) a_{\textrm {FS}} ;
\end{eqnarray*}
$$

(8b) $$\begin{eqnarray*}
f_{\textrm {S}}\left(x\right) &= x a_{\textrm {SF}} + \left(1-x\right) a_{\textrm {SS}} ,
\end{eqnarray*}
$$

respectively. The average fitness in the population is $\overline{f}\left(x\right) =xf_{\textrm {F}}\left(x\right) +\left(1-x\right) f_{\textrm {S}}\left(x\right)$. Under replicator dynamics, the frequency of *FMTL* satisfies the ordinary differential equation

(9) $$\begin{eqnarray*}
\frac{dx}{dt} &= x \left( f_{\textrm {F}}\left(x\right) - \overline{f}\left(x\right) \right) .
\end{eqnarray*}
$$

When *a*_FF_ ≥ *a*_SF_ and *a*_FS_ ≥ *a*_SS_, we have *f*_F_(*x*) ≥ *f*_S_(*x*), and thus $f_{\textrm {F}}\left(x\right) \geqslant \overline{f}\left(x\right)$ for all *x* ∈ (0, 1). Moreover, if one of the inequalities is strict, *a*_FF_ > *a*_SF_ or *a*_FS_ > *a*_SS_, then $f_{\textrm {F}}\left(x\right) \gt \overline{f}\left(x\right)$. This payoff relationship holds in prisoner’s dilemmas and stag hunt games, as well as in the snowdrift game with (*S* + *T*)/2 < *R*, when *n* is sufficiently large. In that case, there are two equilibria. The equilibrium *x* = 0 is unstable, whereas *x* = 1 is globally stable. It follows that *FMTL* evolves from all initial populations with *x* > 0. Figure 4(b) illustrates these dynamics for the donation game as the benefit of cooperation varies. It is worth pointing out that the relative value of *a*_SF_ compared to *a*_FS_ does not affect replicator dynamics. More precisely, even though a selfish learner always gets at least the coplayer’s payoff in any interaction with *FMTL* (i.e. even though *a*_SF_ ≥ *a*_FS_ for all games we studied), selfish learning may still go extinct.

#### Evolutionary dynamics in structured populations

The classical replicator equation describes populations in which all individuals are equally likely to interact with each other. To explore how population structure is expected to affect our evolutionary results, we use the approach of Ohtsuki and Nowak (104). They show that under weak selection, various stochastic evolutionary processes on regular graphs can be approximated by a replicator equation with modified payoffs. Instead of *a_ij_*, the payoff of strategy *i* against strategy *j* is now given by $\widetilde{a}_{ij} := a_{ij}+b_{ij}$. Here, *b_ij_* depends on the game, the evolutionary process under consideration, and the degree *k* > 2 of the network. For example, for death-birth updating,

(10) \begin{equation*} b_{ij} = \frac{\left(k+1\right) a_{ii}+a_{ij}-a_{ji}-\left(k+1\right) a_{jj}}{\left(k+1\right)\left(k-2\right)} . \end{equation*}

When we apply this formula to the four payoffs *a*_FF_, *a*_FS_, *a*_SF_, and *a*_SS_, the modified payoffs are

(11a) $$\begin{eqnarray*}
\widetilde{a}_\textrm {FF} &= a_\textrm {FF} ;
\end{eqnarray*}
$$

(11b) $$\begin{eqnarray*}
\displaystyle \widetilde{a}_\textrm {FS} &= a_\textrm {FS}+\frac{\left(k+1\right) a_\textrm {FF}+a_\textrm {FS}-a_\textrm {SF}-\left(k+1\right) a_\textrm {SS}}{\left(k+1\right)\left(k-2\right)} ;
\end{eqnarray*}
$$

(11c) $$\begin{eqnarray*}
\displaystyle \widetilde{a}_\textrm {SF} &= a_\textrm {SF}+\frac{\left(k+1\right) a_\textrm {SS}+a_\textrm {SF}-a_\textrm {FS}-\left(k+1\right) a_\textrm {FF}}{\left(k+1\right)\left(k-2\right)} ;
\end{eqnarray*}
$$

(11d) $$\begin{eqnarray*}
\widetilde{a}_\textrm {SS} &= a_\textrm {SS} .
\end{eqnarray*}
$$

For replicator dynamics, only payoff differences matter. These can be written as follows:

(12a) $$\begin{eqnarray*}
\widetilde{a}_\textrm {FF}-\widetilde{a}_\textrm {SF} &=& \left(1+\frac{1}{\left(k+1\right)\left(k-2\right)}\right)\left(a_\textrm {FF}-a_\textrm {SF}\right) \nonumber \\
&& +\ \frac{1}{\left(k+1\right)\left(k-2\right)}\left(a_\textrm {FS}-a_\textrm {SS}\right) \nonumber \\
&& + \ \frac{k}{\left(k+1\right)\left(k-2\right)}\left(a_\textrm {FF}-a_\textrm {SS}\right) ;
\end{eqnarray*}
$$

(12b) $$\begin{eqnarray*}
\widetilde{a}_\textrm {FS}-\widetilde{a}_\textrm {SS} &=& \left(1+\frac{1}{\left(k+1\right)\left(k-2\right)}\right)\left(a_\textrm {FS}-a_\textrm {SS}\right) \nonumber \\
&& + \ \frac{1}{\left(k+1\right)\left(k-2\right)}\left(a_\textrm {FF}-a_\textrm {SF}\right)\nonumber \\
&& + \ \frac{k}{\left(k+1\right)\left(k-2\right)}\left(a_\textrm {FF}-a_\textrm {SS}\right) .
\end{eqnarray*}
$$

In a well-mixed population, the condition for *FMTL* to be globally stable is *a*_FF_ ≥ *a*_SF_ and *a*_FS_ ≥ *a*_SS_, with at least one inequality being strict. In the examples where we observe *FMTL* to globally stable, the inequality *a*_FF_ > *a*_SS_ is also satisfied (Fig. 4a; Figures S6 and S7). For those games, Eq. (12) allows us to conclude that if *FMTL* is globally stable in a well-mixed population, then it is also stable in any regular network. Moreover, especially if the degree *k* of the network is small, regular networks may require a smaller learning horizon for *FMTL* to become globally stable.

### Empirical analysis of a repeated prisoner’s dilemma experiment

#### Experimental methods

To compare our theoretical predictions to actual human behavior, we reanalyze the experimental data collected by Hilbe et al. (84). In this experiment, human subjects play 60 rounds of a repeated prisoner’s dilemma against a computer program. Humans are not told the nature of their opponent; instead, they only learn that they are “matched with an opponent with whom they will interact for many rounds.” In each round, participants can either cooperate or defect. The payoffs per round are derived from the payoffs used in Axelrod’s tournament (19):

(13) \begin{equation*} R=0.30~\text{EUR};~~~~S=0.00~\text{EUR};~~~~T=0.50~\text{EUR};~~~~P=0.10~\text{EUR}. \end{equation*}

In addition, all participants get a show-up fee (independent of their performance) of 10 EUR. All participants are first-year biology students recruited from the universities of Kiel and Hamburg, Germany.

The experiment consists of four treatments, which only differ in the memory-one strategies implemented by the computer program. The four strategies are referred to as being “strongly extortionate,” “mildly extortionate,” “mildly generous,” and “strongly generous,” and they are specified as follows:

(14) \begin{equation*} \begin{array}{lll}\text{Strongly extortionate:} &p_0=0, &\mathbf {p}=\left(0.692,~0,~0.538,~0\right) ; \\ \text{Mildly extortionate:} &p_0=0, &\mathbf {p}=\left(0.857,~0,~0.786,~0\right) ; \\ \text{Mildly generous:} &p_0=1, &\mathbf {p}=\left(1,~0.077,~1,~0.154\right) ; \\ \text{Strongly generous:} &p_0=1, &\mathbf {p}=\left(1,~0.182,~1,~0.364\right) . \end{array} \end{equation*}

For the given payoff values, these four strategies represent so-called zero-determinant strategies (20,21). By using one of these strategies, the computer program ensures that there is an approximately linear relationship between the payoff of the human participant π_*H*_ and the payoff of the computer program π_*C*_. The respective linear relationships are (84)

(15) \begin{equation*} \begin{array}{lc}\text{Strongly extortionate:} &\pi _H - P = \frac{1}{3} \left(\pi _C - P\right) ; \\ \text{Mildly extortionate:} &\pi _H - P = \frac{2}{3} \left(\pi _C - P\right) ; \\ \text{Mildly generous:} &\pi _H - R = \frac{2}{3} \left(\pi _C - R\right) ; \\ \text{Strongly generous:} &\pi _H - R = \frac{1}{3} \left(\pi _C - R\right) . \end{array} \end{equation*}

The interpretation of these payoff relationships is as follows. If the program is extortionate, the human coplayer’s surplus (over the mutual defection payoff) is only one-third or two-thirds of the computer’s surplus. On the other hand, if the program is generous, the human coplayer’s loss (compared to the payoff for mutual cooperation) is only one-third or two-thirds of the computer’s loss. In particular, an extortionate computer program always obtains at least as much as the human coplayer, whereas a generous program only obtains at most the payoff of the human coplayer (21). In Fig. 5(a), the payoff relationships for the strong strategy variants are indicated by a black dashed line. In the extortionate case, the black dashed line is always on or below the main diagonal. In the generous case, the black dashed line is always on or above the main diagonal. The colored dots represent experimental data, averaged over all participants of the respective treatment. Each dot indicates the payoff of the human participant and the computer opponent for consecutive five-round intervals. Overall, the study has been conducted with 60 participants (16 participants in each of the two strong treatments and 14 participants in each of the two mild treatments).

#### Theoretical predictions

For the interpretation of the experimental results with respect to our theoretical framework, three aspects of the strategies are crucial.

1. According to Eq. (15), there is a positive linear relationship between the payoffs of the computer program and the human participant. In particular, if humans wish to maximize their own payoff, they should strive to maximize their opponent’s payoff. That is, they should cooperate in every single round.
2. In contrast, if humans wish to have equal payoffs π_*H*_ = π_*C*_ against the extortionate program, they should defect in every round (in which case π_*H*_ = π_*C*_ = *P*). If they wish to have equal payoffs against the generous program, humans should cooperate in every round (in which case π_*H*_ = π_*C*_ = *R*).
3. Importantly, the monetary incentives for humans to become more cooperative are the same in the strongly extortionate and in the strongly generous treatment. In either case, for every cent that they increase the coplayer’s payoff by being more cooperative, their own payoff is increased by one-third of a cent. Similarly, the monetary incentives for increasing cooperation in the two mild treatments are also identical.

Because of the first and the third property, we would predict that participants who wish to increase their own payoffs cooperate equally often, independent of whether they are matched with a strongly extortionate or with a strongly generous opponent. An analogous prediction applies to the two mild treatments. In contrast, because of the second property, we would predict that participants who wish to enhance fairness are more likely to cooperate in the generous treatments. These predictions are general; they do not depend on the exact implementation of the participants’ learning rules.

In addition to these qualitative predictions, we have also run simulations for the learning rules studied herein. In Fig. 5(c), we show the average of 1,000 simulations (bold solid lines) as well as a sample of 20 representative simulation runs (thin dotted lines). For each simulation, there is one learner who either implements selfish learning or *FMTL*. Consistent with our theoretical analysis, the learner is restricted to memory-one strategies. Initially, the learner’s strategy is unconditional, with *p*_0_ = 0.35 and $\mathbf {p}=\left(0.35,~0.35,~0.35,~0.35\right)$. In each simulation, the learner is given 100 opportunities to revise its memory-one strategy. New strategies are sampled within an *s* = 0.1-neighborhood of the parent strategy. For *FMTL*, we use a sensitivity parameter of σ = 0.1, as in our main text analysis of the standard prisoner’s dilemma. The learner’s opponent applies a fixed memory-one strategy that is either strongly extortionate or strongly generous, as specified by Eq. (14). Consistent with the qualitative predictions above, we find that selfish learners equally learn to cooperate no matter whether the opponent is extortionate or generous. In contrast, the *FMTL* player only learns to fully cooperate against a generous opponent.

#### Statistical analysis

To test these predictions, we first compare the cooperation dynamics against the strongly extortionate strategy and the strongly generous strategy (participants matched with the strongly extortionate strategy face the strongest trade-off between payoff maximization and fairness). In Fig. 5(b), each dot represents the human participants’ cooperation rates, averaged over three rounds. In particular, the panel shows that initially participants are equally likely to cooperate against both computer programs (31.3% during the first three rounds for both). However, only for the strong generosity treatment is there a significant increase in cooperation rates (to 85.4% during the last three rounds; Wilcoxon matched-pairs signed-rank test: *Z* = 2.9341, *P* = 0.003, all tests are two-tailed). In contrast, in the strong extortion treatment, there is no such increase (33.3%; Wilcoxon matched-pairs signed-rank test: *Z* = 0.3494, *P* = 0.726.).

We obtain similar results if we compare overall cooperation rates over all 60 rounds (Fig. 5d). Against the strongly extortionate program, this average cooperation rate is 29.6%, compared to 70.3% against the strongly generous program (Mann–Whitney U test, *Z* = 2.789, *P* = 0.005). For the two mild treatments, the difference in cooperation rates is smaller, and fails to be significant; however, players still tend to cooperate more against the generous program (the cooperation rates are 39.5% and 64.8%, Mann–Whitney U test, *Z* = 1.860, *P* = 0.063). Overall, these results suggest that fairness considerations affect human cooperation rates. Moreover, this effect is more pronounced the stronger the trade-off between payoff maximization and fairness.

## Supplementary Material

pgac141_Supplemental_Files

## Data Availability

Supporting code is available at https://github.com/alexmcavoy/fmtl/.
