# Supplementary material for: Evolutionary instability of selfish learning in repeated games
Source: PNAS Nexus. 2022 Jul 27;1(4):pgac141. doi: 10.1093/pnasnexus/pgac141 (PMC9802390; doi:10.1093/pnasnexus/pgac141)
Supplement: pgac141_Supplemental_Files [file pgac141_supplemental_files.zip › si.pdf]

Supporting Information
Evolutionary instability of selfish learning in repeated games

Alex McAvoy[1,2], Julian Kates-Harbeck[3], Krishnendu Chatterjee[4], and Christian Hilbe[5]

[1]Department of Mathematics, University of Pennsylvania, Philadelphia, PA, USA;
[2]Center for Mathematical Biology, University of Pennsylvania, Philadelphia, PA, USA;
[3]Department of Physics, Harvard University, Cambridge, MA, USA;
[4]Institute of Science and Technology Austria, Klosterneuburg, Austria;
[5]Max Planck Research Group: Dynamics of Social Behavior, Max Planck Institute for Evolutionary Biology, Plön, Germany

S1 Related literature

Our study explores how different learning rules affect the dynamics of two-player interactions. It is motivated by previous work in three fields, the literature on evolution in repeated games, the literature on social preferences, and the literature on multi-agent learning. In the following, we discuss each of these three fields in more detail.

S1.1 Related work on evolution in repeated games

There is a rich literature on evolution in repeated games. A general summary of this literature can be found in the books by Axelrod (1), Nowak (2), and Sigmund (3), or in recent review articles (4, 5). Research in this area explores three broad themes. *(i)* It aims to identify strategies that can sustain cooperation (6–13). *(ii)* It describes how likely such cooperative strategies evolve (14–20) and persist (21–24) in a population. *(iii)* It evaluates to which extent the predicted strategies can be observed in humans (25–28) or other animal species (29). While our study speaks to all three aspects mentioned above, we are most concerned with a more general question. We ask how individuals themselves can learn to adopt beneficial strategies more effectively, when given the choice between different learning rules.

Previous research on evolution in repeated games presumes that individuals engage in what we have termed "selfish learning." Selfish learners evaluate the performance of their strategies by the immediate payoffs they yield. Herein, we argue that selfish learning may be replaced by alternative learning rules if selection operates on a learning rule's long-run performance. The selection pressure for an alternative learning rule may be particularly strong in games with multiple equilibria. In such games, selfish learning can lead individuals to settle at inefficient outcomes although more profitable equilibria are available. Such inefficiencies can occur in the

repeated prisoner's dilemma (4) but also in any other social dilemma that requires coordination (30–33).

Within the literature on repeated games, our work is most closely related to studies that explore properties of memory-one strategies (34–43). For the repeated prisoner's dilemma without discounting, Akin (38, 39) was first to describe all memory-one strategies that stabilize full cooperation in a Nash equilibrium. This work was extended by Stewart and Plotkin (36) to describe all symmetric Nash equilibria. In equilibrium, only four outcomes are feasible: mutual cooperation, mutual defection, alternating cooperation, and equilibria that are enforced by so-called equalizer strategies (44). Because Nash equilibria result in outcomes where no player can unilaterally increase their payoff, any such equilibrium is a rest point of the learning dynamics among two selfish learners. When players can switch to any other memory-one strategy, the converse is also true: any rest point of the learning dynamics among two selfish learners must be a Nash equilibrium.

However, similar to the literature on adaptive dynamics (45, 46), we focus on players who adapt locally. This means that in each updating step, learners make only minor modifications to their existing strategies. In that case, two selfish learners may get stuck in rest points that are locally stable without being Nash equilibria. Local adaptation leads to "smoother" trajectories of the learning dynamics. However, as shown by Stewart and Plotkin (47), the qualitative outcomes are comparable to those of global updating. Again, eventually players either mutually cooperate, defect, alternate, or they employ equalizer strategies. However, simulations suggest that they become even more likely to settle at mutual defection (47). Selfish learning is therefore particularly prone to result in inefficient outcomes under local adaptation.

Overall, we contribute to this literature as follows:

1. We introduce a framework to formalize learning in repeated games. While we focus on one learning rule in particular (*FMTL*), our notion of learning rules is general, and it can be used to describe many alternative modes of learning.
2. We explore the learning dynamics that transpire when players have objectives that are different from immediate payoff maximization. With *FMTL*, we identify a learning rule that shows a surprisingly strong performance across many different types of games. In these games, *one FMTL* learner is usually sufficient for *both* players to be better off eventually.
3. Our research clarifies under which conditions selfish learning can be expected to persist in a population. We find that the evolutionary robustness of selfish learning depends on the game being played, and on the relative timescale of evolution. When learning rules evolve at a similar rate as the learners' strategies, selfish learning is prevalent. However, when learning rules evolve at a much slower timescale, selfish learning is dominated in most games we consider. The only exceptions arise when selfish learning generally results in efficient outcomes, as in the hero game and in certain snowdrift games.

S1.2 Related work on social preferences

In addition to the evolutionary game theory literature, our work is related to the branch of behavioral economics that explores social (or other-regarding) preferences. Research in this area is

centered around the following three themes. *(i)* It explores experimentally to which extent humans take the payoffs of other group members into account when making decisions. *(ii)* It aims to integrate these empirically estimated preferences into standard models of economic decision making. *(iii)* It describes how humans may have developed social preferences over an evolutionary timescale. For a general overview of this literature and a critical evaluation, we refer to the reviews by Fehr and Schmidt (48), List (49), and Alger and Weibull (50).

Social preferences can manifest themselves in various forms. Decision makers may aim to increase the payoff of the group member with the lowest payoff ("maximin preferences"); they may strive to have the highest payoff within their group ("competitive preferences"); they may want to maximize the joint payoff of all group members ("efficiency preferences"); or they may wish to reduce economic inequality within their group ("inequity aversion," which is usually interpreted as a preference for fairness). To the extent that decision-makers are willing to pay a personal cost to achieve these objectives, their preferences represent deviations from the classical *homo economicus*.

In the context of our study, the last two of the above-mentioned preferences are particularly relevant–those related to fairness and efficiency. In the following, we first discuss some of the experimental evidence for the existence of such preferences. In a next step, we briefly review the existing theoretical literature on preference evolution.

S1.2.1 Experimental evidence for fairness and efficiency preferences

There is a rather extensive literature suggesting that humans value egalitarian outcomes (51, 52). One piece of evidence comes from experiments on ultimatum-bargaining (53). Here, participants typically reject mutually beneficial offers if these offers put them at a notable disadvantage. Another piece of evidence comes from experiments on public good games with punishment (54). Here, a non-negligible fraction of participants are willing to pay a cost to reduce the payoff of free-riders, even if any long-run advantage of punishment is excluded by the experimental design. While there is considerable cross-cultural variation in the extent to which humans reject unfair offers and retaliate against free-riders (55, 56), an aversion against disadvantageous inequity seems to be universal (57) and it is developed by children at a relatively young age (58). The brain regions that are activated when rejecting unfair offers include the ventral striatum and the ventromedial prefrontal cortex (59). The former is associated with social comparisons, whereas the latter integrates costs and benefits into a decision value (52).

Preferences for inequity aversion can be integrated into economic models by extending the individual's utility functions. While the precise specifications differ between studies (60, 61), these models usually represent utilities as a linear combination of the individual's own payoff and some measure of group inequality. By calculating the Nash equilibria for the modified utility functions, these models can be used to make experimental predictions.

There is similar research on the role of efficiency in human decision making. For example, Andreoni and Miller (62) study different variants of a dictator game. In their experiments, a randomly assigned participant (the "dictator") decides how to divide a fixed number of tokens between herself and a recipient. Different treatments vary the relative worth of tokens. In some treatments, tokens are more valuable to the dictator; in others they are more valuable to the recipient. The study finds that there is considerable heterogeneity among dictators. However,

a substantial number of dictators give more tokens to the player to whom tokens are worth the most (even if this happens to be the other player). This may be taken as evidence that such dictators value efficiency. Similar qualitative findings are reported by Charness and Rabin (63) and by Engelmann and Strobel (64). As with inequity aversion, preferences for efficiency can be incorporated into standard models of game theory by considering appropriately parameterized utility functions (62, 63).

S1.2.2 Indirect evolutionary approach

Individuals with social preferences may adopt strategies that do not maximize their material payoff. If one assumes that evolutionary processes select for payoff-maximizing behavior, the question arises how social preferences can evolve. To address this question, researchers have adopted the "indirect evolutionary approach" (65, 66). Similar to our evolutionary model, this literature considers dynamics on two timescales. In the short run, the players' social preferences are fixed. Given these preferences, individuals coordinate on an equilibrium that maximizes their subjective utilities. In the long run, the players' preferences evolve. For the evolution of preferences, material payoffs are relevant. Only those preferences that yield high material payoffs in equilibrium proliferate. For a detailed review of this literature, see Alger and Weibull (50). In the following, we describe some of the key insights.

The indirect evolutionary approach has identified two major mechanisms that allow social preferences to be evolutionarily stable. The first mechanism is based on the observation that social preferences can serve as a commitment device (e.g. Refs. (65–68)). The respective models assume (sometimes implicitly) that a player's preference type is publicly observable. In this "complete information" scenario, preferences affect a player's payoff in two ways. *(i)* They have a direct effect as they influence which action the player will choose. *(ii)* They have an indirect effect as they influence how the player's opponent will react. While the direct effect has negative consequences on the player's payoff, the indirect effect can be positive. Because the direct payoff effect can be expected to be vanishingly small for sufficiently small deviations from payoff-maximizing behavior, social preferences can evolve (67).

As an example of such a setup, Akçay et al. (68) consider a model where two individuals share resources with each other. Each individual's material payoff is monotonically decreasing in the amount of resource given away, and monotonically increasing in the amount of resource received. Players with other-regarding preferences share more than they would in a Nash equilibrium. Because of the assumed multiplicative form of the players' preference functions, this also induces the second player to share more of their resource. As long as the overall effect is positive, population members have an incentive to extend the magnitude of their other-regarding preferences. Interestingly, the eventual equilibrium can only be sustained *because* the players have evolved other-regarding preferences. This is somewhat different from what happens in our model. In all examples we have studied, the eventual equilibrium payoffs of two *FMTL* learners are also viable equilibrium payoffs among two selfish players. However, *FMTL* makes it more likely that two learners would coordinate on the respective fair and efficient equilibria.

The second (and more recently explored) mechanism that can give rise to social preferences is population structure. When players with similar preferences are more likely to interact with each other, evolution may give rise to behavior consistent with *Homo moralis* (69). For pairwise

interactions, this type of player maximizes a linear combination of the material payoff and the payoff the player would obtain if the co-player adopted the same strategy. While we focus on well-mixed populations throughout most of the main text, we describe in the **Methods** section that population structure can also have a positive effect within our learning framework.

Our work adds to this literature as follows:

1. Existing theoretical models on social preferences use a static perspective to describe the strategic interaction (i.e. the short run dynamics). They introduce modified utility functions to explore the effect on the game's Nash equilibria. To make clear predictions, this approach often requires the respective equilibria to be unique.
   Instead, we are interested in a dynamic theory of learning. In our framework, players do not play equilibrium strategies from the outset, but they adapt their strategies over time. Learning rules do not only affect whether a given payoff allocation can be achieved in equilibrium. Instead, they also affect how likely players are to coordinate on each possible equilibrium outcome.
2. Because the existing literature on the indirect evolutionary approach focuses on equilibrium behavior, they typically assume a complete separation of timescales; by the time preferences evolve, the players' strategies have reached an equilibrium. Instead, we add to this literature by exploring how the players' learning horizon affects the long-run evolutionary dynamics.
3. The experimental literature on social preferences highlights the role of fairness and efficiency for human decision making. We show that these two objectives can be combined to create a learning rule that exhibits a remarkable performance in the context of multi-agent learning.

S1.3 Related work on multi-agent learning

In addition to its relationship to the social sciences, our work touches upon topics of interest in the field of multi-agent learning. The literature on single- and multi-agent reinforcement literature is vast; here, we briefly mention several studies that are closely related. For general overviews of the basic goals of multi-agent learning, as well as its relationship to evolutionary game theory, we refer the reader to Refs. (70–73).

Optimization problems involving a single agent in a stationary environment are usually approached using the well-studied theory of Markov decision processes, where algorithms have strong performance guarantees. Multi-agent learning introduces two key complications (72, 74), which are captured by our simplified model. First, the presence of other agents, all of which can perform actions influencing each other, and all of which are actively learning and changing their own behavior, introduces a "moving target" aspect to the environment, where the outcome of the learning algorithm of one agent is interdependent with the learning trajectory of the other agent (75). The environment is no longer stationary or Markovian. In the multiplied learning setting considered in this paper (as opposed to divided or active learning) each agent acts on their own and is not necessarily aware of or aligned with the goals and algorithms of other agents. Since

increased awareness allows for simpler solutions and direct incorporation of other agents' objective functions (76), which may not be possible in real-life settings, we assume agents to be mutually unaware.

Second, the behavior of the other agents cannot necessarily be predicted based on simple assumptions. Unlike fully cooperative scenarios where all agents have aligned goals, or fully competitive zero-sum games, where one can reason unambiguously about the best response of adversaries (77–79) and make progress with methods such as tree-search or self-play, most real world scenarios combine both cooperative and adversarial components and incentive alignment is unclear or variable. As such, a degree of coordination with the other player and their learning trajectory is necessary to achieve outcomes that maximize both payoffs while limiting exploitation. In this new regime, the optimal choice of objective function for the learner is unclear. How to align incentives and find globally optimal outcomes in this general/unaligned case remains poorly understood (70).

Many approaches to multi-agent learning in the context of repeated social dilemmas assume multiple self-interested agents as a standard starting point (80). An example is infinitesimal gradient ascent (including variants such as "win or learn fast," where learning rate is high when algorithm is losing, otherwise low (81)), which follows the gradient of expected payoffs and is guaranteed to converge to the Nash equilibrium of two-player, two-action games. (In our study, even when discussing selfish learning, our primary focus is on optimization via random search, as opposed to gradient-based techniques.) Another example is Nash-Q, an exemplary algorithm for multi-agent learning, which aims to find Nash equilibria (82). While we note that a high b/c ratio can give rise to increasingly favorable outcomes for self-interested agents (see Figure 2), self-interested agents and Nash equilibria can result in globally poor outcomes in the general case, especially in social dilemmas.

With that being said, in the same way that the idea of social preferences in behavioral economics has been around for decades, considering the opponent's score in multi-agent optimization is not new. A simple example is a learning rule called "cooperative reward shaping" (83), which blends selfish objectives with a desire to increase efficiency. Simple convex combinations of the players' payoffs has also proven effective in coordination games such as the stag hunt (84). Inequity aversion, based on the model of Fehr and Schmidt (60), has been shown to promote cooperation in temporally-extended social dilemmas (85). A slightly different approach is to incorporate the opponent's learning process directly into one's own ("learning with opponent-learning awareness" (76)), provided this information is available. In fact, one could readily conjecture that other-regarding preferences do (and will) play an important role in algorithms based on the fact that humans, a species subject to evolutionary pressures, face similar challenges to those encountered in multi-agent learning and are documented to have social preferences.

The authors of Ref. (70) describe five agendas in the field of multi-agent learning: computational, descriptive, normative, prescriptive (cooperative), and prescriptive (non-cooperative). Our work would be best described as descriptive and prescriptive (non-cooperative). It is descriptive, at least partially, in that the learning rules we consider are inspired by behavioral experiments, in which humans are known to care about (and even balance) fairness and efficiency. In that respect, we are concerned with whether such behaviors can drive selfishness out of a population due their abilities to shape the incentives of a selfish learner. Our work is prescriptive, in the non-cooperative sense, because it addresses the question of how a learner can/ought to behave

in order to overcome the drawbacks of selfish learning (since selfish learning is inefficient in non-cooperative games).

As it relates to the multi-agent learning literature, our contribution can be summarized as follows:

1. We present a novel learning rule, which balances objectives of fairness and efficiency in a dynamic fashion and achieves excellent outcomes against selfish learning across a variety of games.
2. We study "supergames" of learning rules. When trying to understand competition between learning rules in a population, one needs to know the outcomes of all possible combinations of learning rules. This kind of analysis can allow one to make deductions about evolutionary dynamics without studying a specific kind of update rule (e.g. for dominance games). We study this supergame and its qualitative transitions (e.g. bistable competition, coexistence, and dominance games) as a function of the timescale of learning.
3. We show that evolutionary models do not take into account aspects of learning rules that might be nonetheless important in settings of multi-agent learning. While fairness is important for eventually arriving at outcomes with high payoffs, on average, evolutionary dynamics often do not take into account inequity. The hero game, as well as a variant of the snowdrift game, illustrate this point clearly: *FMTL* is effectively neutral relative to selfish learning (Figure S8), yet *FMTL* is able to elicit fair outcomes against an opponent with near certainty, while two selfish learners often end up with unequal payoffs (Figure S5).

S2 An analysis of local strategy revisions

In our study, we consider learning processes among players with memory-one strategies in repeated two-player interactions. To this end, we consider players who adapt *locally*. In each learning step, the respective player compares its current strategy $\mathbf{p}$ with an alternative strategy $\mathbf{p}'$ that is sufficiently nearby. The alternative strategy is adopted if it leads to an improvement, given the player's objectives. In the following, we ask under which conditions such local improvements are possible. This analysis will help us to characterize which strategy pairs $(\mathbf{p}, \mathbf{q})$ are rest points of the learning dynamics. Rest points are those strategy profiles for which both players are unable to make any further local improvements (given their objectives). We begin our analysis by deriving some useful properties of memory-one strategies in repeated games.

S2.1 Game setup and some useful lemmas

We consider two players, X and Y, with memory-one strategies $\mathbf{p} = (p_0, p_{CC}, p_{CD}, p_{DC}, p_{DD})$ and $\mathbf{q} = (q_0, q_{CC}, q_{CD}, q_{DC}, q_{DD})$, respectively. They repeatedly interact in a game with one-shot payoffs $\mathbf{u}_X = (R, S, T, P)$ and $\mathbf{u}_Y = (R, T, S, P)$. Future payoffs are discounted by $\lambda < 1$. The players' payoffs for the repeated game can thus be written as in Equation **3** of the main text,

$$\pi_X(\mathbf{p}, \mathbf{q}) = \langle \mathbf{v}, \mathbf{u}_X \rangle ; \qquad \text{(S1a)}$$

$$\pi_Y(\mathbf{p}, \mathbf{q}) = \langle \mathbf{v}, \mathbf{u}_Y \rangle . \qquad \text{(S1b)}$$

Here, the vector $\mathbf{v}$ is given by

$$\mathbf{v} = (v_{CC}, v_{CD}, v_{DC}, v_{DD}) := (1-\lambda)\, v_0(\mathbf{p},\mathbf{q}) \left(I - \lambda M(\mathbf{p},\mathbf{q})\right)^{-1}, \tag{S2}$$

where

$$v_0(\mathbf{p},\mathbf{q}) = \Big(p_0 q_0,\ p_0(1-q_0),\ (1-p_0)\, q_0,\ (1-p_0)(1-q_0)\Big) \tag{S3}$$

and

$$M(\mathbf{p},\mathbf{q}) = \begin{pmatrix} p_{CC}q_{CC} & p_{CC}(1-q_{CC}) & (1-p_{CC})\,q_{CC} & (1-p_{CC})(1-q_{CC}) \\ p_{CD}q_{DC} & p_{CD}(1-q_{DC}) & (1-p_{CD})\,q_{DC} & (1-p_{CD})(1-q_{DC}) \\ p_{DC}q_{CD} & p_{DC}(1-q_{CD}) & (1-p_{DC})\,q_{CD} & (1-p_{DC})(1-q_{CD}) \\ p_{DD}q_{DD} & p_{DD}(1-q_{DD}) & (1-p_{DD})\,q_{DD} & (1-p_{DD})(1-q_{DD}) \end{pmatrix}. \tag{S4}$$

For $\lambda < 1$, these payoffs, $\pi_X(\mathbf{p},\mathbf{q})$ and $\pi_Y(\mathbf{p},\mathbf{q})$, are well-defined for all $\mathbf{p},\mathbf{q} \in [0,1]^5$, and they fall within the *feasible region* of the game, which is defined as the convex hull of the players' one-shot payoffs, namely (R,R), (S,T), (T,S), and (P,P). In the following, it will be useful to have an alternative expression for the repeated game payoffs. This alternative expression is somewhat more explicit, as it does not require computing the inverse of a matrix. The following result is by Mamiya and Ichinose (42), who themselves use the framework by Press and Dyson (34).

Lemma 1 (An alternative representation of payoffs)
For $\mathbf{u} = (u_1,u_2,u_3,u_4) \in \mathbb{R}^4$ *and* $\mathbf{p},\mathbf{q} \in [0,1]^5$*, let* $A(\mathbf{p},\mathbf{q},\mathbf{u})$ *denote the matrix*

$$\begin{pmatrix} (1-\lambda)\,p_0q_0 + \lambda p_{CC}q_{CC} - 1 & (1-\lambda)\,p_0 + \lambda p_{CC} - 1 & (1-\lambda)\,q_0 + \lambda q_{CC} - 1 & u_1 \\ (1-\lambda)\,p_0q_0 + \lambda p_{CD}q_{DC} & (1-\lambda)\,p_0 + \lambda p_{CD} - 1 & (1-\lambda)\,q_0 + \lambda q_{DC} & u_2 \\ (1-\lambda)\,p_0q_0 + \lambda p_{DC}q_{CD} & (1-\lambda)\,p_0 + \lambda p_{DC} & (1-\lambda)\,q_0 + \lambda q_{CD} - 1 & u_3 \\ (1-\lambda)\,p_0q_0 + \lambda p_{DD}q_{DD} & (1-\lambda)\,p_0 + \lambda p_{DD} & (1-\lambda)\,q_0 + \lambda q_{DD} & u_4 \end{pmatrix}. \tag{S5}$$

The payoffs to players X and Y, according to Equation **S1***, can then be written as*

$$\pi_X(\mathbf{p},\mathbf{q}) = \frac{\det A(\mathbf{p},\mathbf{q},\mathbf{u}_X)}{\det A(\mathbf{p},\mathbf{q},\mathbf{1})}; \tag{S6a}$$

$$\pi_Y(\mathbf{p},\mathbf{q}) = \frac{\det A(\mathbf{p},\mathbf{q},\mathbf{u}_Y)}{\det A(\mathbf{p},\mathbf{q},\mathbf{1})}, \tag{S6b}$$

where $\mathbf{1} \in \mathbb{R}^4$ *is the vector of ones.*

As shown by McAvoy and Nowak (43) for games without discounting, a certain monotonicity property holds if only one component of player X's strategy is varied at a time. To explore local strategy revisions, here we first generalize their result to discounted games. To this end, it will be useful to introduce the basis vectors

$$\mathbf{e}_0 = (1,0,0,0,0)\,; \tag{S7a}$$

$$\mathbf{e}_{CC} = (0,1,0,0,0)\,; \tag{S7b}$$
$$\mathbf{e}_{CD} = (0,0,1,0,0)\,; \tag{S7c}$$
$$\mathbf{e}_{DC} = (0,0,0,1,0)\,; \tag{S7d}$$
$$\mathbf{e}_{DD} = (0,0,0,0,1)\,. \tag{S7e}$$

These vectors allow us to introduce the notion of marginal functions, which track how the payoffs to X and Y change as X varies just one parameter of his or her strategy, $\mathbf{p}$. If X varies only p_0, then we have the marginal functions $\varphi_0(z) := \pi_X(\mathbf{p}+z\mathbf{e}_0,\mathbf{q})$ for X and $\psi_0(z) := \pi_Y(\mathbf{p}+z\mathbf{e}_0,\mathbf{q})$ for Y. If X varies only p_{xy} for $x,y \in \{C,D\}$, then we have the marginal functions $\varphi_{xy}(z) := \pi_X\left(\mathbf{p}+z\mathbf{e}_{xy},\mathbf{q}\right)$ for X and $\psi_{xy}(z) := \pi_Y\left(\mathbf{p}+z\mathbf{e}_{xy},\mathbf{q}\right)$ for Y. We note that φ_0 and ψ_0 are well-defined for all $z \in I_0 := [-p_0, 1-p_0]$. Similarly, for $x,y \in \{C,D\}$, both φ_{xy} and ψ_{xy} are well-defined for all $z \in I_{xy} := \left[-p_{xy}, 1-p_{xy}\right]$.

Lemma 2
Consider a game with arbitrary one-shot payoffs $\mathbf{u}_X$ and $\mathbf{u}_Y$, respectively. Then, for $x,y \in \{C,D\}$, the sets $\left\{\left(\psi_0(z),\varphi_0(z)\right)\right\}_{z\in I_0}$ and $\left\{\left(\psi_{xy}(z),\varphi_{xy}(z)\right)\right\}_{z\in I_{xy}}$ are line segments within the feasible region. Furthermore, the functions $\varphi_0(z)$, $\psi_0(z)$, $\varphi_{xy}(z)$, and $\psi_{xy}(z)$ vary monotonically in z. In particular, as z increases, movement is possible in at most one direction along these line segments.

Proof. The fact that $\{(\psi_0(z),\varphi_0(z))\}_{z\in I_0}$ forms a line segment in the feasible region is immediate from Equations **S1–S3** since p_0 (and therefore z) enters only into the initial distribution over the states. Because the respective functions are linear in z, the monotonicity property also follows immediately.

For $\left\{\left(\psi_{xy}(z),\varphi_{xy}(z)\right)\right\}_{z\in I_{xy}}$, we note that by Equation **S6** (and the fact that the denominators for π_X and π_Y are identical), there exist $\alpha_1,\alpha_2,\beta_1,\beta_2,\gamma_1,\gamma_2$, all independent of z (but depending on $x,y \in \{C,D\}$), such that

$$\varphi_{xy}(z) = \frac{\alpha_1 z+\alpha_2}{\beta_1 z+\beta_2}; \tag{S8a}$$
$$\psi_{xy}(z) = \frac{\gamma_1 z+\gamma_2}{\beta_1 z+\beta_2}. \tag{S8b}$$

A straightforward calculation then gives

$$\left(\beta_1\gamma_2-\beta_2\gamma_1\right)\varphi_{xy}(z) = \left(\alpha_2\beta_1-\alpha_1\beta_2\right)\psi_{xy}(z)+\alpha_1\gamma_2-\alpha_2\gamma_1, \tag{S9}$$

which establishes that $\left\{\left(\psi_{xy}(z),\varphi_{xy}(z)\right)\right\}_{z\in I_{xy}}$ is also a line segment. Finally, to see that one cannot move in two directions along these segments as z increases, we note that $\varphi'_{xy}(z)$ and $\psi'_{xy}(z)$ have the same signs as $\alpha_1\beta_2-\alpha_2\beta_1$ and $\gamma_1\beta_2-\gamma_2\beta_1$, respectively, which are independent of z. □

S2.2 Local strategy revisions

In the following, we ask under which conditions a player is able to locally find profitable deviations from its current strategy (where "profitable" needs to be interpreted in terms of the player's

objectives). To this end, consider an arbitrary but fixed base game with payoff vectors $\mathbf{u}_X$ and $\mathbf{u}_Y$. As usual, we denote the player's current strategies by $\mathbf{p}$ (for player X) and $\mathbf{q}$ (for player Y). Without loss of generality, we assume that it is player X who considers revising his or her strategy.

S2.2.1 Local improvements

Definition 1 (Local improvements)
We say that player X can locally improve its objective function V if for all distances $d > 0$ there is a strategy $\mathbf{p}'$ with $\|\mathbf{p}' - \mathbf{p}\| < d$ such that $V(\mathbf{p}', \mathbf{q}) > V(\mathbf{p}, \mathbf{q})$.

In this definition, it does not matter which exact norm $\|\cdot\|$ we use. However, to be consistent with the simulations, we will usually interpret it as the uniform norm. As a consequence, the relation $\|\mathbf{p}' - \mathbf{p}\| < d$ holds if and only if the difference in each entry is at most d in absolute value.

Trivially, local improvements are only possible if the current value of $V(\mathbf{p}, \mathbf{q})$ is not optimal already. To formalize this observation, we define player X's *maximum attainable value with respect to objective V* as

$$V^*(\mathbf{q}) = \max_{\mathbf{p} \in [0,1]^5} V(\mathbf{p}, \mathbf{q}). \tag{S10}$$

Similarly, we say $\mathbf{p}^*$ is a *best response with respect to objective V* if it is an element of the set

$$\mathrm{BR}_V(\mathbf{q}) = \left\{ \mathbf{p} \in [0,1]^5 \;\middle|\; V(\mathbf{p}, \mathbf{q}) = V^*(\mathbf{q}) \right\}. \tag{S11}$$

We note that for $\mathbf{p} \notin \mathrm{BR}_V(\mathbf{q})$, the task of improving player X's objective function would be simple if global mutations were considered. In this case the player could always switch to some best response $\mathbf{p}^* \in \mathrm{BR}_V(\mathbf{q})$ (or by continuity, to any other strategy that is sufficiently close to $\mathbf{p}^*$).

To identify a local improvement, a natural approach may then be to study interpolations of the form $\mathbf{p}'(z) = (1-z)\,\mathbf{p} + z\mathbf{p}^*$ for $z \in [0,1]$. These interpolations have two useful properties: *(i)* they approach a best response for $z \to 1$ and *(ii)* they are arbitrarily close to the player's current strategy $\mathbf{p}$ for $z \to 0$. For such an interpolation approach to work, however, one would need to prove that $V(\mathbf{p}'(z), \mathbf{q})$ is a monotonic function of z. Unfortunately, this turns out to be not quite how $V(\mathbf{p}'(z), \mathbf{q})$ behaves in general: even if player X can improve its objective function V by taking a large step into the direction of $\mathbf{p}^*$, the value of V may drop when steps are too small.

However, as shown in **Lemma 2**, such a monotonicity property may hold if only one component of player X's strategy is varied at a time (i.e. if we consider the respective marginal functions). To do so, we first consider a special class of objectives.

S2.2.2 Weighted payoff objectives

In the following, we restrict ourselves to objectives that are related to weighted sums of the players' payoffs. For given payoff weights $\boldsymbol{\kappa} = (\kappa_X, \kappa_Y)$, we define the associated objective by

$$V_{\boldsymbol{\kappa}}(\mathbf{p}, \mathbf{q}) = \kappa_X \pi_X(\mathbf{p}, \mathbf{q}) + \kappa_Y \pi_Y(\mathbf{p}, \mathbf{q}). \tag{S12}$$

As special cases, we recover the objective of a selfish learner by setting $\boldsymbol{\kappa} = (1,0)$ and the objective of an efficiency-minded player by setting $\boldsymbol{\kappa} = (1,1)$. To capture the objective function of a fairness-minded player, we consider $-\left|V_{\boldsymbol{\kappa}}(\mathbf{p},\mathbf{q})\right|$, where $\boldsymbol{\kappa} = (1,-1)$.

Because $V_{\boldsymbol{\kappa}}$ is linear in the players' payoffs, and because of the basic properties of scalar products, we can use Equation **S1** to derive more explicit expression for these objective functions,

$$\begin{aligned} V_{\boldsymbol{\kappa}}(\mathbf{p},\mathbf{q}) &= \kappa_X \langle \mathbf{v}, \mathbf{u}_X \rangle + \kappa_Y \langle \mathbf{v}, \mathbf{u}_Y \rangle \\ &= \langle \mathbf{v}, \kappa_X \mathbf{u}_X + \kappa_Y \mathbf{u}_Y \rangle, \end{aligned} \tag{S13}$$

where $\mathbf{v}$ is as defined by Equation **S2**. Equivalently, due to Equation **S6** and the fact that the determinant is a linear function of each column of a matrix, we can write

$$\begin{aligned} V_{\boldsymbol{\kappa}}(\mathbf{p},\mathbf{q}) &= \kappa_X \frac{\det A(\mathbf{p},\mathbf{q},\mathbf{u}_X)}{\det A(\mathbf{p},\mathbf{q},\mathbf{1})} + \kappa_Y \frac{\det A(\mathbf{p},\mathbf{q},\mathbf{u}_Y)}{\det A(\mathbf{p},\mathbf{q},\mathbf{1})} \\ &= \frac{\det A(\mathbf{p},\mathbf{q},\kappa_X \mathbf{u}_X + \kappa_Y \mathbf{u}_Y)}{\det A(\mathbf{p},\mathbf{q},\mathbf{1})}. \end{aligned} \tag{S14}$$

where $A(\mathbf{p},\mathbf{q},\mathbf{u})$ is the matrix defined by Equation **S5**.

Remark 1. By comparing Equation **S13** and Equation **S14** for player X's objective function with Equation **S1** and Equation **S6** for the player's payoff, we identify a useful equivalence: a player who wishes to maximize $V_{\boldsymbol{\kappa}}$ given one-shot payoffs $\mathbf{u}_X$ is equivalent to a player who wishes to maximize π_X for a game with modified one-shot payoffs $\mathbf{u}_{\boldsymbol{\kappa}} := \kappa_X \mathbf{u}_X + \kappa_Y \mathbf{u}_Y$.

S2.2.3 Marginal functions

Definition 2 (Marginal functions with respect to a player's objective V)
Consider two players with strategies $\mathbf{p}$ and $\mathbf{q}$, and suppose player X's objective function is V. Let $I_0 := [-p_0, 1-p_0]$ and, for $x,y \in \{C,D\}$, let $I_{xy} := \left[-p_{xy}, 1-p_{xy}\right]$.

1. *The marginal function $F_0 : I_0 \to \mathbb{R}$ with respect to player X's initial cooperation probability is defined as $F_0(z) := V(\mathbf{p} + z\mathbf{e}_0, \mathbf{q})$.*
2. *Similarly, for each action profile (x,y) with $x,y \in \{C,D\}$ we define a respective marginal function $F_{xy} : I_{xy} \to \mathbb{R}$ by $F_{xy}(z) := V\left(\mathbf{p} + z\mathbf{e}_{xy}, \mathbf{q}\right)$.*

Marginal functions reflect how payoff differences change as player X varies a single entry of his or her strategy. By definition $F_0(0) = F_{xy}(0) = V(\mathbf{p},\mathbf{q})$ corresponds to the status quo. In the special case of weighted payoff objectives, we indeed observe that the associated marginal functions satisfy a monotonicity property:

Lemma 3 (Monotonicity of marginal functions with respect to weighted payoff objectives)
For given payoffs weights $\boldsymbol{\kappa} = (\kappa_X, \kappa_Y)$, each marginal function with respect to the weighted payoff objective $V_{\boldsymbol{\kappa}}$ is either strictly monotonically increasing, strictly monotonically decreasing, or constant on its entire domain.

Proof. The result follows from **Remark 1**, by applying **Lemma 2** to the game in which player X's one-shot payoffs are given by $\mathbf{u}_{\boldsymbol{\kappa}} = \kappa_X \mathbf{u}_X + \kappa_Y \mathbf{u}_Y$. □

The following two Lemmas make use of the above monotonicity property. They show that quite stringent conditions need to hold for player X not to be able to locally improve a weighted payoff objective function:

Lemma 4

Suppose player X's current objective function is $V_{\boldsymbol{\kappa}}$ for some given $\boldsymbol{\kappa} = (\kappa_X, \kappa_Y)$, and consider players with strategies $\mathbf{p}$ and $\mathbf{q}$ such that $\mathbf{p} \notin BR_{V_{\boldsymbol{\kappa}}}(\mathbf{q})$. Moreover, suppose player X is unable to locally improve its objective function.

1. *If $p_0 = 0$, then $F_0(z)$ is either constant or monotonically decreasing in z. If $p_0 = 1$, then $F_0(z)$ is either constant or monotonically increasing. If $0 < p_0 < 1$, then $F_0(z)$ is constant on its entire domain.*
2. *Analogously, if $p_{xy} = 0$ for $x, y \in \{C, D\}$, then $F_{xy}(z)$ is either constant or monotonically decreasing in z. If $p_{xy} = 1$, then $F_{xy}(z)$ is either constant or monotonically increasing. If $0 < p_{xy} < 1$, then $F_{xy}(z)$ is constant on its entire domain.*

Proof. Suppose $F_0(z)$ is not constant. Because of **Lemma 3**, $F_0(z)$ then either needs to be strictly monotonically increasing or strictly monotonically decreasing. If it is increasing, it follows for any $z \in (0, 1 - p_0]$ that $V_{\boldsymbol{\kappa}}(\mathbf{p} + z\mathbf{e}_0, \mathbf{q}) > V_{\boldsymbol{\kappa}}(\mathbf{p}, \mathbf{q})$. For $p_0 < 1$, this yields a contradiction because X was assumed to be unable to locally improve its objective function. A similar contradiction arises if $F_0(z)$ is strictly decreasing and $p_0 > 0$. In that case, $V_{\boldsymbol{\kappa}}(\mathbf{p} + z\mathbf{e}_0, \mathbf{q}) > V_{\boldsymbol{\kappa}}(\mathbf{p}, \mathbf{q})$ for all $z \in [-p_0, 0)$. An analogous proof shows the result for all other marginal functions $F_{xy}(z)$. □

Lemma 5

Suppose player X's current objective function is $V_{\boldsymbol{\kappa}}$ for some given $\boldsymbol{\kappa} = (\kappa_X, \kappa_Y)$, and consider players with strategies $\mathbf{p} \in (0,1)^5$ and $\mathbf{q} \in [0,1]^5$ such that $\mathbf{p} \notin BR_{V_{\boldsymbol{\kappa}}}(\mathbf{q})$. Moreover, suppose player X is unable to locally improve its objective function. Then, there is a $d > 0$ such that $V_{\boldsymbol{\kappa}}(\mathbf{p}', \mathbf{q}) = V_{\boldsymbol{\kappa}}(\mathbf{p}, \mathbf{q})$ for all $\mathbf{p}'$ with $\|\mathbf{p}' - \mathbf{p}\| < d$.

Proof. Because player X is unable to increase fairness and $\mathbf{p} \in (0,1)^5$, we can find a $d > 0$ such that all $\mathbf{p}'$ with $\|\mathbf{p}' - \mathbf{p}\| < d$ satisfy $\mathbf{p}' \in (0,1)^5$ and $V_{\boldsymbol{\kappa}}(\mathbf{p}', \mathbf{q}) \leqslant V_{\boldsymbol{\kappa}}(\mathbf{p}, \mathbf{q})$. Now assume that there is at least one such $\mathbf{p}'$ for which the inequality is strict, $V_{\boldsymbol{\kappa}}(\mathbf{p}', \mathbf{q}) < V_{\boldsymbol{\kappa}}(\mathbf{p}, \mathbf{q})$. Consider the sequence of strategies,

$$\mathbf{p}^0 := (p_0, p_{CC}, p_{CD}, p_{DC}, p_{DD})\,; \tag{S15a}$$

$$\mathbf{p}^1 := (p_0', p_{CC}, p_{CD}, p_{DC}, p_{DD})\,; \tag{S15b}$$

$$\mathbf{p}^2 := (p_0', p_{CC}', p_{CD}, p_{DC}, p_{DD})\,; \tag{S15c}$$

$$\mathbf{p}^3 := (p_0', p_{CC}', p_{CD}', p_{DC}, p_{DD})\,; \tag{S15d}$$

$$\mathbf{p}^4 := (p_0', p_{CC}', p_{CD}', p_{DC}', p_{DD})\,; \tag{S15e}$$

$$\mathbf{p}^5 := (p_0', p_{CC}', p_{CD}', p_{DC}', p_{DD}')\,. \tag{S15f}$$

In particular, $\mathbf{p}^0 = \mathbf{p}$, $\mathbf{p}^5 = \mathbf{p}'$, and in each step we only vary one coordinate. Moreover, $\|\mathbf{p}^i - \mathbf{p}\| \leqslant \|\mathbf{p}' - \mathbf{p}\| < d$ for all i, and therefore $V_{\boldsymbol{\kappa}}(\mathbf{p}^i, \mathbf{q}) \leqslant V_{\boldsymbol{\kappa}}(\mathbf{p}, \mathbf{q})$ for all i. Let j be

the largest number such that $V_{\boldsymbol{\kappa}}\left(\mathbf{p}^{j},\mathbf{q}\right) = V_{\boldsymbol{\kappa}}\left(\mathbf{p},\mathbf{q}\right)$. By construction, we must have $j < 5$. Since $\mathbf{p}^{j} \in (0,1)^5$ and player X also needs to be unable to locally increase fairness around the new point $\left(\mathbf{p}^{j},\mathbf{q}\right)$, it follows from **Lemma 4** that any marginal function centered at the new point $\left(\mathbf{p}^{j},\mathbf{q}\right)$ has to be constant. Since $\mathbf{p}^{j+1}$ and $\mathbf{p}^{j}$ only differ in one coordinate, it follows that $V_{\boldsymbol{\kappa}}\left(\mathbf{p}^{j+1},\mathbf{q}\right) = V_{\boldsymbol{\kappa}}\left(\mathbf{p}^{j},\mathbf{q}\right) = V_{\boldsymbol{\kappa}}\left(\mathbf{p},\mathbf{q}\right)$, which contradicts the definition of j as the largest number for which equality holds. □

S2.2.4 Local improvements in the interior of the strategy space

We can now show that, for generic strategies in the interior of the state space, player X can always locally increase her objective function $V_{\boldsymbol{\kappa}}$ (unless X uses a best response already). Note that the following result does not make any assumptions on the exact payoffs of the game:

Proposition 1 (Existence of local improvements in the interior of the strategy space)
Consider a player X with weighted payoff objectives $V_{\boldsymbol{\kappa}}$ for some given $\boldsymbol{\kappa} = (\kappa_X,\kappa_Y)$, and suppose the players' strategies are $\mathbf{p} \in (0,1)^5$ and $\mathbf{q} \in [0,1]^5$ such that $\mathbf{p} \notin BR_{V_{\boldsymbol{\kappa}}}(\mathbf{q})$. Then player X can locally improve his or her objective function.

Proof. Given the co-player's strategy, let $\mathbf{p}^* \in \mathrm{BR}_{V_{\boldsymbol{\kappa}}}(\mathbf{q})$ be an arbitrary best response. Then, $V_{\boldsymbol{\kappa}}(\mathbf{p},\mathbf{q}) < V_{\boldsymbol{\kappa}}(\mathbf{p}^*,\mathbf{q})$ since $\mathbf{p} \notin \mathrm{BR}_{V_{\boldsymbol{\kappa}}}(\mathbf{q})$. We now define a function $h : [0,1] \to \mathbb{R}$ by

$$h(z) := V_{\boldsymbol{\kappa}}\big((1-z)\,\mathbf{p} + z\mathbf{p}^*,\mathbf{q}\big) - V_{\boldsymbol{\kappa}}(\mathbf{p},\mathbf{q}). \tag{S16}$$

By Equation **S14**, it follows that $h(z)$ is a rational function of z. Suppose to the contrary that player X cannot locally improve $V_{\boldsymbol{\kappa}}$. By **Lemma 5**, $h(z)$ must be identically zero for all z sufficiently small. But a rational function has either finitely many zeros or is identically zero, so $h(z) = 0$ for all $z \in [0,1]$. However, by Equation **S16**, $h(1) = V_{\boldsymbol{\kappa}}\left(\mathbf{p}^*,\mathbf{q}\right) - V_{\boldsymbol{\kappa}}(\mathbf{p},\mathbf{q}) > 0$. □

As a result, if both players adopt strategies in the interior of the strategy space, the restriction that players need to adapt locally does not introduce any new equilibria. If both players are unable to locally improve their objective function, then their respective strategies need to be (global) best replies to each other (relative to their respective objective functions).

Next, we apply **Proposition 1** to discuss the special cases that player X has objectives based on selfishness, efficiency, or fairness:

Proposition 2 (Existence of local improvements for some important objective functions)
Consider a player X with objective function V in a symmetric base game with continuation probability $\lambda > 0$ and payoffs $\mathbf{u}_X$, $\mathbf{u}_Y$, such that $R \geqslant P$. Moreover, suppose the player's strategies are $\mathbf{p} \in (0,1)^5$ and $\mathbf{q} \in [0,1]^5$, and that player X cannot locally improve its objective function.

1. *If X has selfish objectives, meaning $V(\mathbf{p},\mathbf{q}) = \pi_X(\mathbf{p},\mathbf{q})$, then $\mathbf{q}$ must be an equalizer strategy. That is, for $\lambda < 1$ the entries of $\mathbf{q}$ need to satisfy the equations (37, 41)*

$$\lambda(R-P)\,q_{CD} = \lambda(T-P)\,q_{CC} - (T-R)(1+\lambda q_{DD})\,; \tag{S17a}$$

$$\lambda(R-P)\,q_{DC} = (P-S)(1-\lambda q_{CC}) + (R-S)\,\lambda q_{DD}. \tag{S17b}$$

For $\lambda = 1$, the strategy $\mathbf{q}$ additionally needs to be different from the degenerate strategy Repeat *defined by $q_{CC} = q_{CD} = 1$ and $q_{DC} = q_{DD} = 0$.*

2. *If X has efficiency objectives, i.e. $V(\mathbf{p},\mathbf{q}) = \pi_X(\mathbf{p},\mathbf{q}) + \pi_Y(\mathbf{p},\mathbf{q})$, then the game needs to be constant-sum ($2R = 2P = S + T$). In that case, any $\mathbf{p}$ and $\mathbf{q}$ satisfy the assumptions.*

3. *If X has fairness objectives, i.e. $V(\mathbf{p},\mathbf{q}) = -|\pi_X(\mathbf{p},\mathbf{q}) - \pi_Y(\mathbf{p},\mathbf{q})|$, then the payoffs need to be equal ($\pi_X(\mathbf{p},\mathbf{q}) = \pi_Y(\mathbf{p},\mathbf{q})$).*

Proof. 1. Because player X is unable to locally improve its objective function, it follows by an argument similar to the proof of **Proposition 1** that $V(\mathbf{p},\mathbf{q}) = V(\mathbf{p}^*,\mathbf{q})$ for all alternative strategies $\mathbf{p}^*$. In other words, player X's payoff is independent of its strategy. The strategies $\mathbf{q}$ that have this property have been called *equalizers* (44). For discounted games, the equalizers are exactly the strategies satisfying Equation **S17** (37, 41). For games without discounting, it is well-known that *Repeat* needs to be excluded explicitly (34).

2. By **Remark 1**, players with efficiency objectives can interpreted as players with selfish objective in a game with modified one-shot payoffs $\mathbf{u}_E := \mathbf{u}_X + \mathbf{u}_Y = (2R, S+T, S+T, 2P)$. We can thus use Equation **S17** to obtain a necessary condition on player Y's strategy:

$$2\lambda(R-P)\,q_{CD} = \lambda(S+T-2P)\,q_{CC} - (S+T-2R)(1+\lambda q_{DD})\,; \qquad \text{(S18a)}$$

$$2\lambda(R-P)\,q_{DC} = (2P-T-S)(1-\lambda q_{CC}) + (2R-T-S)\,\lambda q_{DD}. \qquad \text{(S18b)}$$

In the special case that $R = P$, the sum of these two equations simplifies to $0 = 2R - T - S$, and therefore $2R = 2P = S + T$. In the other case, $R > P$, taking the difference between these two equations gives $\lambda(q_{CD} - q_{DC}) = 1$. This equation cannot be satisfied when $\lambda < 1$, and when $\lambda = 1$ it requires $q_{CD} = 1$ and $q_{DC} = 0$. Plugging these values into Equation **S18**, we find that $q_{CC} = 1$ and $q_{DD} = 0$, so $\mathbf{q}$ must be the strategy *Repeat*, which is explicitly excluded.

3. Assume to the contrary that payoffs are unequal. Without loss of generality, we may assume that $\pi_X(\mathbf{p},\mathbf{q}) < \pi_Y(\mathbf{p},\mathbf{q})$. It follows that locally, player X's objective function has the form $V_{\boldsymbol{\kappa}}$ with $\boldsymbol{\kappa} = (1,-1)$. Since $V_{\boldsymbol{\kappa}}(\mathbf{p},\mathbf{q}) < 0 = V_{\boldsymbol{\kappa}}(\mathbf{q},\mathbf{q})$, we have $\mathbf{p} \notin \mathrm{BR}_{V_{\boldsymbol{\kappa}}}(\mathbf{q})$. By **Proposition 1**, it then follows that player X can locally improve its objective function. That is, for any distance $d > 0$, one can find a strategy $\mathbf{p}_d$ with $\|\mathbf{p}_d - \mathbf{p}\| < d$ such that $V_{\boldsymbol{\kappa}}(\mathbf{p}_d,\mathbf{q}) > V_{\boldsymbol{\kappa}}(\mathbf{p},\mathbf{q})$. Moreover, because $V_{\boldsymbol{\kappa}}$ is a continuous function of its inputs, $V_{\boldsymbol{\kappa}}(\mathbf{p}_d,\mathbf{q}) < 0$ for all d that are sufficiently small. For any such d, we have $-|V_{\boldsymbol{\kappa}}(\mathbf{p},\mathbf{q})| < -|V_{\boldsymbol{\kappa}}(\mathbf{p}_d,\mathbf{q})|$. Thus, local improvements are possible for all d, a contradiction. □

S2.2.5 Local improvements on the boundary of the strategy space

In the following, we briefly discuss under which condition player X is guaranteed to find a local improvement when its current strategy is on the boundary, $\mathbf{p} \in \mathrm{bd}\left([0,1]^5\right)$.

Obviously, any local improvement is impossible when both players already use a best response to each other (given their objectives). However, even in the standard case of two players

with selfish objectives, very little is known about the corresponding set of all Nash equilibria among the memory-one strategies. Most available results address games without discounting, $\lambda = 1$. For such games, Akin (38) described all Nash equilibria profiles $(\mathbf{p}, \mathbf{q})$ that sustain full cooperation in the prisoner's dilemma. Stewart and Plotkin (36) describe all symmetric Nash equilibria $(\mathbf{p}, \mathbf{p})$ among the memory-one strategies for all 2×2 games. For generic cases (with $R \neq P$ and $T \neq S$) they find that in addition to the equalizer strategies of the form Equation **S17**, there are three more classes of equilibrium strategies $\mathbf{p}$:

1. **Strategies that result in mutual cooperation** are a Nash equilibrium if and only if

$$p_{CC} = 1; \tag{S19a}$$
$$(T - R)\, p_{DC} \leqslant (R - S)\,(1 - p_{CD})\,; \tag{S19b}$$
$$(T - R)\, p_{DD} \leqslant (R - P)\,(1 - p_{CD})\,. \tag{S19c}$$

2. **Strategies that result in alternating cooperation** are a Nash equilibrium if and only if

$$p_{CD} = 0; \tag{S20a}$$
$$p_{DC} = 1; \tag{S20b}$$
$$(T - S)\, p_{CC} \leqslant 2\,(T - R)\,; \tag{S20c}$$
$$(T - S)\, p_{DD} \leqslant (S + T - 2P)\,. \tag{S20d}$$

3. **Strategies that result in mutual defection** are a Nash equilibrium if and only if

$$p_{DD} = 0; \tag{S21a}$$
$$(R - P)\, p_{DC} \leqslant (P - S)\,(1 - p_{CC})\,; \tag{S21b}$$
$$(T - P)\, p_{DC} \leqslant (P - S)\,(1 - p_{CD})\,. \tag{S21c}$$

In the following, we ask whether these three qualitative outcomes are still feasible if (at least) one of the players is motivated by efficiency:

Proposition 3
Consider a player X whose objective is to maximize efficiency, $V(\mathbf{p}, \mathbf{q}) = \pi_X(\mathbf{p}, \mathbf{q}) + \pi_Y(\mathbf{p}, \mathbf{q})$ in a symmetric base game with $R > P$ and continuation probability $\lambda > 0$.

1. *Suppose that $R \geqslant (S + T)/2$ and that both players mutually cooperate, giving $\pi_X = \pi_Y = R$. Then, player X is unable to locally (or globally) improve its objective function.*

2. *Suppose $R < (S + T)/2$, and both players cooperate in an alternating fashion, giving $\pi_X = \pi_Y = (S + T)/2$. Then, player X is unable to locally (or globally) improve its objective function.*

3. *Suppose both players mutually defect, giving $\pi_X = \pi_Y = P$. Then there are strategies $\mathbf{q}$ for player Y such that player X is unable to locally (or globally) improve its objective function if and only if $(S + T)/2 \leqslant P$.*

Proof. 1. By assumption, the current efficiency is $\pi_X(\mathbf{p},\mathbf{q})+\pi_Y(\mathbf{p},\mathbf{q})=2R$. After any deviation of player X to some other strategy $\mathbf{p}^*$, this efficiency takes the form

$$\pi_X(\mathbf{p}^*,\mathbf{q})+\pi_Y(\mathbf{p}^*,\mathbf{q})=a_1(2R)+a_2(S+T)+a_3(2P), \tag{S22}$$

where $a_1,a_2,a_3\geqslant 0$ and $a_1+a_2+a_3=1$. Because $R\geqslant(S+T)/2$ and $R>P$, this efficiency is at most $2R$.

2. By assumption, the current efficiency is $\pi_X(\mathbf{p},\mathbf{q})+\pi_Y(\mathbf{p},\mathbf{q})=S+T$. After a deviation, the efficiency again takes the form of Equation **S22**. Because now $(S+T)/2\geqslant R\geqslant P$, again it follows that this efficiency is at most $S+T$.

3. First, assume that $(S+T)/2>P$, and suppose player X deviates by choosing a strategy $\mathbf{p}^*=\mathbf{p}+\varepsilon\mathbf{e}_{DD}$, with $\varepsilon>0$ being arbitrarily small. Again, the efficiency after the deviation takes the form of Equation **S22**, with either a_1 or a_2 strictly positive. Because $R>P$ and $(S+T)/2>P$, this efficiency is larger than the current efficiency of $2P$. Hence, player X is able to locally improve its objective function.

   For the other direction, assume now that $(S+T)/2\leqslant P$ and that player Y adopts the strategy ALLD. Then any deviation $\mathbf{p}^*$ by player X leads to an efficiency of the form

$$\pi_X(\mathbf{p}^*,\mathbf{q})+\pi_Y(\mathbf{p}^*,\mathbf{q})=a(S+T)+(1-a)(2P), \tag{S23}$$

   which is at most $2P$. Thus player X is unable to locally (or globally) improve its objective function. □

In particular, this result implies that in donation games, players cannot settle at mutual defection if at least one of them applies *FMTL*. On the other hand, in the special case of a prisoner's dilemma with $(S+T)/2\leqslant P$, mutual defection can be stable even if both players value efficiency. This effect arises because in our framework (and in evolutionary game theory more generally), learners adapt their strategies independently from each other. In games in which one player alone is unable to escape from inefficient strategy profiles, players may thus end up in a rest point that they both wish to avoid.

S3 Evolutionary dynamics of learning rules

Here, we briefly outline several qualitative observations about the evolutionary dynamics of learning rules. Recall that the dynamics we consider depend on mean payoffs only. Suppose that $\mathcal{R}$ and $\mathcal{S}$ are learning rules. For $\mathcal{X},\mathcal{Y}\in\{\mathcal{R},\mathcal{S}\}$, we let $a_{\mathcal{X}\mathcal{Y}}(n)$ denote the mean payoff to $\mathcal{X}$ against $\mathcal{Y}$ after n learning steps, averaged over initial conditions and learning trajectories.

These mean payoffs motivate a qualitative comparison among learning rules based on how they perform against one another, on average. We consider three broad conditions:

- $\mathcal{R}$ *exploits* $\mathcal{S}$ after n steps if $a_{\mathcal{R}\mathcal{S}}(n)>a_{\mathcal{S}\mathcal{R}}(n)$;
- $\mathcal{R}$ is *fair relative to* $\mathcal{S}$ after n steps if $a_{\mathcal{R}\mathcal{S}}(n)=a_{\mathcal{S}\mathcal{R}}(n)$;

- $\mathcal{R}$ is *exploited by* $\mathcal{S}$ after n steps if $a_{\mathcal{RS}}(n) < a_{\mathcal{SR}}(n)$.

Note that each of these conditions disregards the performance of a learning rule against itself, i.e. $a_{\mathcal{RR}}(n)$ and $a_{\mathcal{SS}}(n)$. As we will see below, these conditions do not alone allow one to conclude anything about evolutionary stability, but they do provide reasonable classifications for a first comparison of two learning rules.

Example 1. In Figure S9b, team-based learning (maximizing $\pi_X + \pi_Y$) is exploited by selfish learning after sufficiently many learning steps. In all of our examples, *FMTL* is fair relative to selfish learning after long enough learning horizons, but it can be exploited by selfish learning on shorter timescales (e.g. Figure 4a). However, as the example of Figure 4a shows, *FMTL* can still be evolutionary stable relative to selfish learning even when it is being exploited, due to the superior performance of *FMTL* against itself relative to selfish learning against itself. By symmetry, any learning rule is fair relative to itself.

Example 2. Selfish learning, i.e. caring about one's own payoff only, can actually still be exploited by some learning rules. To see why, we can formulate a learning rule that takes advantage of selfish learners. Suppose that $\chi > 1$. In the donation game with benefit b and cost c, consider the following learning rule: if $\pi_X \geqslant \chi\pi_Y$, then maximize $\pi_X + \pi_Y$; otherwise, minimize $|\pi_X - \chi\pi_Y|$. When $b = 2$, $c = 1$, $\chi = 2$, and this learning rule is paired with a selfish learner, the mean payoffs to the two learners over 10^5 runs are 1.1788 and 0.5894, respectively. We do not wish to dwell on this particular learning rule; rather, this example is intended simply to illustrate that even selfish learners can be exploited by learners with sufficiently coercive preferences.

The following result says that if $\mathcal{S}$ does not exploit an opposing learning rule, $\mathcal{R}$, then $\mathcal{S}$ cannot be evolutionary stable relative to $\mathcal{R}$ whenever $\mathcal{R}$ is optimal against itself. (To simplify the notation, we suppress the number of learning steps, n.)

Proposition 4
Suppose that $\mathcal{R}$ either exploits $\mathcal{S}$ or is fair relative to $\mathcal{S}$, and suppose furthermore that $a_{\mathcal{RR}}$ is maximal (which can be determined by looking at the feasible region for the repeated game). Then, either (i) $\mathcal{R}$ is neutral relative to $\mathcal{S}$ or (ii) $\mathcal{R}$ is evolutionarily stable relative to $\mathcal{S}$.

Proof. Since $\mathcal{R}$ exploits $\mathcal{S}$ or is fair relative to $\mathcal{S}$, we have $a_{\mathcal{RS}} \geqslant a_{\mathcal{SR}}$. Moreover, since $a_{\mathcal{RR}}$ is maximal and the feasible region is convex, we must have $a_{\mathcal{RR}} \geqslant a_{\mathcal{SR}}$. If $a_{\mathcal{RR}} > a_{\mathcal{SR}}$, then $\mathcal{R}$ is automatically evolutionarily stable relative to $\mathcal{S}$. But if $a_{\mathcal{RR}} = a_{\mathcal{SR}}$, then $a_{\mathcal{RS}} \geqslant a_{\mathcal{SR}} = a_{\mathcal{RR}} \geqslant a_{\mathcal{SS}}$. If these inequalities are all equalities, then $\mathcal{R}$ is neutral relative to $\mathcal{S}$; otherwise, $\mathcal{R}$ satisfies Maynard Smith's second condition (86) and is thus evolutionarily stable relative to $\mathcal{S}$. □

For example, in the hero game, we know that *FMTL* is both fair and optimal against itself after sufficiently many learning steps (Figure S8b). Thus, selfish learning cannot be evolutionary stable relative to *FMTL*. In this case, since selfish learning is also optimal against itself, *FMTL* is neutral relative to selfish learning. Of course, one can always deduce evolutionary stability by looking at the mean payoffs $a_{\mathcal{XY}}$ themselves. **Proposition 4** simply says that a learner who performs optimally against itself is *at least* neutral against any nonexploitative opponent.

S4 Beyond introspection: imitation as a form of learning

The "evolutionary" aspect of our model enters in describing competition between learning rules (the "supergame" dynamics), not in the implementation of learning rules themselves. Rather, the learning rules we consider are implemented between two players (a trivial case of a population) using introspection. Each player samples a new strategy nearby and then determines whether it wants to adopt this strategy or retain the old one. The difference between a selfish learning and *FMTL* amounts to the objective(s) used in determining whether to adopt the new strategy.

Introspection is a natural choice for learners because each learner knows their own strategy and can thus sample something nearby to test against an opponent. Whether the player accepts this strategy or not depends on observed payoffs; a player does not specifically need to know the strategy used by the opponent. In contrast, imitation dynamics require that a player can copy an opponent's strategy, which may or may not be a reasonable assumption. In classical models involving a small number of strategic types, a player simply needs to see the action taken by the opponent and then copy that action (87). But repeated games involve more complicated conditional behavior, in general, and a player might not be able to faithfully infer such a strategy by interacting with an opponent. This observation is not to discount the value of imitation but rather to say that it likely entails a degree of error or mutation in strategy transmission.

Nonetheless, we can study what happens when a player's objectives are evaluated through imitation rather than introspection. Suppose that $s \in [0,1]$. If X imitates the strategy $\mathbf{q}$ of Y, then we assume that the ith coordinate of X's strategy is $\min\left\{\max\left\{q_i+z_i,0\right\},1\right\}$, where z_i is uniformly distributed on $[-s,s]$, and z_i is independent of z_j for $j\neq i$. This mutation procedure is identical to the one used for introspection dynamics in the main text, although here we use $s=10^{-2}$; there are always errors in copying strategies, but these errors are not too large.

The baseline for comparison here, analogous to a selfish learner in the main text, is a selfish imitator. Suppose that X is a selfish imitator using $\mathbf{p}$, and let Y be a model player who uses $\mathbf{q}$. X chooses another individual, Z, using $\mathbf{r}$, and asks how X fares against Z relative to how Y fares against Z. Since our focus is on symmetric games, we can simplify notation and write $\pi\left(\mathbf{p},\mathbf{r}\right)$ for $\pi_X\left(\mathbf{p},\mathbf{r}\right)$ and $\pi\left(\mathbf{r},\mathbf{p}\right)$ for $\pi_Z\left(\mathbf{p},\mathbf{r}\right)$. X then copies the strategy of Y with probability

$$\frac{1}{1+e^{-\delta\left(\pi(\mathbf{q},\mathbf{r})-\pi(\mathbf{p},\mathbf{r})\right)}}, \tag{S24}$$

where $\delta\geqslant 0$ represents the intensity of selection. This is known as Fermi updating (88, 89).

The analogue of *FMTL* first chooses an objective function to optimize, based on the current value of $\left|\pi\left(\mathbf{p},\mathbf{r}\right)-\pi\left(\mathbf{r},\mathbf{p}\right)\right|$, exactly as prescribed in the main text. The key distinction between this implementation of *FMTL* and the one in the main text lies in the next step. If efficiency is the objective, then X imitates Y based on whether Y and Z have a larger total payoff than X and Z. If the objective is fairness, then X imitates Y based on whether Y's payoff is closer to Z's than X's is to Z's. More specifically, the probability that X imitates Y's strategy, relative to Z, is

$$\begin{cases} \dfrac{1}{1+e^{-\delta\left(\left(\pi(\mathbf{q},\mathbf{r})+\pi(\mathbf{r},\mathbf{q})\right)-\left(\pi(\mathbf{p},\mathbf{r})+\pi(\mathbf{r},\mathbf{p})\right)\right)}} & \text{with probability } \omega\left(\mathbf{p},\mathbf{r}\right), \\ \\ \dfrac{1}{1+e^{-\delta\left(\left|\pi(\mathbf{p},\mathbf{r})-\pi(\mathbf{r},\mathbf{p})\right|-\left|\pi(\mathbf{q},\mathbf{r})-\pi(\mathbf{r},\mathbf{q})\right|\right)}} & \text{with probability } 1-\omega\left(\mathbf{p},\mathbf{r}\right), \end{cases} \tag{S25}$$

where ω is the function of Equation **5** (or Equation **6**) of the main text. For both selfish and *FMTL* imitators, we use a selection intensity of $\delta = 1.0$.

Using the donation game as an example, our findings in Figure 2 and Figure 4**a** say that *FMTL* improves the outcomes for all learners and cannot be exploited by selfish learners. To explore whether this behavior is preserved under imitation dynamics, we consider three settings: *(i)* populations consisting of only selfish imitators; *(ii)* populations consisting of a mixture of selfish imitators and *FMTL* imitators; and *(iii)* populations consisting of only *FMTL* imitators. In unstructured populations of finite size N, there are $N - 1$ possibilities for option *(ii)*, one for each number of *FMTL* imitators between 1 and $N - 1$. Figure S11 illustrates imitation dynamics in a population of size $N = 100$ when **a** all imitators are selfish, **b** there are as many *FMTL* imitators as selfish imitators, and **c** all imitators use *FMTL*. Qualitatively, we find the same outcomes reported in the main text: selfish imitators lead to relatively poor outcomes overall, but the presence of *FMTL* leads to substantial improvements while avoiding exploitation. Figure S12 demonstrates this trend beyond populations with an equal number of each kind of imitator. Worthy of note is the fact that a selfish imitator would always be better off if they switched to *FMTL*.

The payoffs of selfish imitators and *FMTL* imitators in Figure S11**b** are highly correlated. However, this is not necessarily attributed entirely to the fact that *FMTL* emphasizes fairness. Because changes in strategies are based on imitation, a player who imitates based on *FMTL* can copy a strategy that was shaped by selfish objectives and vice versa. What was learned by one player can thus be directly transferred to another player, even when the two players have different preferences over how to imitate. In this sense, imitation dynamics result in blended learning within a population, which partly contributes to the correlations between payoffs across types. Actually, even team-based imitation alone can improve outcomes for all, relative to selfish imitation, which is a finding that does not hold in the introspective setting (Figure S9**b**).

Beyond an individual's ability to infer and imitate a complicated strategy, other issues arise when considering social preferences in classical models, especially when strategies are inherited instead of imitated. Under birth-death dynamics, individuals reproduce and pass down their strategies for a repeated game, subject to mutation. More successful strategies tend to produce more offspring, so these dynamics may be thought of as encoding a kind of selfish learning. However, unlike in imitation dynamics, individuals do not necessarily have the agency to choose their reproductive rates based on preferences. Just because an individual values efficiency or fairness does not mean that their birth rate increases as those objectives get closer to their optima.

In summary, *FMTL* is most natural when implemented in settings for which social preferences can be effectively expressed, including introspection and imitation. Our main focus is on introspection-based learning in pairs, which is quite different from the population-based model of imitation presented here. However, the latter illustrates an important point: the key beneficial properties of *FMTL* are not due to introspection but rather to the specific preferences such learners use when making choices.

S5 Asymmetric games

S5.1 Weakly-asymmetric games

Our focus in this study is on symmetric games, but it is natural to ask how the results extend to settings with asymmetric interactions. Consider a general two-action bimatrix game,

$$\begin{array}{c} \\ U \\ D \end{array} \begin{array}{c} \begin{array}{cc} L & R \end{array} \\ \left(\begin{array}{cc} a_1, b_1 & a_2, b_2 \\ a_3, b_3 & a_4, b_4 \end{array} \right) \end{array} . \tag{S26}$$

Here, X is the row player and Y is the column player. The respective action spaces are $S_X = \{U, D\}$ ("up" and "down") for X and $S_Y = \{L, R\}$ ("left" and "right") for Y, with a_i denoting X's payoff and b_i denoting Y's payoff. If $A := \begin{pmatrix} a_1 & a_2 \\ a_3 & a_4 \end{pmatrix}$ and $B := \begin{pmatrix} b_1 & b_2 \\ b_3 & b_4 \end{pmatrix}$, then this game is symmetric if $S_X = S_Y$ and $B^\intercal = A$. Note that this definition is based on a comparison of the exact values in A and B, and it implicitly identifies U with L and D with R. We will consider a weaker version of this definition, in which we use relative rankings of the values in each matrix. More specifically, let $\mathrm{rank}\,(a_i, A) = k$ mean that a_i is the kth largest value in A (ties are allowed when two values are equal). Now, consider the two ranked matrices $A^{\mathrm{ranked}} := \begin{pmatrix} \mathrm{rank}\,(a_1, A) & \mathrm{rank}\,(a_2, A) \\ \mathrm{rank}\,(a_3, A) & \mathrm{rank}\,(a_4, A) \end{pmatrix}$ and $B^{\mathrm{ranked}} := \begin{pmatrix} \mathrm{rank}\,(b_1, B) & \mathrm{rank}\,(b_2, B) \\ \mathrm{rank}\,(b_3, B) & \mathrm{rank}\,(b_4, B) \end{pmatrix}$. We say that this game is *weakly-asymmetric* if $\left(B^{\mathrm{ranked}}\right)^\intercal = A^{\mathrm{ranked}}$. More generally, we say that:

Definition 3 (Weakly-asymmetric game)
An asymmetric N-player game with action spaces $S_1, \dots, S_N$ and utility function $u : \prod_{i=1}^N S_i \to \mathbb{R}^N$ is weakly asymmetric *if there exists a symmetric game with action space S and utility function $u' : S^N \to \mathbb{R}^N$, together with bijections $\{\phi_i : S_i \to S\}_{i=1}^N$ such that for all $i = 1, \dots, N$ and $s, s' \in \prod_{i=1}^N S_i$, $sgn\,(u_i\,(s) - u_i\,(s')) = sgn\,(u'_i\,(\phi\,(s)) - u'_i\,(\phi\,(s')))$, where $\phi = \phi_1 \times \cdots \times \phi_N$ and $sgn\,(x)$ is -1 if $x < 0$, 0 if $x = 0$, and 1 if $x > 0$. In other words, for all $s, s' \in \prod_{i=1}^N S_i$,*

$$u_i\,(s) \geqslant u_i\,(s') \iff u'_i\,(\phi\,(s)) \geqslant u'_i\,(\phi\,(s'))\,, \tag{S27}$$

with equality on the left-hand side if and only if there is equality on the right-hand side.

Example 3. Consider the two-player "battle of the sexes" game (90), with utility u given by

$$\begin{array}{c} \\ F \\ B \end{array} \begin{array}{c} \begin{array}{cc} F & B \end{array} \\ \left(\begin{array}{cc} 3, 2 & 1, 1 \\ 0, 0 & 2, 3 \end{array} \right) \end{array} . \tag{S28}$$

The interpretation of this game is that two individuals (row and column) have different preferences and choose between two options: attend a fight (F) and attend a ballet (B). The row player prefers seeing a fight, while the column player prefers seeing a ballet. They prefer, however, to be together rather than to be apart, which gives the payoff ranking depicted in the matrix.

Let $\phi_1, \phi_2 : \{F, B\} \to \{C, D\}$ be the bijections with $\phi_1(F) = C$ and $\phi_2(F) = D$. With these correspondences on the strategy spaces, this battle of the sexes game maps to the symmetric "hero" game described in the main text, with utility function u' given by the payoff matrix

$$\begin{array}{cc} & \begin{array}{cc} C & D \end{array} \\ \begin{array}{c} C \\ D \end{array} & \begin{pmatrix} 1 & 3 \\ 2 & 0 \end{pmatrix} \end{array} . \tag{S29}$$

This mapping preserves the value of $u_i(s) - u_i(s')$, so the battle of the sexes game is weakly asymmetric. However, note that there is no such mapping to a symmetric game if $\phi_1(F) = \phi_2(F) = C$. The reason is that if we have a symmetric matrix game with parameters R, S, T, and P, then the fact that $u_1(F,F) = 3 > 2 = u_1(B,B)$ requires that $R > P$. At the same time, the fact that $u_2(F,F) = 2 < 3 = u_2(B,B)$ requires that $R < P$, and we cannot have both of these inequalities. Thus, in mapping the battle of the sexes game to a symmetric game, it was necessary to map one player's choice of F to C and the other player's choice of F to D.

This property of the battle of the sexes game partially motivates the definition of weakly-asymmetric games. The two players view the same option, B, differently. So, when considering the action F for one player, the comparable choice for the second player is not necessarily the same nominal action. Rather, it is the action that, relative to player two, is most comparable to the action F, relative to player one. In this case, we can think of C as meaning "choose one's own preference" and D as "choose the partner's preference." This aspect of the battle of the sexes game will be important for understanding fairness because we will need to talk about what happens when two players swap actions (or imitate) one another; and if player one chooses F, then when player two imitates this action, she actually chooses B rather than F.

Another, slightly simpler example is the asymmetric donation game, in which player X can pay a cost c to donate b_X and player Y can pay c to donate b_Y. Taking such an action is considered "cooperation" for each player, whereas "defection" for either X or Y amounts to paying nothing and donating nothing. In this case, the action C of X corresponds to the action C of Y, so we do not have to deal with the same subtlety that arises in the battle of the sexes game. In fact, we will use this game as our main illustration. However, before describing results for the asymmetric donation game, we note that the approach outlined above assumes that players have comparable actions, so that they can swap strategies in the first place. Within the space of weakly-asymmetric games, there must exist a correspondence $\phi_Y^{-1} \circ \phi_X : S_X \to S_Y$, so we know that there is at least one way of relating the strategies of X to those of Y. Generically, this correspondence is unique:

Proposition 5

In a weakly-asymmetric bimatrix game of the form depicted in Equation **S26***, any two bijections ϕ_X and ϕ_Y satisfying the conditions in Definition 3 give rise to a unique bijection between actions, $\phi_Y^{-1} \circ \phi_X : \{U, D\} \to \{L, R\}$, unless $a_1 = a_4$, $a_2 = a_3$, $b_1 = b_4$, and $b_2 = b_3$.*

Proof. By relabeling the strategies in Equation **S26** if necessary, we may assume that the bijections ϕ_X and ϕ_Y satisfying Definition 3 are such that the bijection $\phi_Y^{-1} \circ \phi_X : \{U, D\} \to \{L, R\}$ sends U to L. By the definition of a weakly-asymmetric game, we then have the equation

$$\begin{pmatrix} \mathrm{rank}\,(a_1, A) & \mathrm{rank}\,(a_2, A) \\ \mathrm{rank}\,(a_3, A) & \mathrm{rank}\,(a_4, A) \end{pmatrix} = \begin{pmatrix} \mathrm{rank}\,(b_1, B) & \mathrm{rank}\,(b_3, B) \\ \mathrm{rank}\,(b_2, B) & \mathrm{rank}\,(b_4, B) \end{pmatrix} . \tag{S30}$$

If there were another bijection $\widetilde{\phi}_Y^{-1} \circ \widetilde{\phi}_X : \{U, D\} \to \{L, R\}$ sending U to R instead, such that $\widetilde{\phi}_X$ and $\widetilde{\phi}_Y$ also satisfy Definition 3, then by swapping the columns of Equation **S26** we have

$$\begin{pmatrix} \mathrm{rank}\,(a_2, A) & \mathrm{rank}\,(a_1, A) \\ \mathrm{rank}\,(a_4, A) & \mathrm{rank}\,(a_3, A) \end{pmatrix} = \begin{pmatrix} \mathrm{rank}\,(b_2, B) & \mathrm{rank}\,(b_4, B) \\ \mathrm{rank}\,(b_1, B) & \mathrm{rank}\,(b_3, B) \end{pmatrix}. \tag{S31}$$

Combining Equations **S30–S31**, we see that $a_1 = a_4$, $a_2 = a_3$, $b_1 = b_4$, and $b_2 = b_3$. □

S5.2 *FMTL* for weakly-asymmetric games

Clearly, not all asymmetric games are weakly asymmetric because players can either have incomparable actions or even a different number of actions to choose from. An analysis of "fairness" in general asymmetric games is complicated and beyond the scope of this paper. However, owing to the unique correspondence between the players' actions in weakly asymmetric games, there is a natural way to extend *FMTL* beyond symmetric games. This is important because, in an asymmetric game, it is no longer necessarily true that equal outcomes are necessarily "fair."

Another way of thinking about the objective $V_F(\mathbf{p}, \mathbf{q}) = -|\pi_X(\mathbf{p}, \mathbf{q}) - \pi_Y(\mathbf{p}, \mathbf{q})|$ is in terms of the taxicab distance between $(\pi_Y(\mathbf{p}, \mathbf{q}), \pi_X(\mathbf{p}, \mathbf{q}))$ and $(\pi_Y(\mathbf{q}, \mathbf{p}), \pi_X(\mathbf{q}, \mathbf{p}))$ in $\mathbb{R}^2$. Note that when X and Y swap strategies, they implicitly take into account the correspondence $\phi_Y^{-1} \circ \phi_X$. In other words, if $\mathbf{q}$ says that Y plays y with probability q, then when X plays $\mathbf{q}$ she plays $\phi_X^{-1} \circ \phi_Y(y)$ with probability q. When the game is weakly asymmetric, we can let

$$V_F(\mathbf{p}, \mathbf{q}) = -\frac{1}{2}|\pi_X(\mathbf{p}, \mathbf{q}) - \pi_X(\mathbf{q}, \mathbf{p})| - \frac{1}{2}|\pi_Y(\mathbf{p}, \mathbf{q}) - \pi_Y(\mathbf{q}, \mathbf{p})| \tag{S32}$$

serve as a proxy for measuring fairness. The idea is to allow X and Y to swap behaviors and then measure how far away the resulting points in payoff space are from one another. The taxicab metric is not special; we simply choose it here (and in the definition of *FMTL* in general) for convenience. In symmetric games, this objective V_F reduces to the one studied in the main text.

Returning to the asymmetric donation game, X receives $b_Y - c$ and Y receives $b_X - c$ when both players cooperate in the iterated game. For two such strategies, $\mathbf{p}$ for X and $\mathbf{q}$ for Y, their payoffs do not change if they swap strategies with one another, so the unequal outcome of $b_Y - c$ for X and $b_X - c$ for Y is considered fair. This makes intuitive sense because both players are taking the same action ("donate") and making the same sacrifice (paying c). More generally, $V_F(\mathbf{p}, \mathbf{q}) = 0$ if and only if $\pi_X(\mathbf{p}, \mathbf{q}) = \pi_X(\mathbf{q}, \mathbf{p})$ and $\pi_Y(\mathbf{p}, \mathbf{q}) = \pi_Y(\mathbf{q}, \mathbf{p})$. For this asymmetric donation game, these equations hold if and only if there exists K such that $\pi_X(\mathbf{p}, \mathbf{q}) = K(b_Y - c)$ and $\pi_Y(\mathbf{p}, \mathbf{q}) = K(b_X - c)$. As a result, the line connecting the payoffs for mutual defection and mutual cooperation represent the fairest possible outcomes.

For measuring efficiency, we retain the team payoff, $V_E(\mathbf{p}, \mathbf{q}) = \pi_X(\mathbf{p}, \mathbf{q}) + \pi_Y(\mathbf{p}, \mathbf{q})$. The associated learning rule, which we denote *FMTL-WA* ("WA" for "weakly asymmetric") is defined analogously to *FMTL* in the main text: first decide which of V_F and V_E to use, based (stochastically) on the value of V_F; then sample in order to optimize the chosen objective function. Figure S13 shows the results of three learning rules against a selfish learner in an asymmetric donation game. The first learner is itself a selfish learner; the second uses the (symmetric) version of *FMTL* described in the main text; and the third is *FMTL-WA*. Although *FMTL* itself

performs much better against a selfish learner than does another selfish learner, it is *FMTL-WA* that is able to bring out optimal payoffs in more than 99% of runs. Note that the dashed line in Figure S13 no longer represents equality but rather fairness as defined by $V_F = 0$ (see above).

References

[1] R. Axelrod. *The Evolution of Cooperation*. Basic Books, 1984.

[2] M. A. Nowak. *Evolutionary Dynamics: Exploring the Equations of Life*. Belknap Press, 2006.

[3] K. Sigmund. *The calculus of selfishness*. Princeton University Press, 2010.

[4] C. Hilbe, K. Chatterjee, and M. A. Nowak. Partners and rivals in direct reciprocity. *Nature Human Behaviour*, 2018. doi: 10.1038/s41562-018-0320-9.

[5] P. Dal Bó and G. R. Fréchette. On the Determinants of Cooperation in Infinitely Repeated Games: A Survey. *Journal of Economic Literature*, 56(1):60–114, 2018. doi: 10.1257/jel.20160980.

[6] R. Axelrod and W. Hamilton. The evolution of cooperation. *Science*, 211(4489):1390–1396, 1981. doi: 10.1126/science.7466396.

[7] P. Molander. The optimal level of generosity in a selfish, uncertain environment. *Journal of Conflict Resolution*, 29:611–618, 1985.

[8] D. P. Kraines and V. Y. Kraines. Pavlov and the prisoner's dilemma. *Theory and Decision*, 26:47–79, 1989.

[9] M. A. Nowak and K. Sigmund. Tit for tat in heterogeneous populations. *Nature*, 355: 250–253, 1992.

[10] M. Nowak and K. Sigmund. A strategy of win-stay, lose-shift that outperforms tit-for-tat in the Prisoner's Dilemma game. *Nature*, 364(6432):56–58, 1993. doi: 10.1038/364056a0.

[11] C. Hauert and H. G. Schuster. Effects of increasing the number of players and memory size in the iterated prisoner's dilemma: a numerical approach. *Proceedings of the Royal Society B*, 264:513–519, 1997.

[12] I. Fischer, A. Frid, S. J. Goerg, S. A. Levin, D. I. Rubenstein, and R. Selten. Fusing enacted and expected mimicry generates a winning strategy that promotes the evolution of cooperation. *Proceedings of the National Academy of Sciences*, 110:10229–10233, 2013.

[13] Y. Murase and S. K. Baek. Five rules for friendly rivalry in direct reciprocity. *Scientific Reports*, 10:16904, 2020.

[14] K. Lindgren. Evolutionary dynamics in game-theoretic models. In W. B. Arthur, Steven N. Durlauf, and David A. Lane, editors, *The Economy as an Evolving Complex System II*, pages 337–368. Addison-Wesley, Reading MA, 1997.

[15] G. Szabó, T. Antal, P. Szabó, and M. Droz. Spatial evolutionary prisoner's dilemma game with three strategies and external constraints. *Physical Review E*, 62:1095–1103, 2000.

[16] M. A. Nowak, A. Sasaki, C. Taylor, and D. Fudenberg. Emergence of cooperation and evolutionary stability in finite populations. *Nature*, 428(6983):646–650, 2004. doi: 10.1038/nature02414.

[17] L. A. Imhof and M. A. Nowak. Stochastic evolutionary dynamics of direct reciprocity. *Proceedings of the Royal Society B*, 277:463–468, 2010.

[18] L. A. Martinez-Vaquero, J. A. Cuesta, and A. Sanchez. Generosity pays in the presence of direct reciprocity: A comprehensive study of 2x2 repeated games. *PLoS ONE*, 7(4): E35135, 2012.

[19] A. Szolnoki and M. Perc. Defection and extortion as unexpected catalysts of unconditional cooperation in structured populations. *Scientific Reports*, 4:5496, 2014.

[20] S. K. Baek, H.-C. Jeong, C. Hilbe, and M. A. Nowak. Comparing reactive and memory-one strategies of direct reciprocity. *Scientific Reports*, 6(1), 2016. doi: 10.1038/srep25676.

[21] R. Boyd and J.M.D. Lorberbaum. No pure strategy is evolutionary stable in the iterated prisoner's dilemma game. *Nature*, 327:58–59, 1987.

[22] R. Boyd. Mistakes allow evolutionary stability in the repeated Prisoner's Dilemma game. *Journal of Theoretical Biology*, 136:47–56, 1989.

[23] J. García and M. van Veelen. In and out of equilibrium I: Evolution of strategies in repeated games with discounting. *Journal of Economic Theory*, 161:161–189, 2016.

[24] J. García and M. van Veelen. No strategy can win in the repeated prisoner's dilemma: Linking game theory and computer simulations. *Frontiers in Robotics and AI*, 5:102, 2018.

[25] M. Milinski and C. Wedekind. Working memory constrains human cooperation in the prisoner's dilemma. *Proceedings of the National Academy of Sciences USA*, 95:13755–13758, 1998.

[26] P. Dal Bó and G. R. Fréchette. The Evolution of Cooperation in Infinitely Repeated Games: Experimental Evidence. *American Economic Review*, 101(1):411–429, 2011. doi: 10.1257/aer.101.1.411.

[27] D. Fudenberg, A. Dreber, and D. G. Rand. Slow to anger and fast to forgive: Cooperation in an uncertain world. *American Economic Review*, 102:720–749, 2012.

[28] C. Hilbe, T. Röhl, and M. Milinski. Extortion subdues human players but is finally punished in the prisoner's dilemma. *Nature Communications*, 5:3976, 2014.

[29] N. J. Raihani and R. Bshary. Resolving the iterated prisoner's dilemma: theory and reality. *Journal of Evolutionary Biology*, 24:1628–1639, 2011.

[30] J. M. Pacheco, F. C. Santos, M. O. Souza, and B. Skyrms. Evolutionary dynamics of collective action in n-person stag hunt dilemmas. *Proceedings of the Royal Society B*, 276: 315–321, 2009.

[31] F. C. Santos and J. M. Pacheco. Risk of collective failure provides an escape from the tragedy of the commons. *Proceedings of the National Academy of Sciences USA*, 108: 10421–10425, 2011.

[32] S. Iyer and T. Killingback. Evolution of cooperation in social dilemmas on complex networks. *PLoS Computational Biology*, 12(2):e1004779, 2016.

[33] M. C. Couto, J. M. Pacheco, and F. C. Santos. Governance of risky public goods under graduated punishment. *Journal of Theoretical Biology*, 505:110423, 2020.

[34] W. H. Press and F. J. Dyson. Iterated Prisoner's Dilemma contains strategies that dominate any evolutionary opponent. *Proceedings of the National Academy of Sciences*, 109(26): 10409–10413, 2012. doi: 10.1073/pnas.1206569109.

[35] A. J. Stewart and J. B. Plotkin. From extortion to generosity, evolution in the Iterated Prisoner's Dilemma. *Proceedings of the National Academy of Sciences*, 110(38):15348–15353, 2013. doi: 10.1073/pnas.1306246110.

[36] A. J. Stewart and J. B. Plotkin. Collapse of cooperation in evolving games. *Proceedings of the National Academy of Sciences USA*, 111(49):17558 – 17563, 2014.

[37] C. Hilbe, A. Traulsen, and K. Sigmund. Partners or rivals? Strategies for the iterated prisoner's dilemma. *Games and Economic Behavior*, 92:41–52, 2015.

[38] E. Akin. What You Gotta Know to Play Good in the Iterated Prisoner's Dilemma. *Games*, 6(3):175–190, 2015. doi: 10.3390/g6030175.

[39] E. Akin. The iterated prisoner's dilemma: Good strategies and their dynamics. In I. Assani, editor, *Ergodic Theory, Advances in Dynamics*, pages 77–107. de Gruyter, Berlin, 2016.

[40] A. McAvoy and C. Hauert. Autocratic strategies for iterated games with arbitrary action spaces. *Proceedings of the National Academy of Sciences*, 113(13):3573–3578, 2016.

[41] G. Ichinose and N. Masuda. Zero-determinant strategies in finitely repeated games. *Journal of Theoretical Biology*, 438:61–77, 2018.

[42] A. Mamiya and G. Ichinose. Zero-determinant strategies under observation errors in repeated games. *Physical Review E*, 102:032115, 2020.

[43] A. McAvoy and M. A. Nowak. Reactive learning strategies for iterated games. *Proceedings of the Royal Society A: Mathematical, Physical and Engineering Sciences*, 475(2223): 20180819, 2019. doi: 10.1098/rspa.2018.0819.

[44] M. C. Boerlijst, M. A. Nowak, and K. Sigmund. Equal pay for all prisoners. *American Mathematical Monthly*, 104:303–307, 1997.

[45] S. A. H. Geritz, J. A. J. Metz, É. Kisdi, and G. Meszéna. Dynamics of adaptation and evolutionary branching. *Physical Review Letters*, 78(10):2024–2027, 1997.

[46] U. Dieckmann. Can adaptive dynamics invade? *TREE*, 12(4):128–131, 1997.

[47] A. J. Stewart and J. B. Plotkin. The evolvability of cooperation under local and non-local mutations. *Games*, 6(3):231–250, 2015.

[48] E. Fehr and K. M. Schmidt. The Economics of Fairness, Reciprocity and Altruism – Experimental Evidence and New Theories. In *Handbook of the Economics of Giving, Altruism and Reciprocity*, pages 615–691. Elsevier, 2006. doi: 10.1016/s1574-0714(06)01008-6.

[49] J. A. List. Social preferences: Some thoughts from the field. *Annual Review of Economics*, 1:563–579, 2009.

[50] I. Alger and J. W. Weibull. Evolutionary models of preference formation. *Annual Review of Economics*, 11:329–354, 2019.

[51] E. Fehr and U. Fischbacher. The nature of human altruism. *Nature*, 425:785–791, 2003.

[52] K. McAuliffe, P. R. Blake, N. Steinbeis, and F. Warneken. The developmental foundations of human fairness. *Nature Human Behaviour*, 1(2), 2017. doi: 10.1038/s41562-016-0042.

[53] W. Güth, R. Schmittberger, and B. Schwarze. An experimental analysis of ultimatum bargaining. *Journal of Economic Behavior & Organization*, 3:376–388, 1982.

[54] E. Fehr and S. Gächter. Cooperation and punishment in public goods experiments. *American Economic Review*, 90:980–994, 2000.

[55] J. Henrich, R. Boyd, S. Bowles, C. Camerer, H. Gintis, R. McElreath, and E. Fehr. In search of homo economicus: Experiments in 15 small-scale societies. *American Economic Review*, 91:73–79, 2001.

[56] B. Herrmann, C. Thöni, and S. Gächter. Antisocial punishment across societies. *Science*, 319:1362–1367, 2008.

[57] P. Blake, K. McAuliffe, J. Corbit, O. Callaghan, O. Barry, A. Bowie, L. Kleutsch, K. L. Kramer, E. Ross, H. Vongsachang, R. Wrangham, and F. Warneken. The ontogeny of fairness in seven societies. *Nature*, 528:258–261, 2015.

[58] E. Fehr, H. Bernhard, and B. Rockenbach. Egalitarianism in young children. *Nature*, 454 (7208):1079–1083, 2008.

[59] E. Tricomi, A. Rangel, C. F. Camerer, and J. P. O'Doherty. Neural evidence for inequality-averse social preferences. *Nature*, 463(7284):1089–1091, 2010. doi: 10.1038/nature08785.

[60] E. Fehr and K. M. Schmidt. A Theory of Fairness, Competition, and Cooperation. *The Quarterly Journal of Economics*, 114(3):817–868, 1999. doi: 10.1162/003355399556151.

[61] G. E. Bolton and A. Ockenfels. ERC: A theory of equity, reciprocity, and competition. *American Economic Review*, 90:166–193, 2000.

[62] J. Andreoni and J. Miller. Giving According to GARP: An Experimental Test of the Consistency of Preferences for Altruism. *Econometrica*, 70(2):737–753, 2002. doi: 10.1111/1468-0262.00302.

[63] G. Charness and M. Rabin. Understanding Social Preferences with Simple Tests. *The Quarterly Journal of Economics*, 117(3):817–869, 2002. doi: 10.1162/003355302760193904.

[64] D. Engelmann and M. Strobel. Inequality Aversion, Efficiency, and Maximin Preferences in Simple Distribution Experiments. *American Economic Review*, 94(4):857–869, 2004. doi: 10.1257/0002828042002741.

[65] W. Güth. An evolutionary approach to explaining cooperative behavior by reciprocal incentives. *International Journal of Game Theory*, 24(4):323–344, 1995. doi: 10.1007/bf01243036.

[66] W. Güth and H. Kliemt. The indirect evolutionary approach: Bridging the gap between rationality and adaptation. *Rationality and Society*, 10(3):377–399, 1998. doi: 10.1177/104346398010003005.

[67] A. Heifetz, C. Shannon, and Y. Spiegel. The Dynamic Evolution of Preferences. *Economic Theory*, 32(2):251–286, 2007. doi: 10.1007/s00199-006-0121-7.

[68] E. Akçay, J. Van Cleve, M. W. Feldman, and J. Roughgarden. A theory for the evolution of other-regard integrating proximate and ultimate perspectives. *Proceedings of the National Academy of Sciences*, 106(45):19061–19066, 2009. doi: 10.1073/pnas.0904357106.

[69] I. Alger and J. W. Weibull. Homo moralis—preference evolution under incomplete information and assortative matching. *Econometrica*, 81(6):2269–2302, 2013.

[70] Y. Shoham, R. Powers, and T. Grenager. If multi-agent learning is the answer, what is the question? *Artificial Intelligence*, 171(7):365–377, May 2007. doi: 10.1016/j.artint.2006.02.006.

[71] K. Tuyls and S. Parsons. What evolutionary game theory tells us about multiagent learning. *Artificial Intelligence*, 171(7):406–416, 2007. doi: 10.1016/j.artint.2007.01.004.

[72] D. Bloembergen, K. Tuyls, D. Hennes, and M. Kaisers. Evolutionary Dynamics of Multi-Agent Learning: A Survey. *Journal of Artificial Intelligence Research*, 53:659–697, Aug 2015. doi: 10.1613/jair.4818.

[73] K. Zhang, Z. Yang, and T. Başar. *Handbook of Reinforcement Learning and Control*, chapter Multi-Agent Reinforcement Learning: A Selective Overview of Theories and Algorithms, pages 321–384. Springer International Publishing, 2021. doi: 10.1007/978-3-030-60990-0_12.

[74] K. Tuyls and G. Weiss. Multiagent Learning: Basics, Challenges, and Prospects. *AI Magazine*, 33(3):41, Sep 2012. doi: 10.1609/aimag.v33i3.2426.

[75] K. Tuyls, P. J. 'T Hoen, and B. Vanschoenwinkel. An Evolutionary Dynamical Analysis of Multi-Agent Learning in Iterated Games. *Autonomous Agents and Multi-Agent Systems*, 12 (1):115–153, 2005. doi: 10.1007/s10458-005-3783-9.

[76] J. Foerster, R. Y. Chen, M. Al-Shedivat, S. Whiteson, P. Abbeel, and I. Mordatch. Learning with opponent-learning awareness. In *Proceedings of the 17th International Conference on Autonomous Agents and MultiAgent Systems*, pages 122–130. International Foundation for Autonomous Agents and Multiagent Systems, 2018.

[77] D. Silver, A. Huang, C. J. Maddison, A. Guez, L. Sifre, G. Van Den Driessche, J. Schrittwieser, I. Antonoglou, V. Panneershelvam, M. Lanctot, et al. Mastering the game of Go with deep neural networks and tree search. *Nature*, 529(7587):484, 2016.

[78] D. Silver, J. Schrittwieser, K. Simonyan, I. Antonoglou, A. Huang, A. Guez, T. Hubert, L. Baker, M. Lai, A. Bolton, et al. Mastering the game of Go without human knowledge. *Nature*, 550(7676):354–359, 2017.

[79] Oriol Vinyals, Igor Babuschkin, Wojciech M Czarnecki, Michaël Mathieu, Andrew Dudzik, Junyoung Chung, David H Choi, Richard Powell, Timo Ewalds, Petko Georgiev, et al. Grandmaster level in StarCraft II using multi-agent reinforcement learning. *Nature*, 575 (7782):350–354, 2019.

[80] Joel Z Leibo, Vinicius Zambaldi, Marc Lanctot, Janusz Marecki, and Thore Graepel. Multi-agent reinforcement learning in sequential social dilemmas. *arXiv preprint arXiv:1702.03037*, 2017.

[81] M. Bowling and M. Veloso. Multiagent learning using a variable learning rate. *Artificial Intelligence*, 136(2):215–250, Apr 2002. doi: 10.1016/s0004-3702(02)00121-2.

[82] J. Hu and M. P. Wellman. Nash Q-learning for general-sum stochastic games. *Journal of Machine Learning Research*, 4:1039–1069, 2003.

[83] D. Ivanov, V. Egorov, and A. Shpilman. *Balancing Rational and Other-Regarding Preferences in Cooperative-Competitive Environments*, page 15361538. International Foundation for Autonomous Agents and Multiagent Systems, 2021.

[84] A. Peysakhovich and A. Lerer. Prosocial Learning Agents Solve Generalized Stag Hunts Better than Selfish Ones. In *Proceedings of the 17th International Conference on Autonomous Agents and MultiAgent Systems*, page 20432044. International Foundation for Autonomous Agents and Multiagent Systems, 2018.

[85] E. Hughes, J. Z. Leibo, M. Phillips, K. Tuyls, E. A. Duéñez-Guzmán, A. G. Castañeda, I. Dunning, T. Zhu, K. R. McKee, R. Koster, H. Roff, and T. Graepel. Inequity aversion improves cooperation in intertemporal social dilemmas. In *Advances in Neural Information Processing Systems*, pages 3330–3340, 2018.

[86] J. Maynard Smith and G. R. Price. The Logic of Animal Conflict. *Nature*, 246(5427): 15–18, 1973. doi: 10.1038/246015a0.

[87] A. Traulsen, C. Hauert, H. De Silva, M. A. Nowak, and K. Sigmund. Exploration dynamics in evolutionary games. *Proceedings of the National Academy of Sciences*, 106(3):709–712, 2009. doi: 10.1073/pnas.0808450106.

[88] G. Szabó and C. Tőke. Evolutionary Prisoner's Dilemma game on a square lattice. *Physical Review E*, 58:69–73, 1998.

[89] A. Traulsen, J. M. Pacheco, and M. A. Nowak. Pairwise comparison and selection temperature in evolutionary game dynamics. *Journal of Theoretical Biology*, 246:522–529, 2007.

[90] R. D. Luce and H. Raiffa. *Games and Decisions: Introduction and Critical Survey*. Dover Books on Mathematics. Dover Publications, 1989. ISBN 9780486659435.

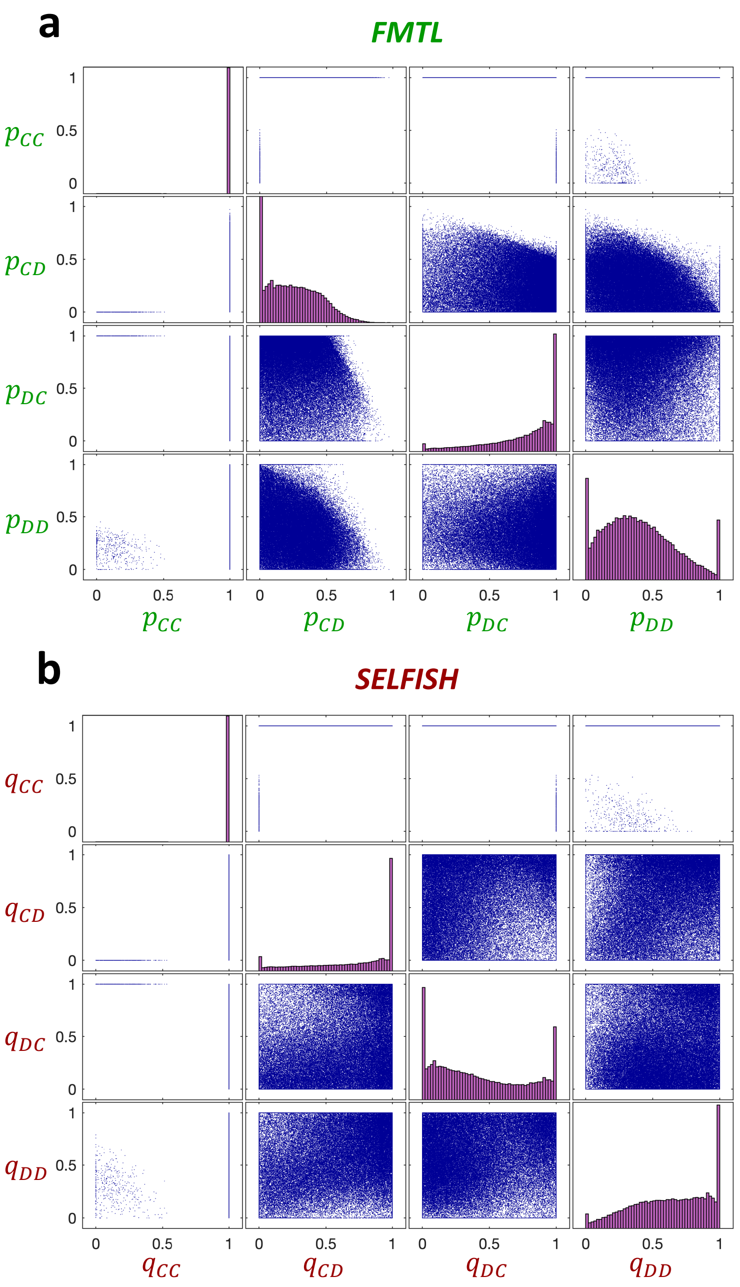

Fig. S1: Memory-one strategies resulting from *FMTL* against a selfish learner. In the donation game with $b = 2$ and $c = 1$, we let the learning process unfold for *FMTL* paired with a selfish learner for 10^5 randomly-chosen initial conditions. The conditional probabilities making up the resulting memory-one strategies are shown in **a** for the *FMTL* learner and in **b** for the selfish learner. Notably, the strategies that result from this learning process are quite diverse, with the simplest common feature being that $p_{CC} = q_{CC} = 1$ in most runs.

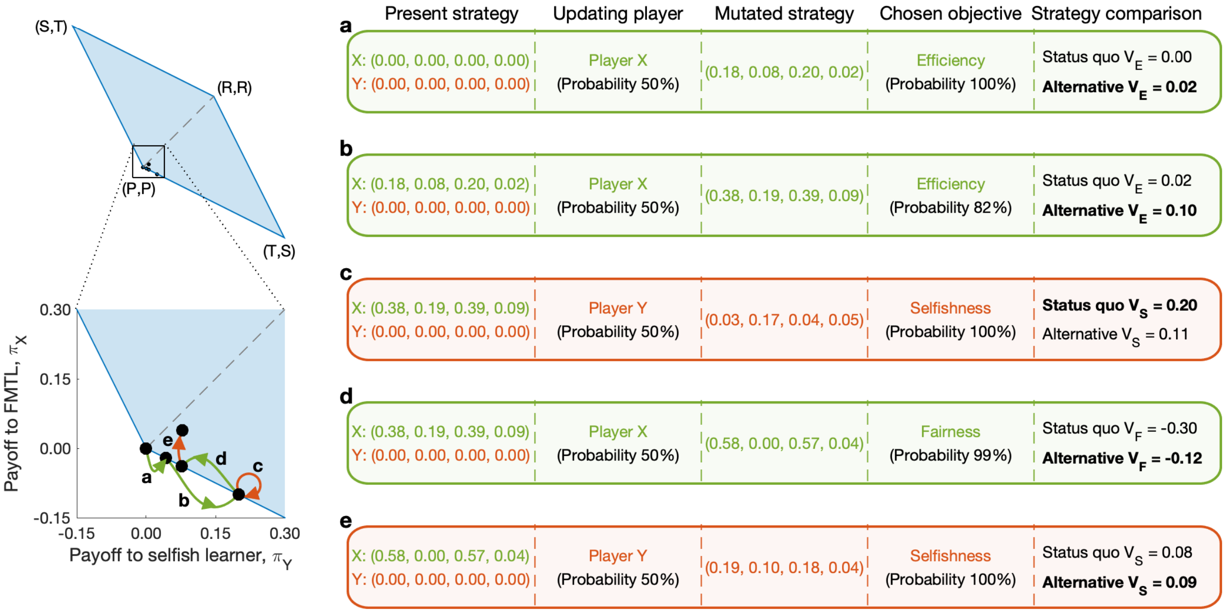

Fig. S2: *FMTL* can escape from mutual defection in the repeated donation game. We consider an interaction between an *FMTL* player (player X) and a selfish learner (player Y). Learning occurs as described in the main text. In each time step, one player is randomly chosen to revise its strategy. To this end, the respective player compares its present strategy with a nearby strategy. The players' objectives are either efficiency and fairness (for *FMTL*), or selfishness (for the selfish learner). The mutated strategy is adopted if and only if it has a better performance with respect to the objective (marked in bold). **a,b,** Initially, both players defect unconditionally. Because payoffs are equal but inefficient, the *FMTL* player consecutively adopts strategies that increase efficiency. **c,** In the meanwhile, the selfish player has no incentive yet to adapt. Unconditional defection is in the player's best interest. **d,** To avoid exploitation by the selfish player, *FMTL* regularly revises its strategy to ensure a fairer outcome. **e,** Once *FMTL* adopts a strategy that rewards cooperation sufficiently, it is also in the selfish player's interest to become more cooperative. In the end, both players are better off than initially, even if *FMTL* has a reduced payoff in the short run (during steps **a-d**). We note that the same sequence of updating players and mutated strategies would have had no effect if both players were selfish learners. Parameters: We consider a donation game with $b = 2$ and $c = 1$ without discounting ($\lambda = 1$). The depicted memory-one strategies are of the form $(p_{CC}, p_{CD}, p_{DC}, p_{DD})$; the player's initial cooperation probability has been dropped as it is irrelevant in undiscounted games (3). To reduce the number of learning steps required for this illustration, we use a slightly larger mutation kernel: each entry of the mutated strategy can differ by at most $s = 0.2$ from the present strategy. All other parameters are the same as in Figure 3a.

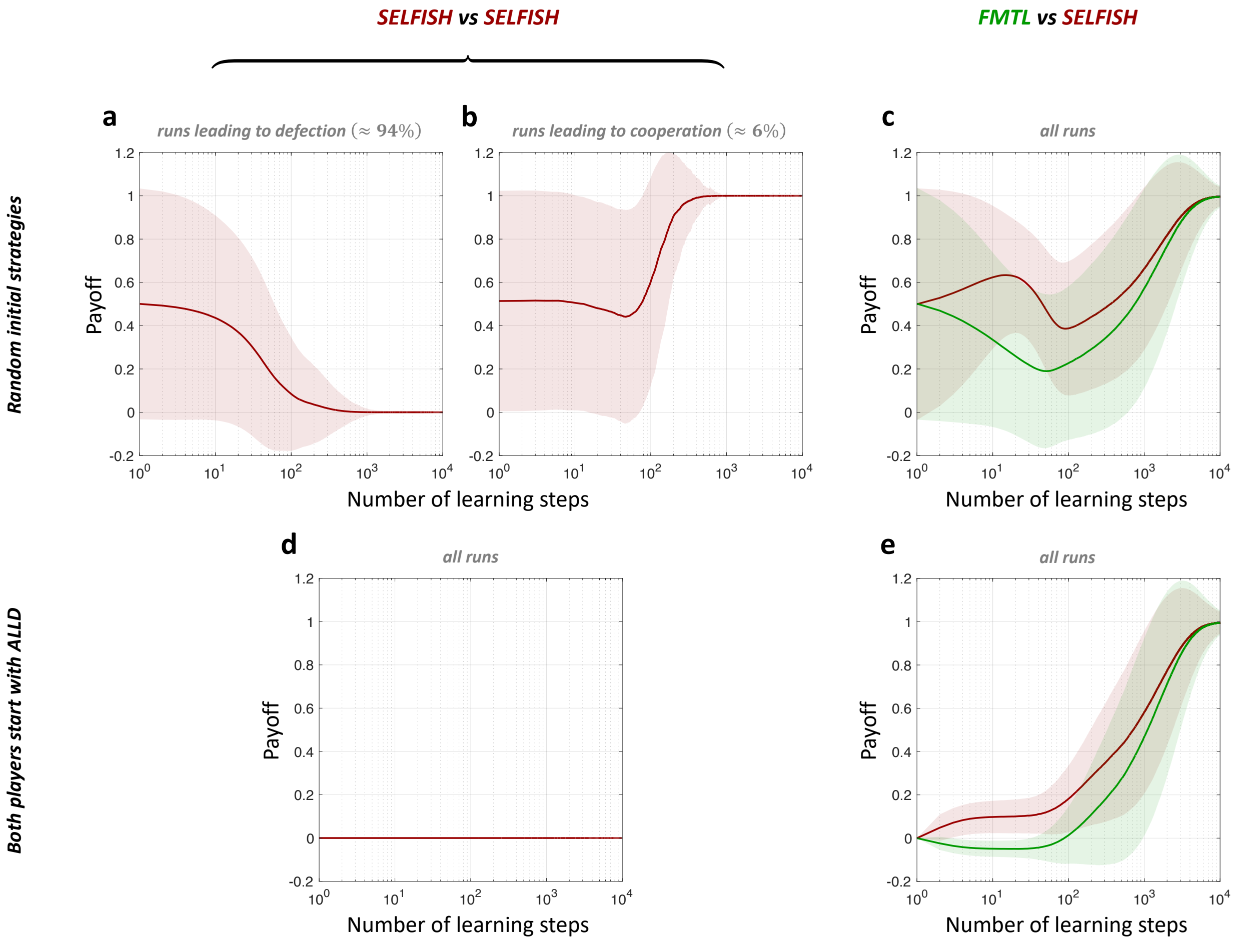

Fig. S3: Comparing trajectories of selfish learning and *FMTL*. Starting with random strategies, with each coordinate chosen independently from an arcsine distribution (**a,b,c**), or "always defect" (ALLD) (**d,e**), we observe the payoff trajectories for both learners over 10^4 time steps in the donation game with a benefit of $b = 2$ and a cost of $c = 1$. These trajectories are averaged over 10^5 initial conditions (solid lines), with shaded regions representing one standard deviation from the mean. With random initial conditions, a selfish learner against another selfish learner leads to outcomes of defection approximately 94% of the time (**a**) and outcomes of cooperation approximately 6% of the time (**b**). When *FMTL* is paired with a selfish learner, the outcome is nearly always cooperative, as *FMTL* can incentivize a selfish learner to avoid detrimental equilibria (**c**). These effects are amplified when the initial conditions are "bad," such as when both players start off at ALLD. Here, a selfish learner paired with a selfish learner can never escape defection (**d**), but *FMTL* can still destabilize defection and lead a selfish partner to a cooperative outcome (**e**).

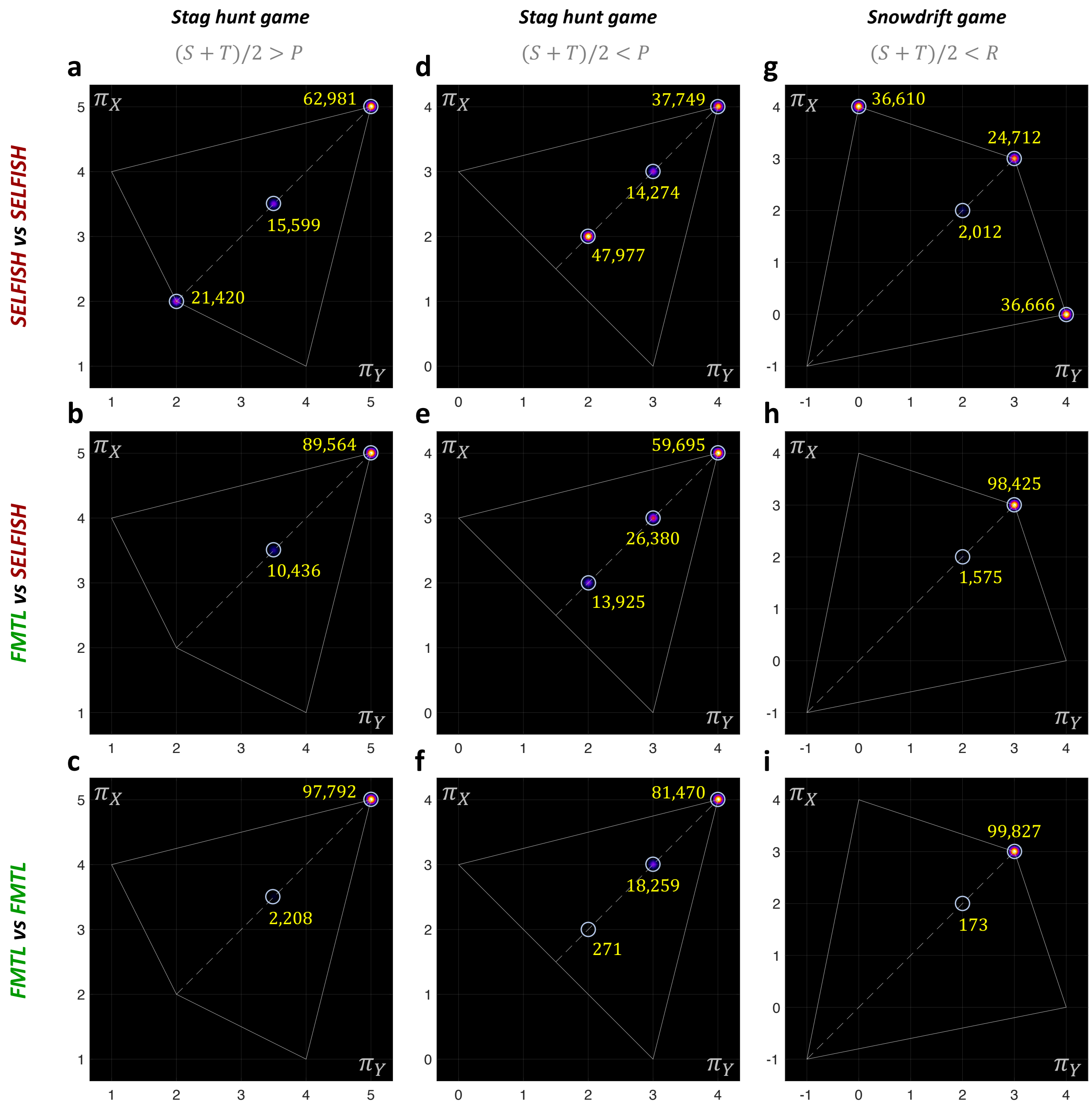

Fig. S4: Other conflicts for which selfish learning is suboptimal. In addition to the prisoner's dilemmas depicted in the main text, we consider three other classes of conflicts for which selfish learning leads to inefficient outcomes. The first two are stag hunt games, one with $(S+T)/2 > P$ (**a**,**b**,**c**) and one with $(S+T)/2 < P$ (**d**,**e**,**f**). The third is the snowdrift game with $(S+T)/2 < R$ (**g**,**h**,**i**). In both kinds of stag hunt games, *FMTL* is able to significantly improve the average payoffs of the individuals, despite not being able to ensure that all players receive an optimal payoff with certainty. In the snowdrift game, *FMTL* not only significantly increases the average payoffs; it also ensures that the two players obtain equal payoffs in all runs. The points that correspond to alternating cooperation in **h** and **i** are not actual rest points; however, escaping from these points requires more time than allowed by the termination condition (no update for 10^4 steps). Game parameters: (**a**,**b**,**c**) $R=5$, $S=1$, $T=4$, and $P=2$; (**d**,**e**,**f**) $R=4$, $S=0$, $T=3$, and $P=2$; (**g**,**h**,**i**) $R=3$, $S=0$, $T=4$, and $P=-1$.

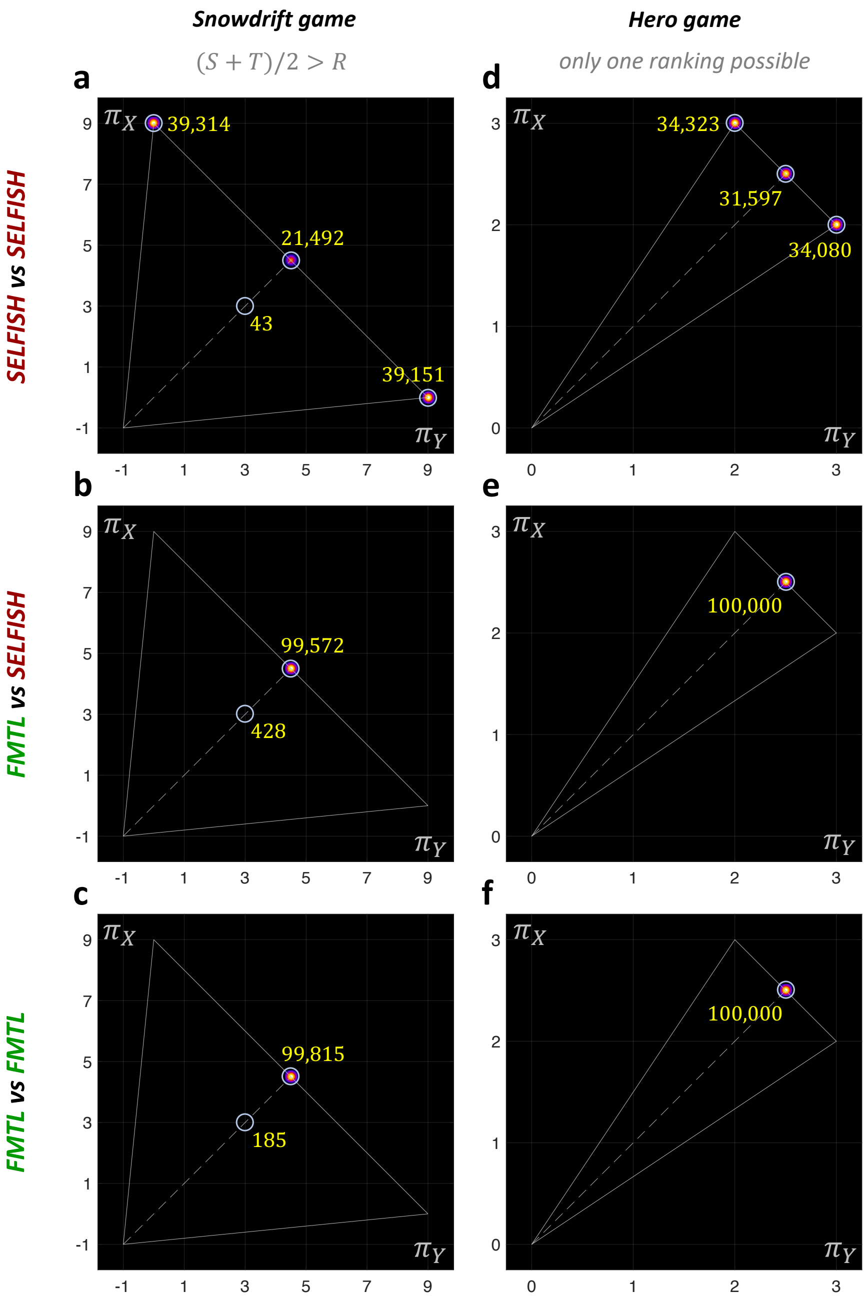

Fig. S5: Performance of *FMTL* in conflicts for which selfish learning is nearly optimal. In the snowdrift game with $(S+T)/2 > R$ (**a**,**b**,**c**) and the hero game (**g**,**h**,**i**), a selfish learner against a selfish learner leads to nearly optimal outcomes on average (**a** and **d**). For the snowdrift game, *FMTL* versus a selfish learner actually leads to slightly lower average payoffs due to the insistence on fairness, which can lead (by chance) to a small amount of additional runs ending at mutual cooperation. For the hero game, the average payoffs in **d**,**e**,**f** are the same since the game is undiscounted. (Otherwise, the initial few rounds needed to coordinate a policy of alternation can affect payoffs slightly, but even in this case the average payoffs in **d**,**e**,**f** are approximately equal.) Game parameters: (**a**,**b**,**c**) $R=3$, $S=0$, $T=9$, and $P=-1$; (**d**,**e**,**f**) $R=1$, $S=3$, $T=2$, and $P=0$.

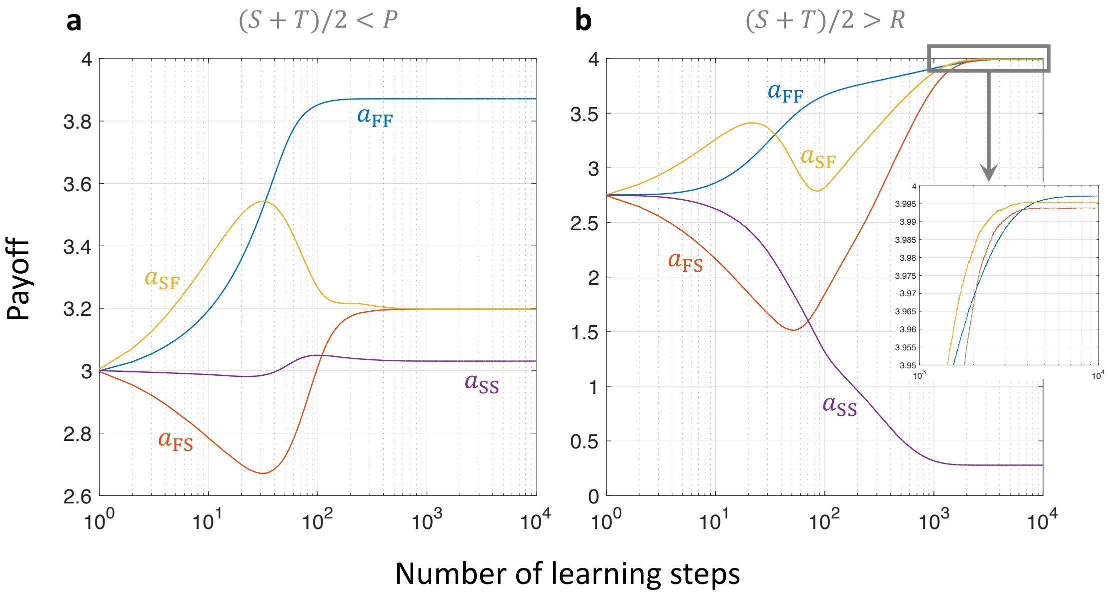

Fig. S6: Supergame payoffs in non-standard prisoner's dilemma interactions. The donation game is a prisoner's dilemma satisfying $P < (S+T)/2 < R$. Here, we instead consider prisoner's dilemmas in which one of these two inequalities does not hold. (There is no prisoner's dilemma in which both inequalities fail to hold.) In **a**, alternating cooperation is the most inefficient outcome, whereas in **b**, alternating cooperation is most efficient outcome. In both cases, the learning process eventually turns the resulting supergame into a harmony game, which means that *FMTL* can invade and replace a population of selfish learners. In **a,** this happens after $\approx$ 100 learning steps (when a_{FS} exceeds a_{SS}). In **b,** it happens slightly earlier. Game parameters: **a,** $R = 4$, $S = 0$, $T = 5$, and $P = 3$; **b,** $R = 3$, $S = -1$, $T = 9$, and $P = 0$.

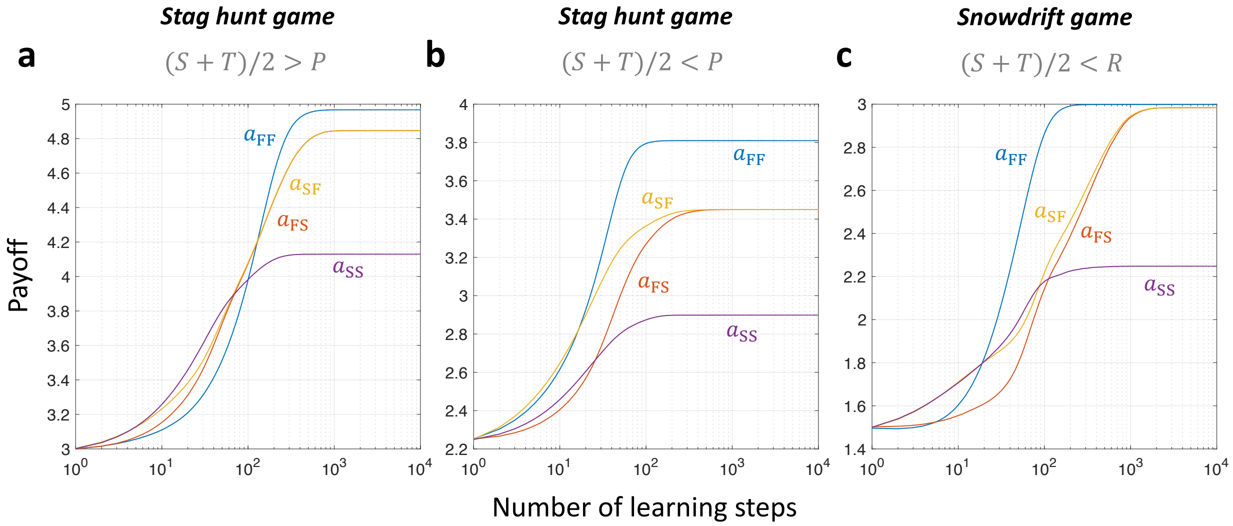

Fig. S7: Supergame payoffs in conflicts for which selfish learning is suboptimal. In each of these games, mutual selfish learning leads to relatively low payoffs (purple). *FMTL* is able to greatly improve these payoffs, on average. As a result, after a reasonably small number of learning steps, *FMTL* globally dominates selfish learning in the resulting evolutionary dynamics. Game parameters: **a** $R = 5$, $S = 1$, $T = 4$, and $P = 2$; **b** $R = 4$, $S = 0$, $T = 3$, and $P = 2$; **c** $R = 3$, $S = 0$, $T = 4$, and $P = -1$.

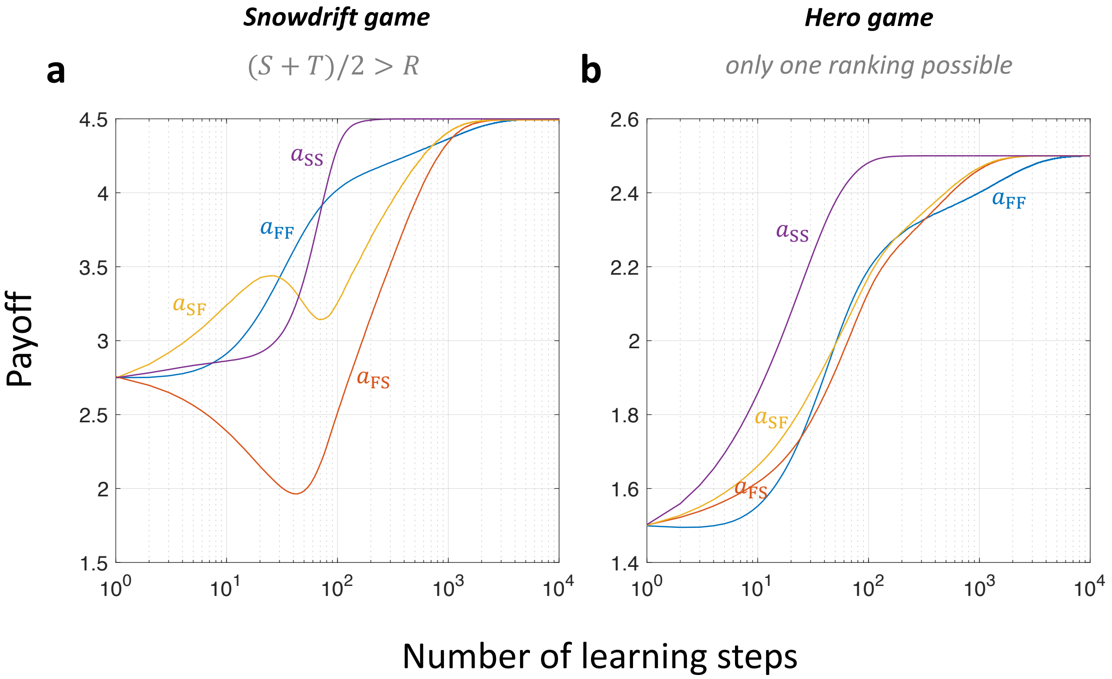

Fig. S8: Supergame payoffs in conflicts for which selfish learning is nearly optimal. Since selfish learning is already nearly optimal (on average) in the snowdrift and hero games, *FMTL* is approximately neutral relative to selfish learning after sufficiently many learning steps. Since *FMTL* also values fairness (which in these cases does not affect evolutionary dynamics), it is less efficient in converging to a final outcome. As a result, on shorter learning timescales, selfish learning is significantly favored relative to *FMTL*. Moreover, since *FMTL* picks up a small number of additional suboptimal points at mutual cooperation in the snowdrift game (Figure S5**b,c**), after a sufficiently long learning horizon we find that there is bistable competition between *FMTL* and selfish learning. However, since the long-run payoffs of the four curves in each of **a** and **b** all converge to approximately the same value, any competition between the two learning rules is nearly neutral after enough learning steps. Moreover, unlike in the previous conflicts we have studied, competition between the two learners in these two games does not significantly change the payoffs in a population. Game parameters: **a** $R = 3$, $S = 0$, $T = 9$, and $P = -1$; **b** $R = 1$, $S = 3$, $T = 2$, and $P = 0$.

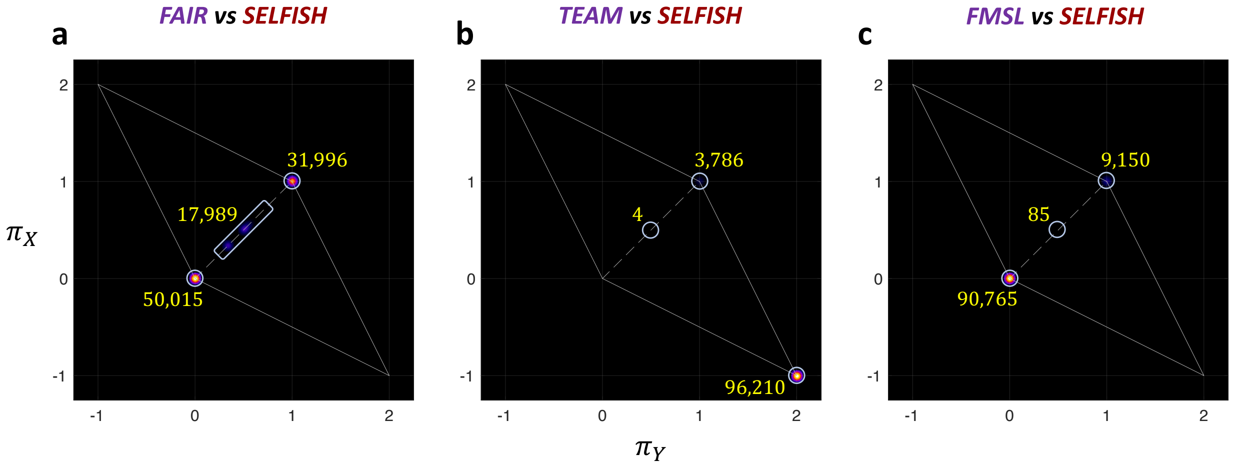

Fig. S9: Performance of related learning rules. Since *FMTL* balances both fairness and team-based learning, a natural question is whether either of these two objectives alone is sufficient when paired with a selfish learner. We consider the donation game with $b = 2$ and $c = 1$ as an example. **a** and **b** demonstrate that neither one alone can reliably lead to good outcomes. The final outcomes in **a** are fair but often involve a substantial amount of defection. In **b**, a selfish learner exploits a team-based learner in most runs. Finally, **c** addresses the question of whether good outcomes are possible with fairness-mediated *selfish* learning (*FMSL*). The results are reminiscent of when a selfish learner is paired with another selfish learner; in particular, the majority of outcomes from *FMSL* versus a selfish learner involve a substantial amount of defection. The endpoints in all panels are based on 10^5 random initial strategy pairs.

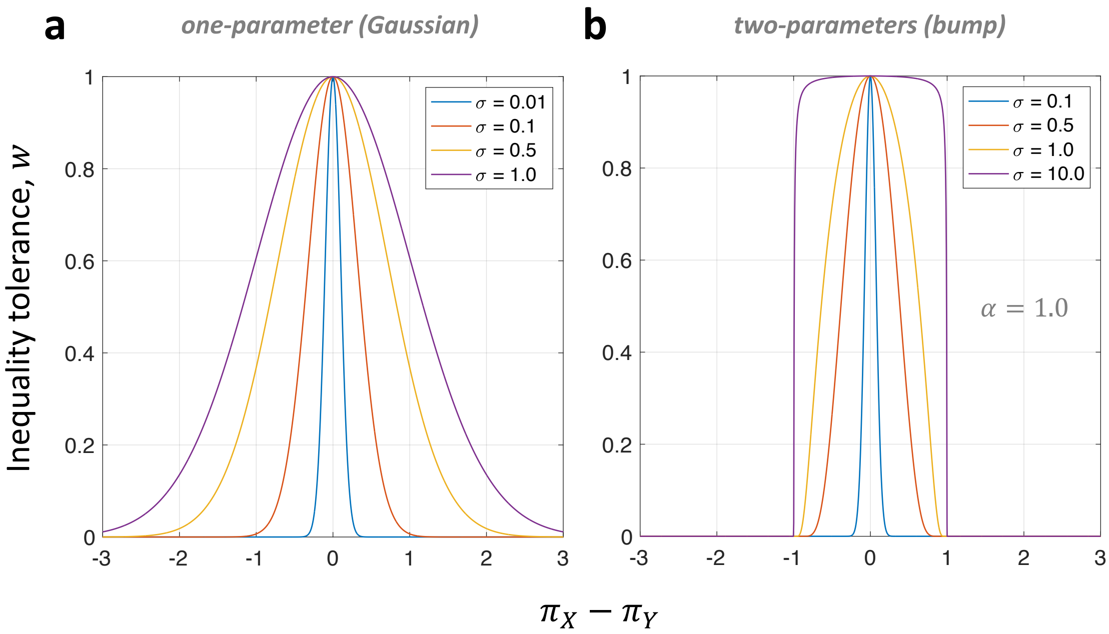

Fig. S10: Inequality tolerance functions for *FMTL*. Suppose that X and Y use strategies $\mathbf{p}$ and $\mathbf{q}$, respectively. In the *FMTL* rule, ω represents the probability that the learner maximizes the sum of the players' payoffs, $\pi_X(\mathbf{p},\mathbf{q}) + \pi_Y(\mathbf{p},\mathbf{q})$, as opposed to minimizing the magnitude of the difference, $|\pi_X(\mathbf{p},\mathbf{q}) - \pi_Y(\mathbf{p},\mathbf{q})|$. This probability is a function of the current value of $|\pi_X(\mathbf{p},\mathbf{q}) - \pi_Y(\mathbf{p},\mathbf{q})|$. **a** depicts a Gaussian tolerance function, which involves only one parameter (see Equation **5**). **b** shows another option, a bump function, which allows for more control over both the rate of decay and the support of the function. This flexibility comes at the added cost of an additional parameter (see Equation **6**). In our examples, we do not see significant qualitative differences between the two functions when implemented in *FMTL*, so we use the Gaussian function for simplicity.

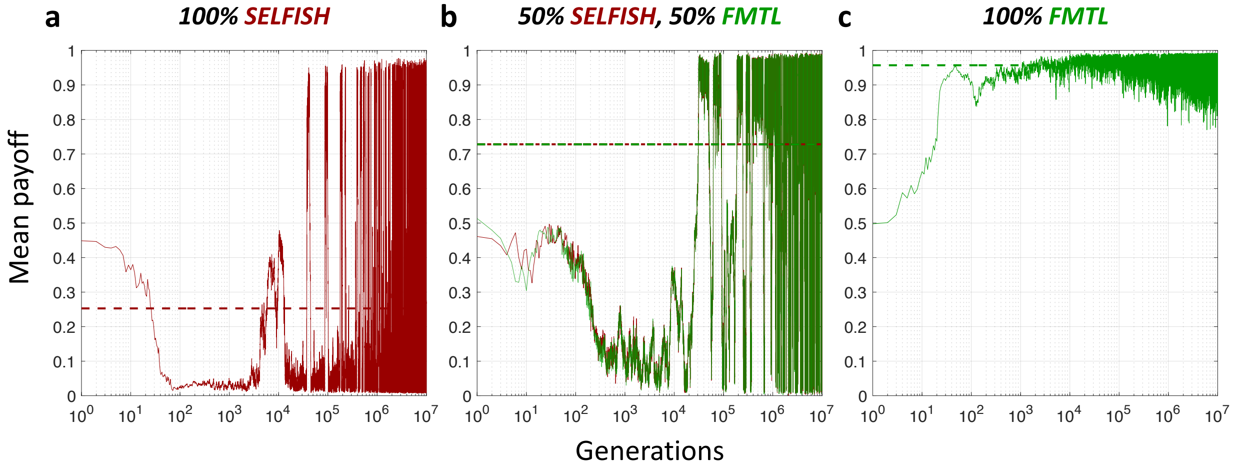

Fig. S11: Imitation-based learning dynamics. In a population of size $N = 100$, we consider synchronous imitation dynamics based on different social preferences. At the start, each individual chooses a random memory-one strategy, with each coordinate chosen independently from an arcsine distribution. In **a**, all individuals imitate based on a desire to improve their own payoffs. Panels **b** and **c** illustrate the presence of *FMTL* imitators, who imitate based on either efficiency or fairness, with an equal mixture of selfish and *FMTL* in **b** and only *FMTL* in **c**. In each, mean payoffs over 10^7 generations are depicted using dashed lines. Notably, these mean payoffs increase with the presence of *FMTL*.

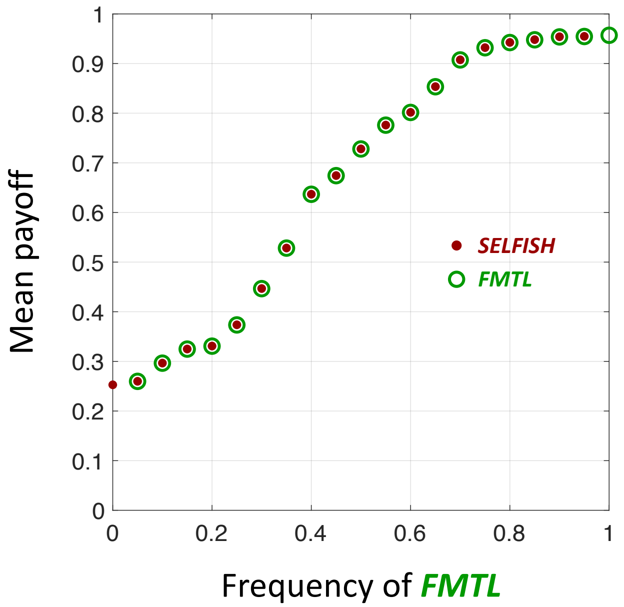

Fig. S12: Mean payoffs as functions of *FMTL* frequency under imitation dynamics. In a population of size $N = 100$, mean payoffs over 10^7 generations are shown in red for selfish imitators and in green for *FMTL* imitators (corresponding to the dashed lines in Figure S11). The number of *FMTL* present in the population ranges from 0 to N in increments of 5, and the trend is that *FMTL* increases the mean payoffs of both kinds of imitators. In particular, a selfish imitator would always benefit from switching to *FMTL*, which is qualitatively similar to the findings for introspection dynamics in the main text. The stochasticity in these mean payoffs is due to finite population effects and the choice of a finite number of generations (10^7) over which to average.

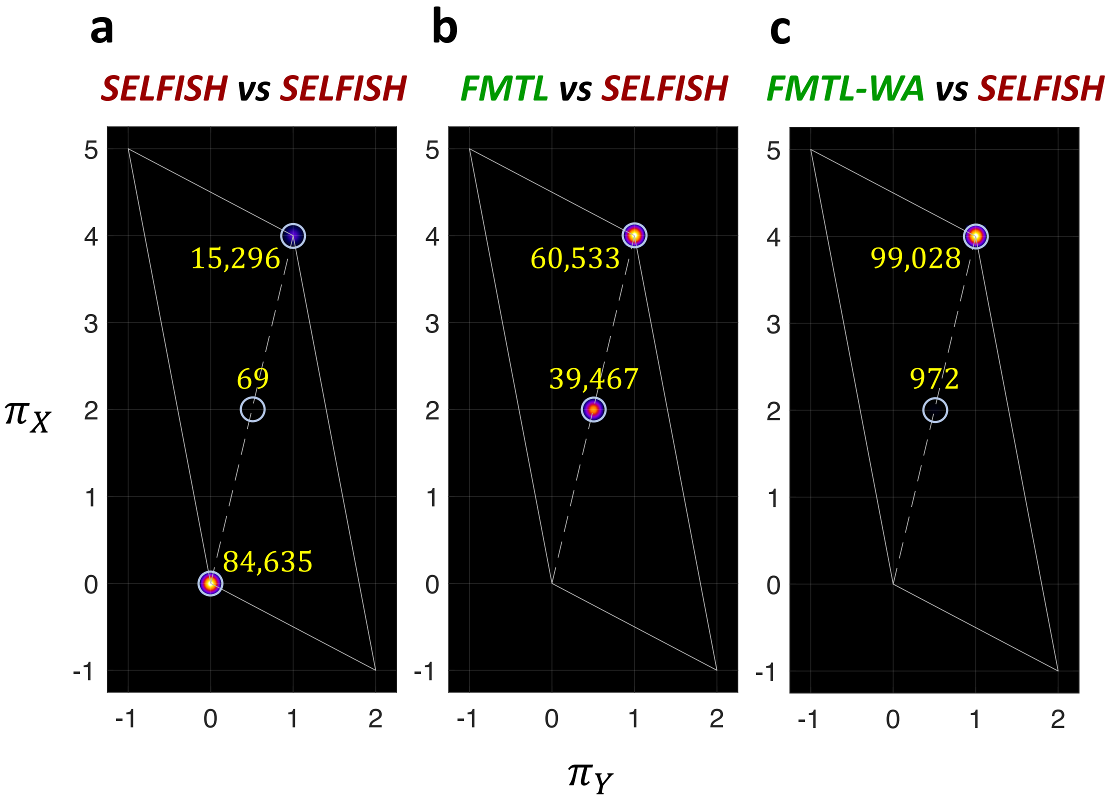

Fig. S13: Fairness-mediated team learning in a weakly-asymmetric game. The learning rule *FMTL*, if not properly modified for asymmetric games, may be thought of as "equality-mediated team learning." Although this learning rule results in better outcomes against a selfish learner relative to selfish learning (panels **a** and **b**), it does not properly take into account fairness since equality no longer serves as a proxy for fairness in asymmetric settings. Instead, since this asymmetric donation game is weakly asymmetric, there is a natural correspondence between the strategies of the two players (which, in this case, is just C to C and D to D). This correspondence allows the players to compare payoffs when strategies are swapped (Equation **S32**), giving rise to a weakly-asymmetric version of *FMTL*, which we denote *FMTL-WA* (panel **c**). This rule elicits fair, optimal outcomes against a selfish learner in over 99% of runs, reflecting the qualitative findings discussed for symmetric games in the main text. The dashed line represents the outcomes that are perfectly fair, i.e. which maximize the objective function in Equation **S32**. The endpoints in each panel are based on 10^5 randomly-sampled initial strategies, with $b_X = 2$, $b_Y = 5$, and $c = 1$.

Game	Variant	Fraction of simulations converging to **approximately efficient outcome**: *Selfish Learning* vs. *Selfish Learning*	Fraction of simulations converging to **approximately efficient outcome**: *FMTL* vs. *Selfish Learning*	Fraction of simulations converging to **approximately fair outcome**: *Selfish Learning* vs. *Selfish Learning*	Fraction of simulations converging to **approximately fair outcome**: *FMTL* vs. *Selfish Learning*
**Prisoner's dilemma** $T > R > P > S$	$R + P = S + T$ (**Figure 2**)	*6 %*	*> 99 %*	*> 99 %*	*> 99 %*
	$(S + T)/2 < P$ (**Figure 5a**)	*3 %*	*20 %*	*> 99 %*	*> 99 %*
	$(S + T)/2 > R$ (**Figure 3**)	*5 %*	*> 99 %*	*> 99 %*	*> 99 %*
**Stag hunt game** $R > T > P > S$	$(S + T)/2 > P$ (**Supp. Figure 4**)	*63 %*	*90 %*	*> 99 %*	*> 99 %*
	$(S + T)/2 < P$ (**Supp. Figure 4**)	*38 %*	*60 %*	*> 99 %*	*> 99 %*
**Snowdrift game** $T > R > S > P$	$(S + T)/2 < R$ (**Supp. Figure 4**)	*25 %*	*98 %*	*27 %*	*> 99 %*
	$(S + T)/2 > R$ (**Supp. Figure 5**)	*> 99 %*	*> 99 %*	*22 %*	*> 99 %*
**Hero game** $S > T > R \geq P$	*Only one possible ranking* (**Supp. Figure 5**)	*> 99 %*	*> 99 %*	*32 %*	*> 99 %*

Tab. S1: Performance of learning rules across different repeated games. When facing a selfish learner, we ask, for each learning rule, whether the resulting dynamics eventually lead to efficiency and fairness. For efficiency, we consider the degree to which a learner can evade all inefficient equilibria. For fairness, we ask whether players are expected to achieve an equitable outcome. We note that in games in which alternating cooperation is particularly detrimental, such that $(S + T)/2 < P$, no combination of learning rules can escape from mutual defection. However, if at least one player adopts *FMTL*, players are much less likely to settle at mutual defection in the first place (Figure S6a, Figure S4d,e, and Figure S7b).

<table>
<tr><th></th><th></th><th colspan="3">Learning horizon</th></tr>
<tr><th>Game</th><th>Variant</th><th>Short</th><th>Intermediate</th><th>Long</th></tr>
<tr><td rowspan="3">Prisoner's dilemma
$T > R > P > S$</td><td>$R + P = S + T$
(Figure 4a)</td><td rowspan="8">SELFISH globally dominates FMTL</td><td rowspan="2">Bistable competition</td><td rowspan="6">FMTL globally dominates SELFISH</td></tr>
<tr><td>$(S + T)/2 < P$
(Figure 5a)</td></tr>
<tr><td>$(S + T)/2 > R$
(Figure 5b)</td><td>Multiple dynamics</td></tr>
<tr><td rowspan="2">Stag hunt game
$R > T > P > S$</td><td>$(S + T)/2 > P$
(Supp. Figure 6a)</td><td>Coexistence</td></tr>
<tr><td>$(S + T)/2 < P$
(Supp. Figure 6b)</td><td rowspan="3">Bistable competition</td></tr>
<tr><td rowspan="2">Snowdrift game
$T > R > S > P$</td><td>$(S + T)/2 < R$
(Supp. Figure 6c)</td></tr>
<tr><td>$(S + T)/2 > R$
(Supp. Figure 7a)</td><td rowspan="2">Nearly-neutral competition</td></tr>
<tr><td>Hero game
$S > T > R \geq P$</td><td>Only one possible ranking
(Supp. Figure 7b)</td><td>Multiple dynamics</td></tr>
</table>

Tab. S2: Evolutionary dynamics among learning rules across different repeated games. Here, we summarize the results shown in Figure 4, Figure S6, Figure S7, and Figure S8. For each of the game types we consider, the table shows the qualitative dynamics between selfish learning and *FMTL*. The results depend on the players' learning horizon. The table broadly distinguishes between three cases. A short learning horizon refers to the limiting case in which players assess the performance of their learning rule based on a minimum of learning steps, for $n = 1$. A long learning horizon refers to the other limiting case in which players assess their learning rule based on which outcome they yield eventually, for $n \to \infty$. We use the intermediate class to summarize all phase transitions that we observe between those extremes. For two games, we observe multiple phase transitions. The first such game is the prisoner's dilemma with $(S + T)/2 > R$. Here, both bistable competition and coexistence are possible for intermediate learning horizons (Figure S6b). In the hero game, we observe both bistable competition and selfish learning dominating *FMTL* (Figure S8b). We note that while the short and the long class are formally defined as a limiting case, the behaviors described here are robust. In particular, selfish learning remains globally stable for $n > 1$, as long as the number of learning steps is below a threshold that depends on the game. Similarly, *FMTL* can be globally stable long before the learning dynamics converge to an equilibrium.

Vid. S1: Escape from ALLD. Both players start with strategies of ALLD, mutual unconditional defection, in the donation game with $b = 2$ and $c = 1$. X uses *FMTL* while Y is a selfish learner. After each learning step, the current payoff is depicted in purple, while the feasible region of the strategy used by X is shown in light grey. This region represents the space of all possible payoffs against this particular strategy of X. As the number of learning steps (shown in the upper-right corner) increases, X is successfully able to draw Y out of defection, eventually settling on strategies that support mutual cooperation. The feasible region for the final strategy used by X makes it clear why this strategy cannot be exploited: any deviation in payoffs must decrease the score of Y.
